# Supplementary material for: Anatomy-to-Tract Mapping Infers White Matter Pathways Without Diffusion Streamline Propagation
Source: bioRxiv. 2025 Oct 21:2024.12.24.630267. Preprint. [Version 3] doi: 10.1101/2024.12.24.630267 (PMC12338524; doi:10.1101/2024.12.24.630267)
Supplement: Supplement 1 [file media-1.pdf]

Supplementary Note: Architectural Details of ATM

The segmentation module of ATM comprises a total of C 3D convolutional downsampling blocks, followed by $C - 1$ upsampling blocks. The entire module is complemented by a long-skip connection for precise bundle segmentation. These convolutional blocks employ a kernel size of 3, and each block is equipped with filters specified as {32, 64, 128, 256}, followed by upsampling blocks with filter sizes of {128, 64, 32}. The final upsampling block is followed by a 3D convolutional output layer with a filter size of 1. The encoder-decoder component of the streamline VAE module is structured with C 1D convolutional downsampling and upsampling blocks. This module utilizes a kernel size of 63, a latent space dimensionality of 64, and filter size for each downsampling and upsampling block are set as {32, 64, 128, 256} and {256, 128, 64, 32}, respectively. Each upsampling block is coupled with a FiLM layer[41] for adaptive feature modulation except the output layer with a filter size of 3. These FiLM layers receive input from the FiLM shared-weight block, which consists of two layers of multi-layer perceptron (MLP).

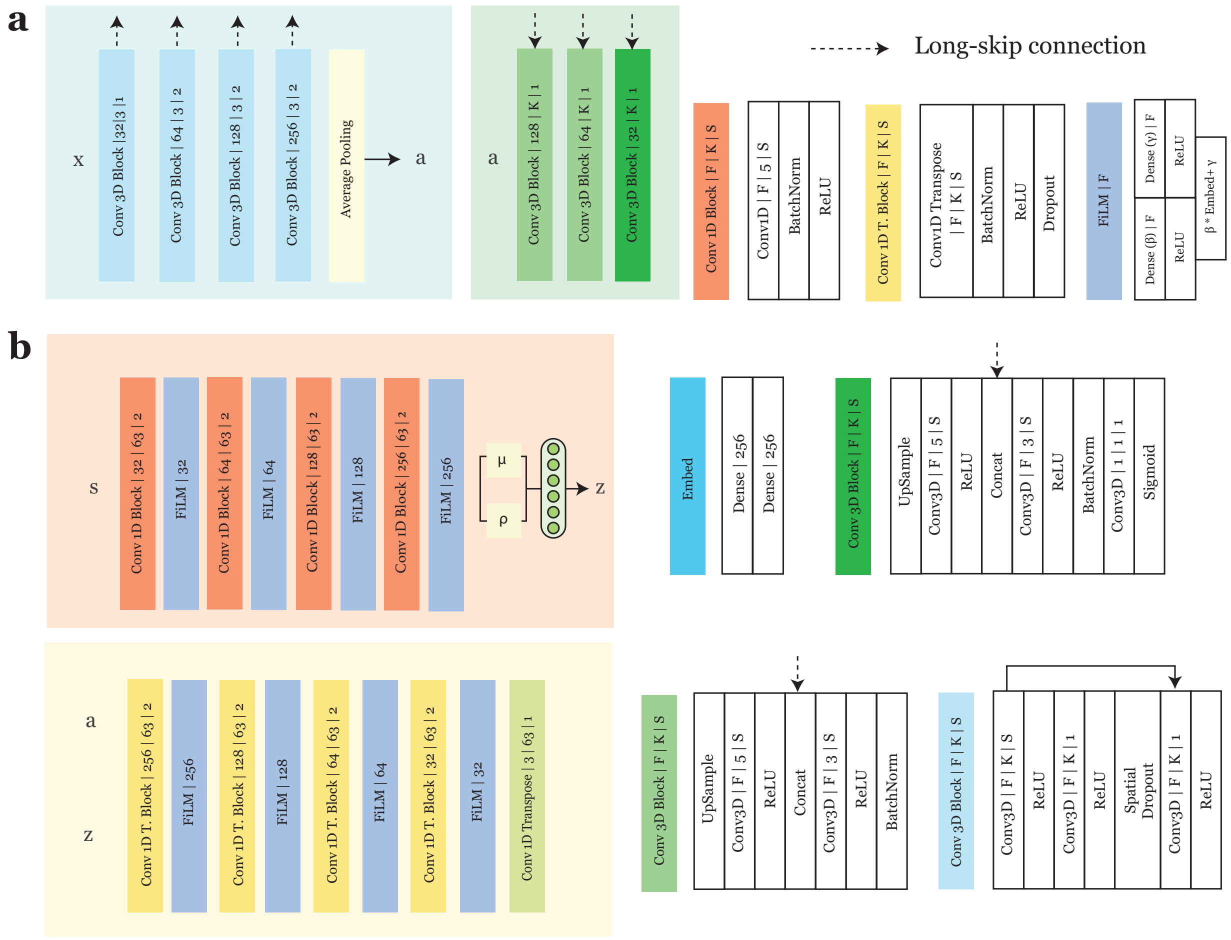

Fig. S1 | **ATM architectural details**. **a**, Network architecture of T1w encoder, E_A, and decoder, D_A. **b**, Network architectures of streamline encoder, E_S, and decoder, D_S.

Tab. S1 | White matter bundles.

**Bundle**	**Abbreviation**
Arcuate fascicle	AF
Corpus callosum, Frontal lobe (most anterior part)	CC_Fr_1
Corpus callosum, Frontal lobe (most anterior part)	CC_Fr_2
Corpus callosum, Occipital lobe	CC_Oc
Corpus callosum, Parietal lobe	CC_Pa
Corpus callosum, Pre/Post central gyri	CC_Pr_Po
Cingulum	CG
Frontal aslant tract	FAT
Fronto-pontine tract	FPT
Inferior fronto-occipital fasciculus	IFOF
Inferior longitudinal fasciculus	ILF
Middle cerebellar peduncle	MCP
Middle longitudinal fascicle	MdLF
Optic radiation and Meyer's loop	OR_ML
Parieto-occipito pontine tract	POPT
Pyramidal tract	PYT
Superior longitudinal fasciculus	SLF
Uncinate fasciculus	UF

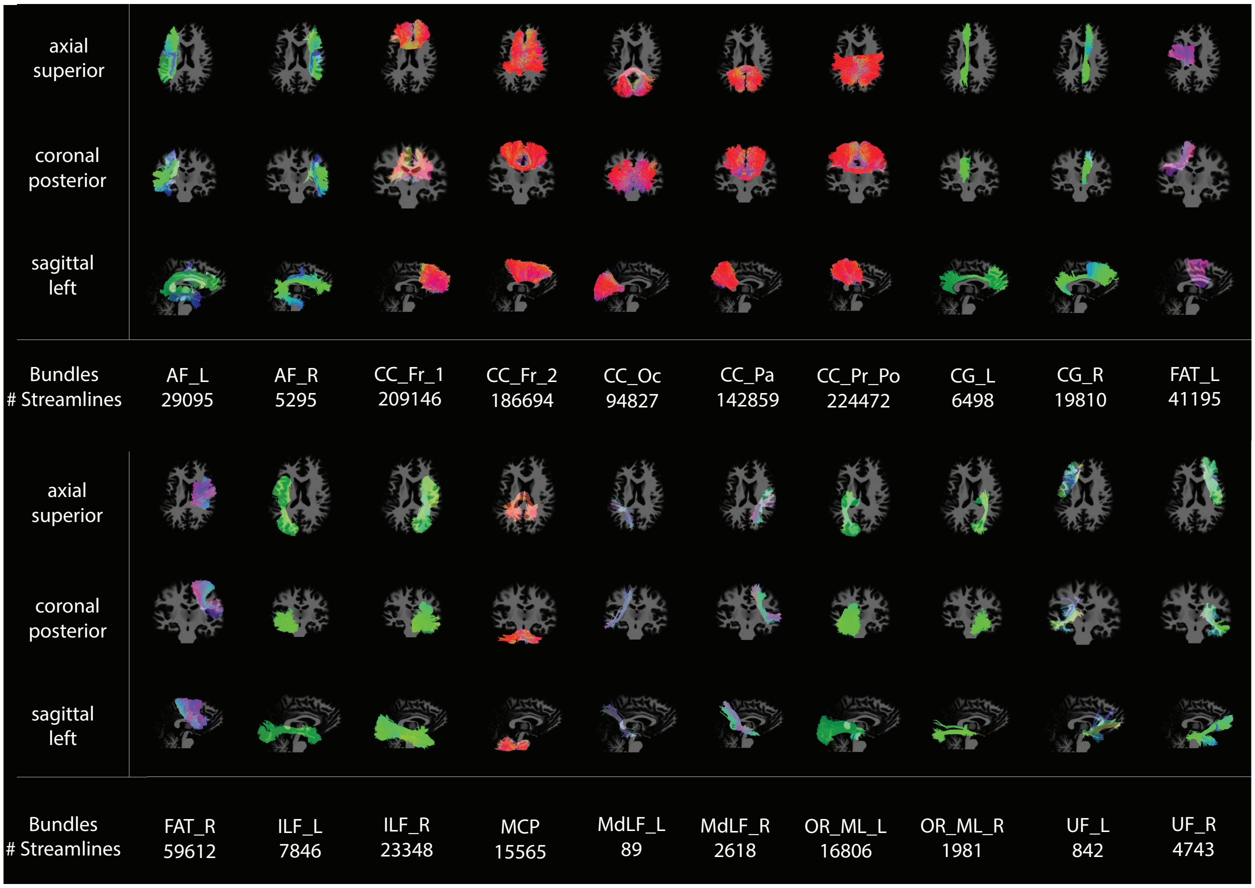

Fig. S2 | **Bundle comparison.** Ground truth bundles from Tractoinferno for a single representative subject.

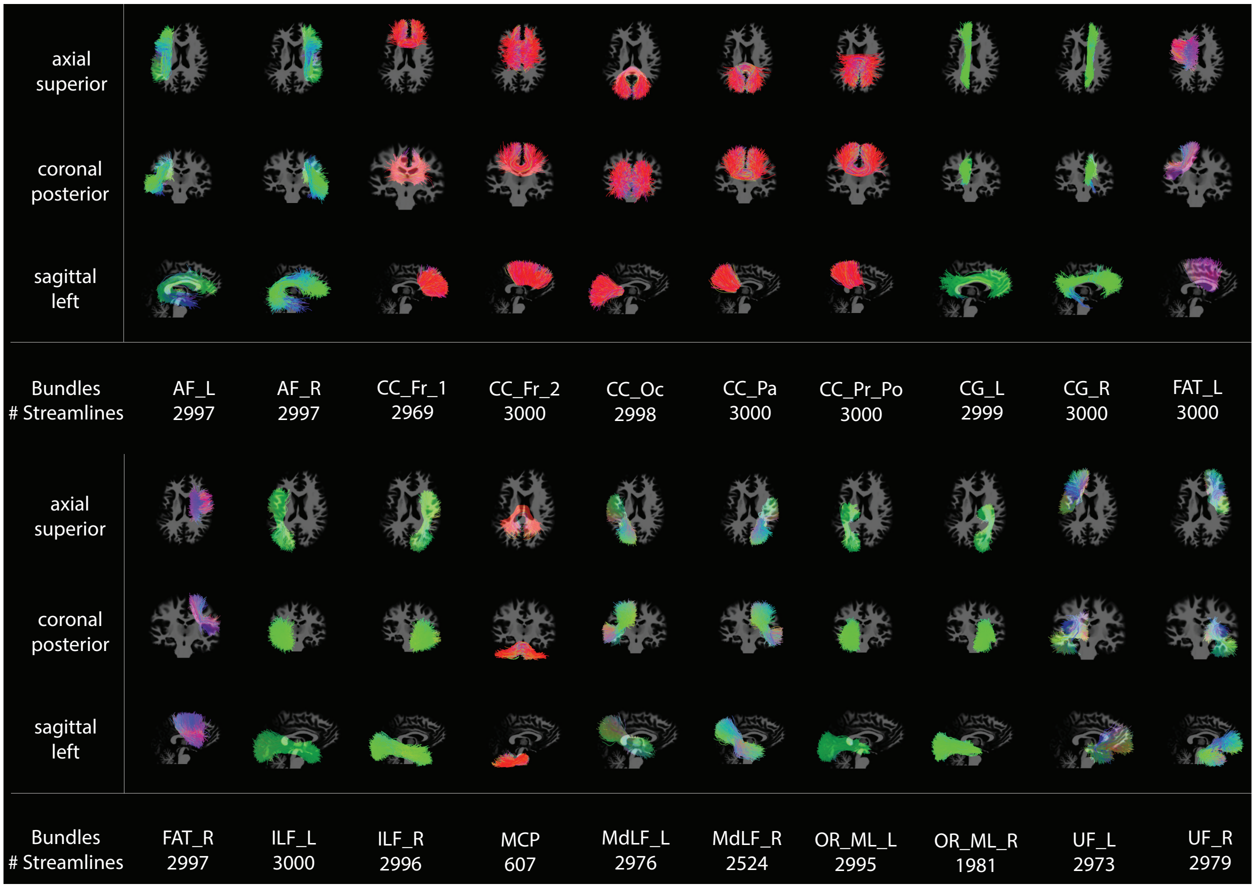

Fig. S3 | **Bundle comparison.** Bundles generated with ATM for a single representative subject.

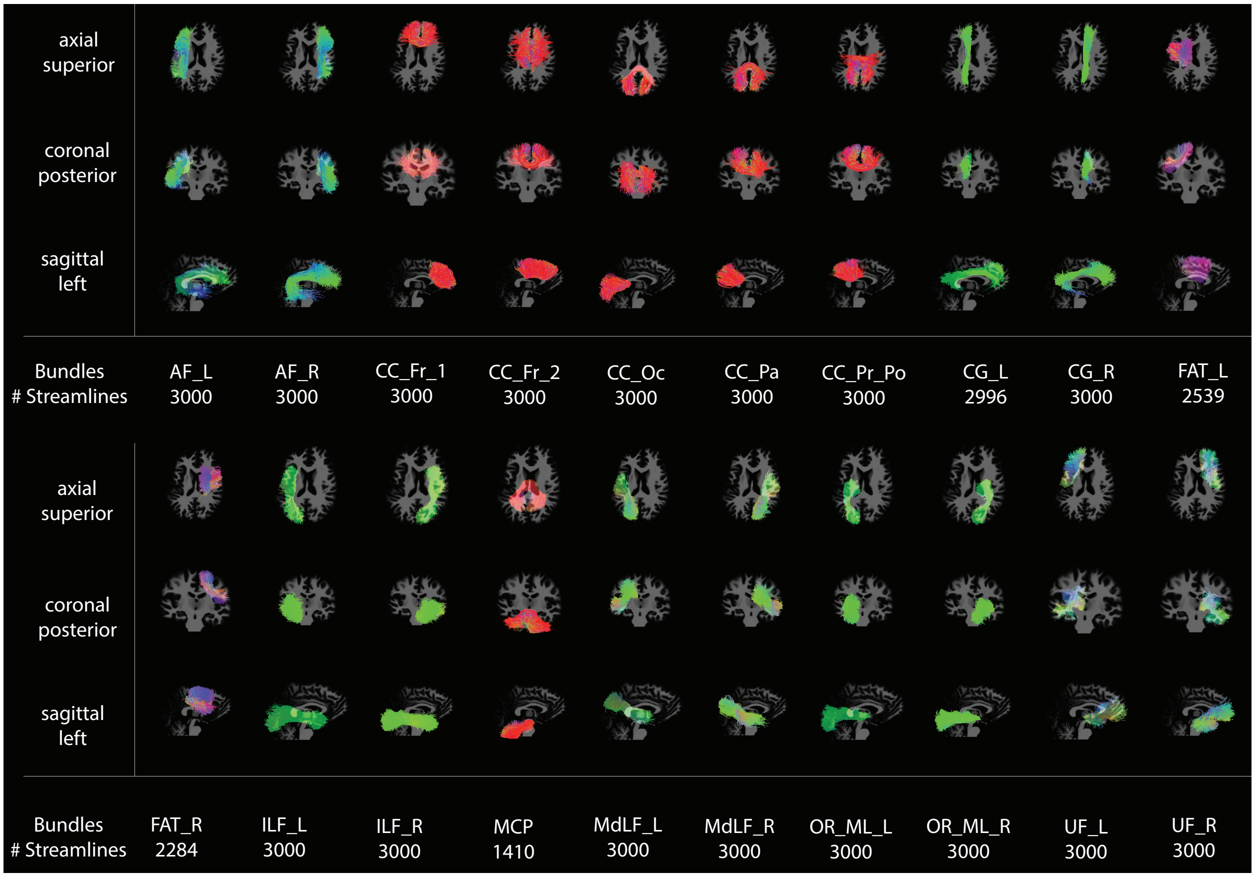

Fig. S4 | Bundle comparison. Bundles generated with ATM-Population for a single representative subject.

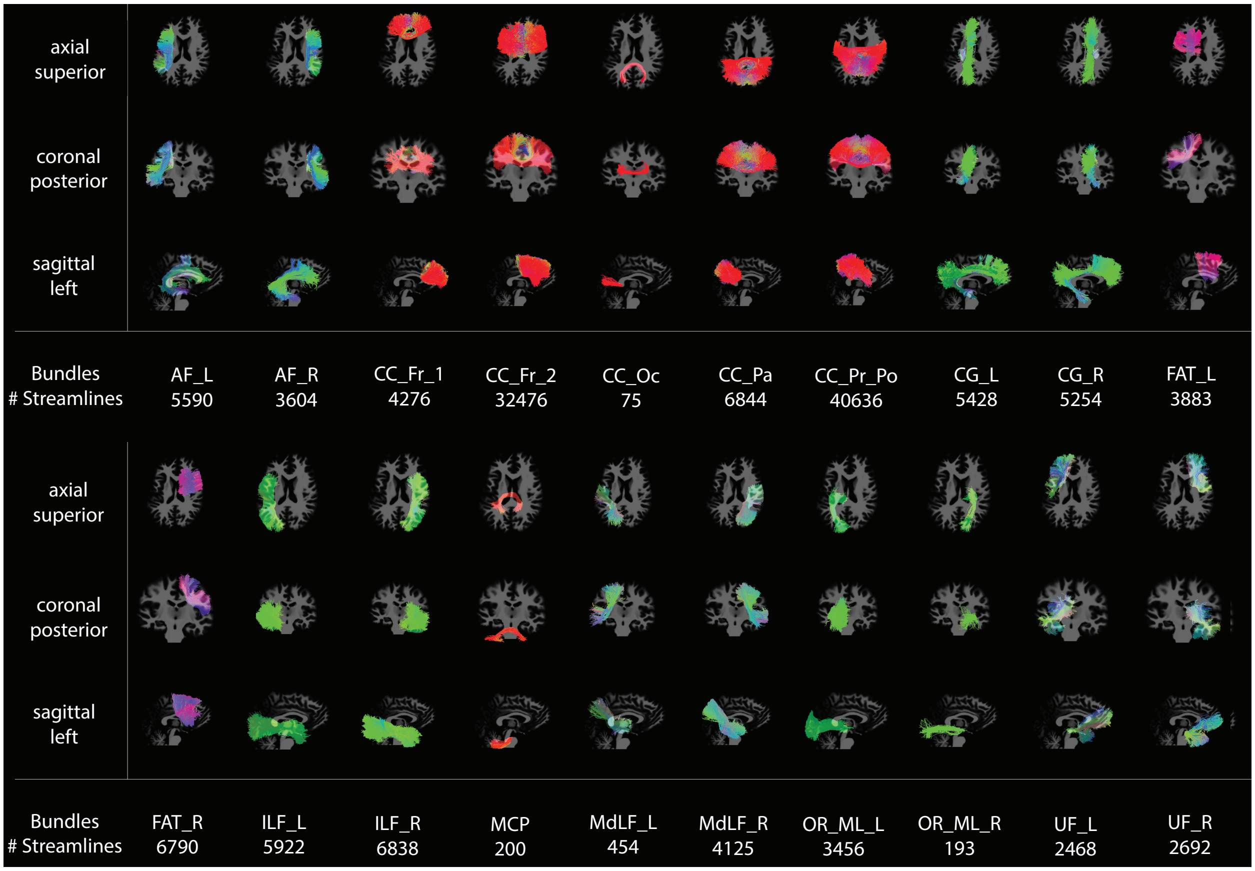

Fig. S5 | **Bundle comparison.** Bundles generated with MRtrix and BundleSeg for a single representative subject.

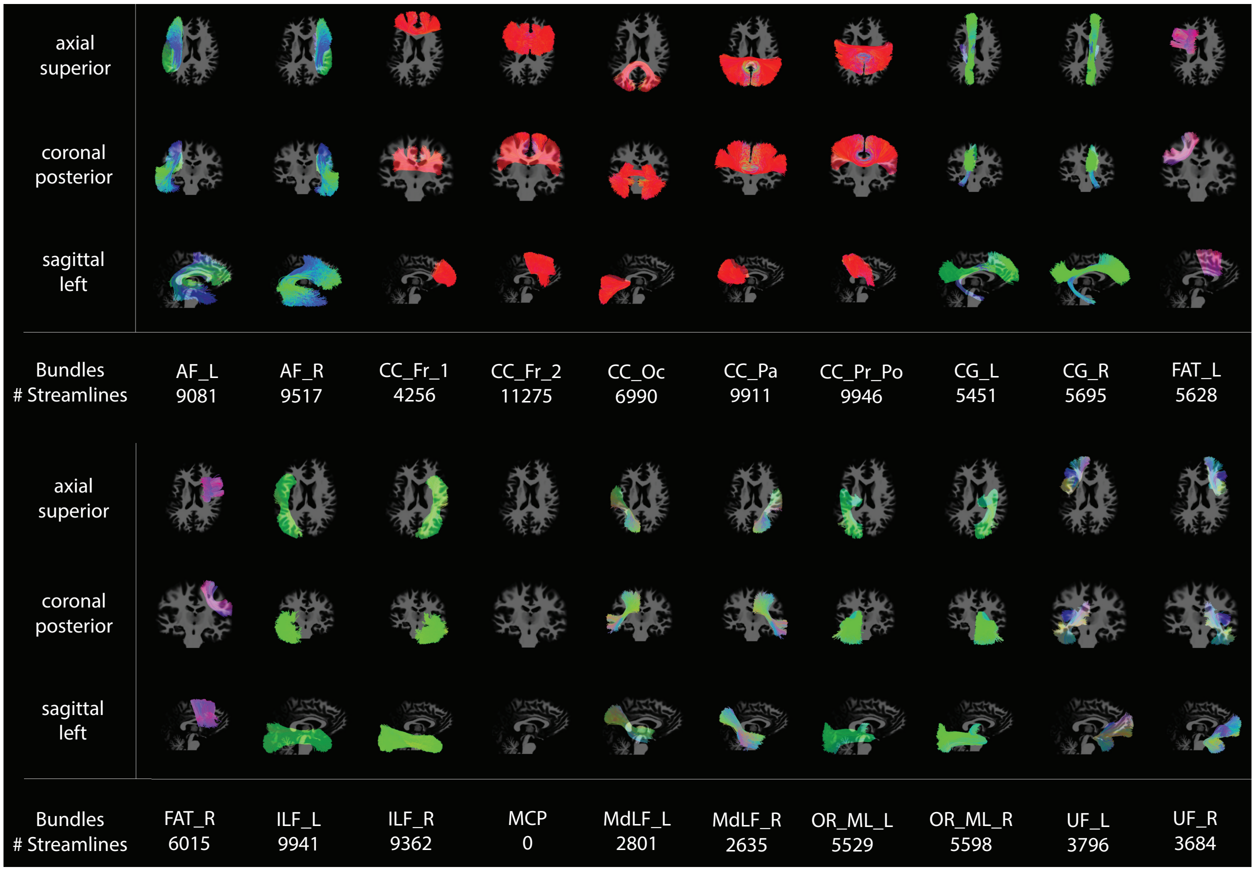

Fig. S6 | **Bundle comparison.** Bundles generated with SCIL WM atlas warping for a single representative subject.

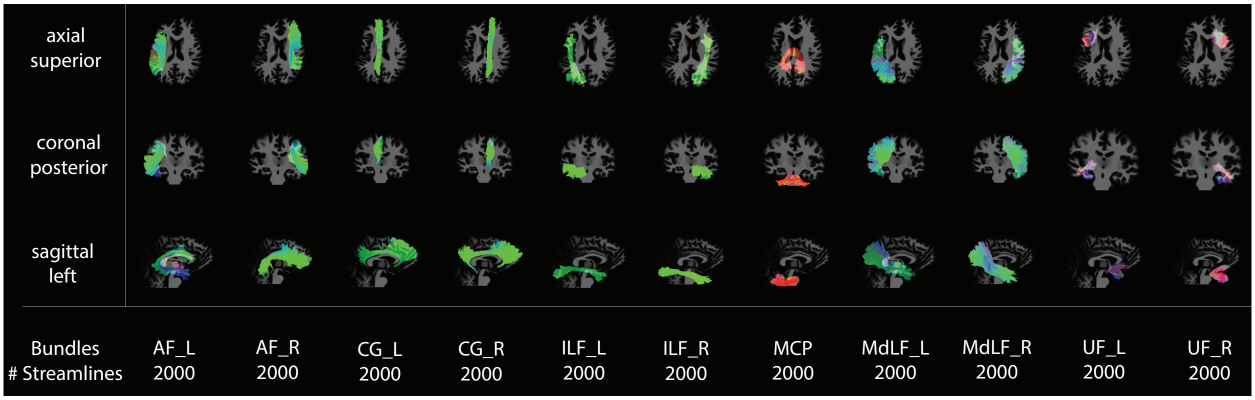

Fig. S7 | Bundle comparison. Bundles generated with TractSeg for a single representative subject.

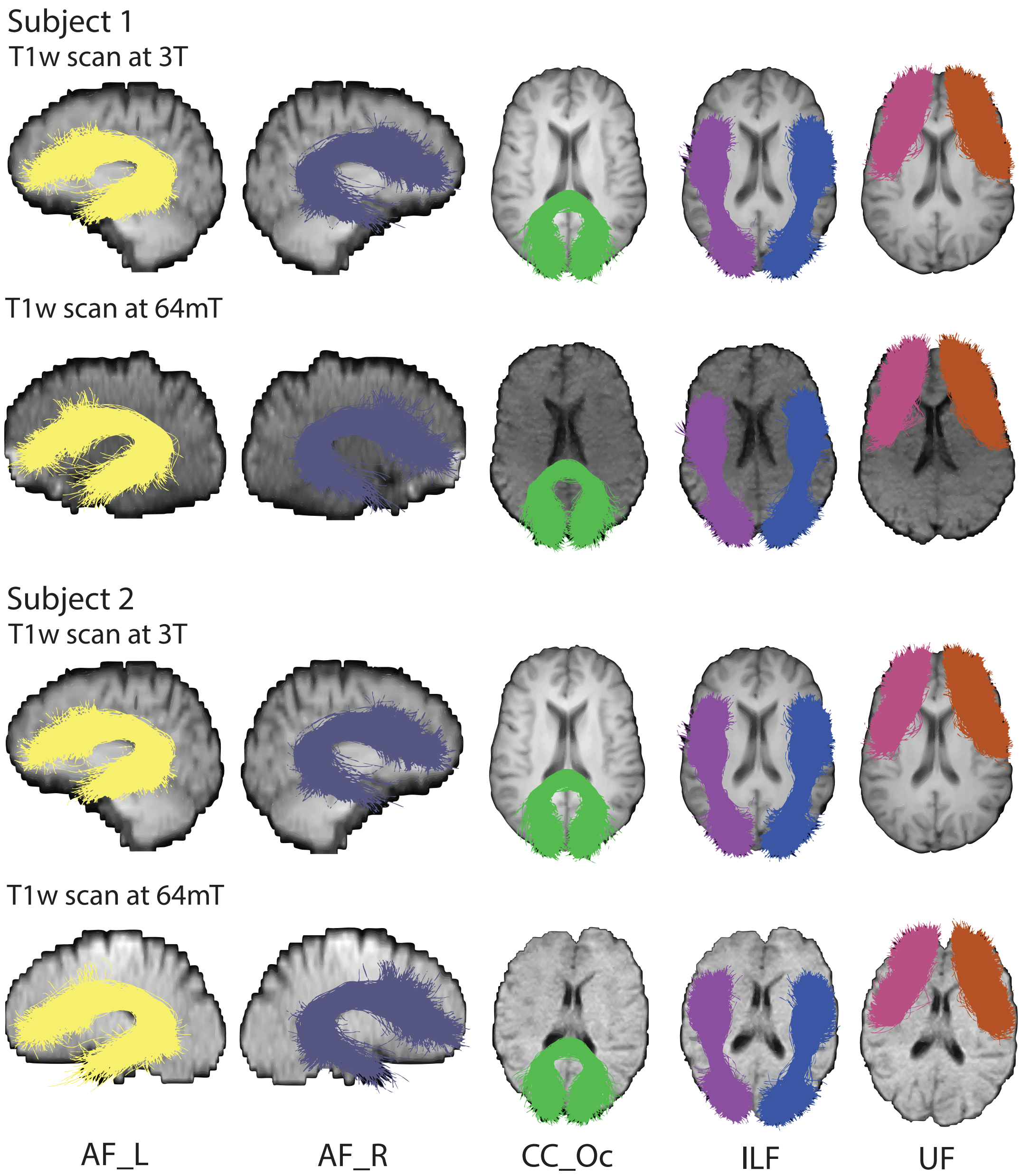

Fig. S8 | Clinical scans. Bundles generated with ATM for clincal T1w scans acquired at 3T with 1.5 mm × 1.5 mm × 5 mm resolution and at 64 mT with 1.6 mm × 1.6 mm × 5 mm resolution.

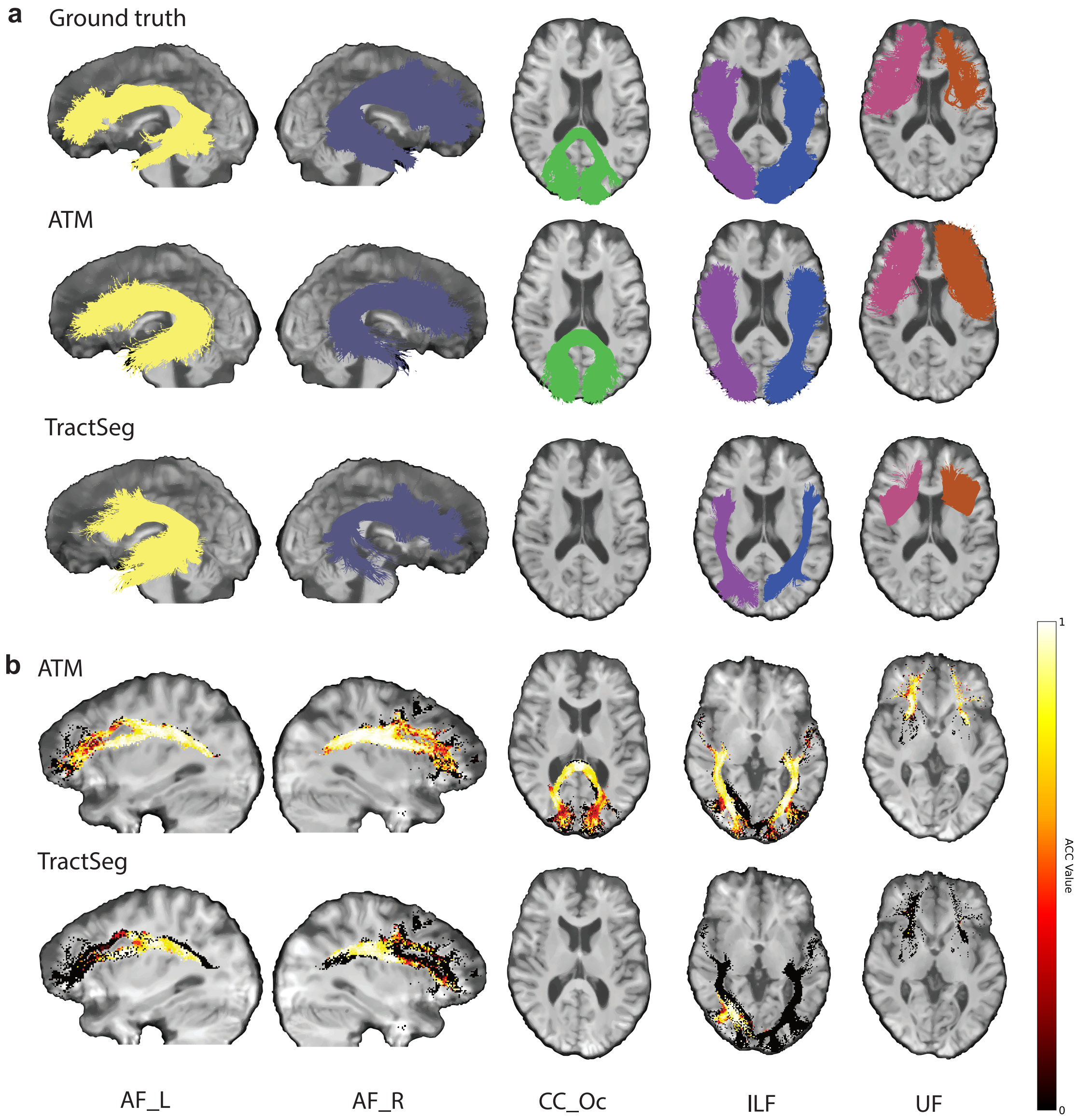

Fig. S9 | **Bundle comparison and orientation alignment.** **a**, Qualitative comparison of representative bundles reconstructed with ATM and TractSeg. **b**, Angular correlation coefficients (ACCs) computed between the tract orientation density maps of the ground truth and generated streamlines. TractSeg does not generate the CC_Oc bundle, as this bundle was not part of its training set. (TractoInferno Subject ID: 1135)

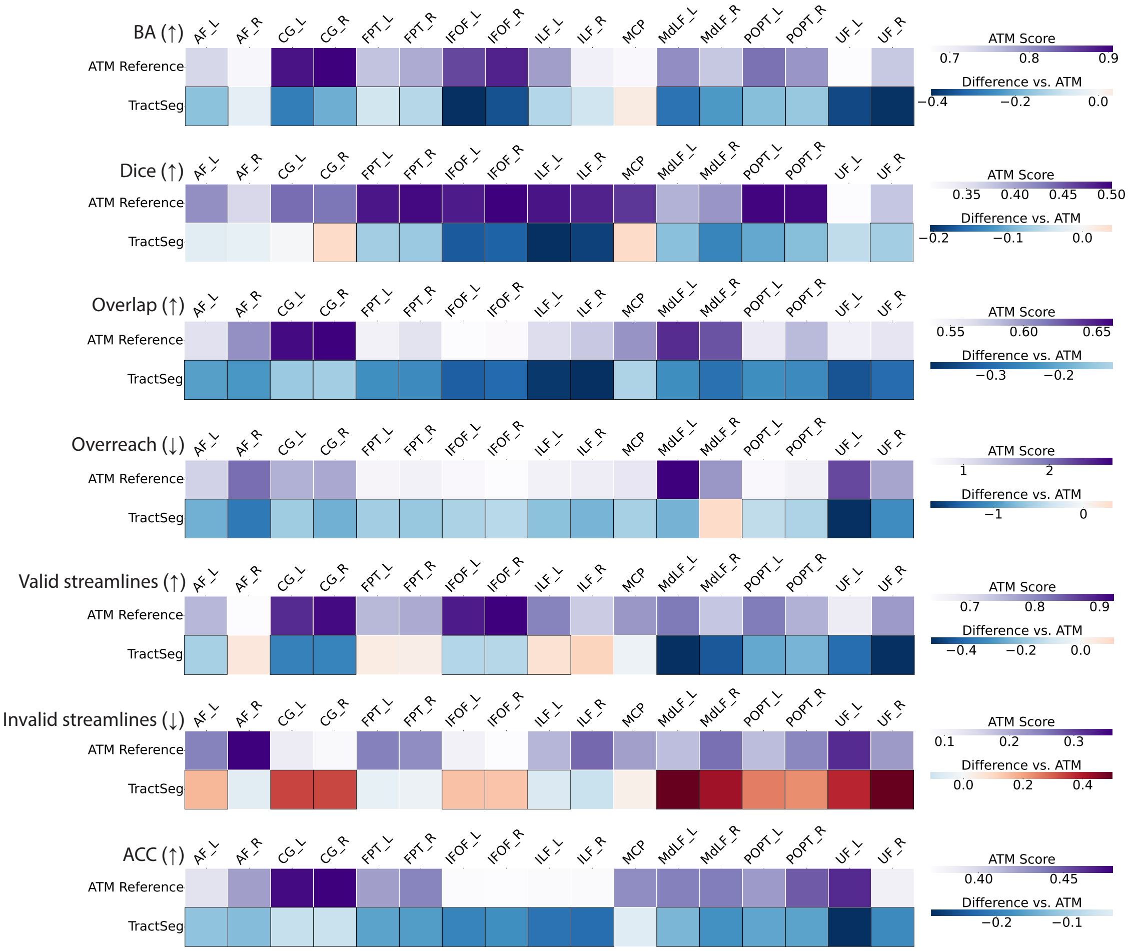

Fig. S10 | Similarity and coverage of bundles generated with ATM and TractSeg. The first row of each heatmap shows the ATM values as the baseline. The subsequent row displays the paired performance differences of TractSeg relative to this baseline. For each metric, the arrow indicates preference: ↑ higher is better and ↓ lower is better. Cells outlined with squares indicate statistically significant differences.

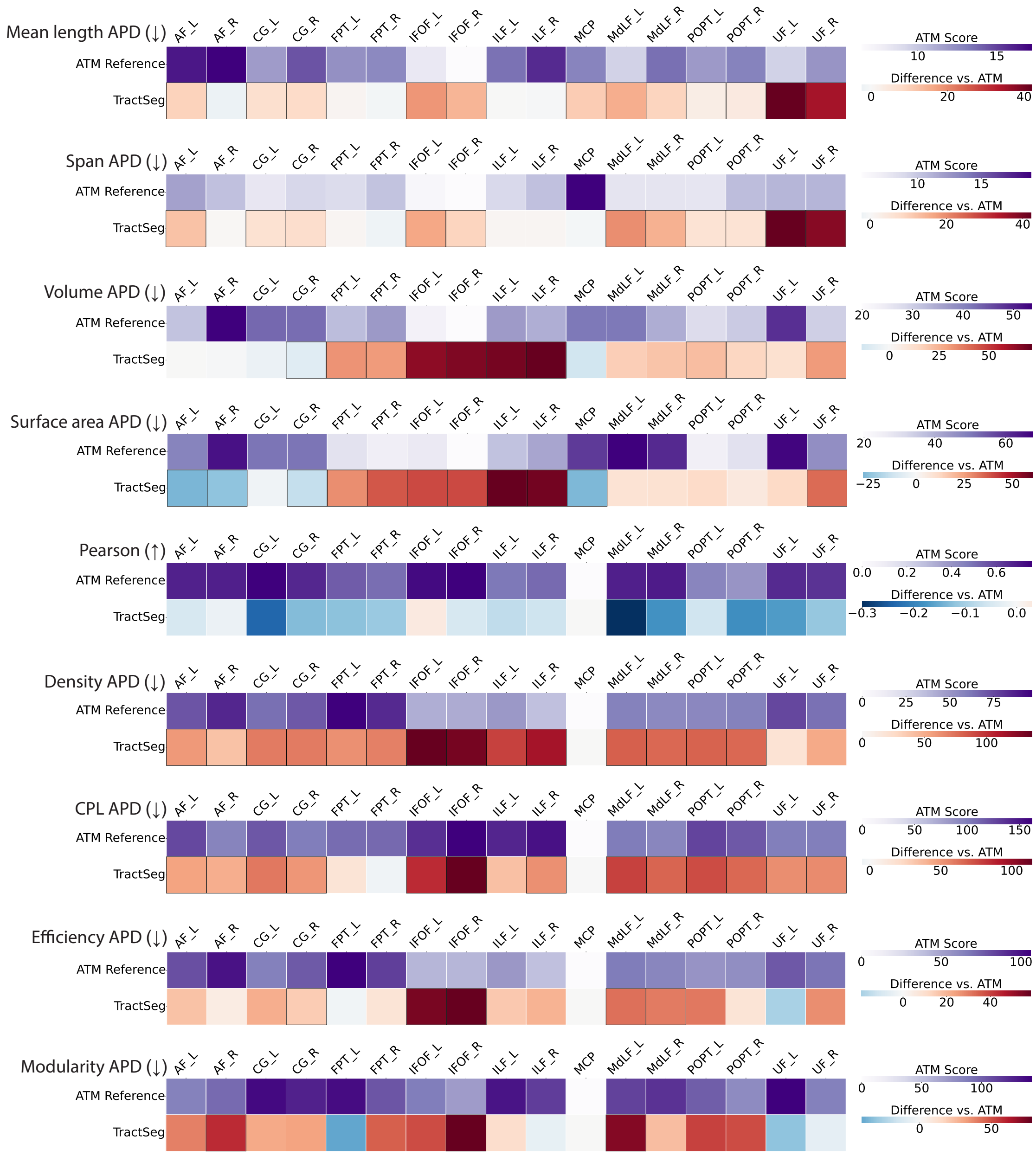

Fig. S11 | Geometry and connectomics of bundles generated with ATM and TractSeg. The first row of each heatmap shows the ATM values as the baseline. The subsequent row displays the paired performance differences of TractSeg relative to this baseline. For each metric, the arrow indicates preference: ↑ higher is better and ↓ lower is better. Cells outlined in squares indicate statistically significant differences.

Tab. S2 | Similarity, coverage, and validity of individual bundles generated with ATM. Values are presented as mean ± standard deviation. For each metric, the arrow indicates preference: ↑ higher is better, ↓ lower is better.

Bundles	BA (↑)	Coverage			Validity		ACC (↑)
		Dice (↑)	Overlap (↑)	Overreach (↓)	VS (↑)	IS (↓)	
AF_L	0.7365 ± 0.1000	0.4149 ± 0.0540	0.5621 ± 0.0817	1.2206 ± 0.6170	0.7524 ± 0.1310	0.2476 ± 0.1310	0.3934 ± 0.0665
AF_R	0.6876 ± 0.1392	0.3607 ± 0.0878	0.6036 ± 0.0939	2.0266 ± 1.2352	0.6403 ± 0.2039	0.3597 ± 0.2039	0.4239 ± 0.0667
CC_Fr_1	0.8637 ± 0.1135	0.5175 ± 0.0585	0.5203 ± 0.0983	0.4976 ± 0.2881	0.8855 ± 0.1199	0.1145 ± 0.1199	0.3742 ± 0.0819
CC_Fr_2	0.8498 ± 0.0653	0.4857 ± 0.0509	0.4369 ± 0.0965	0.3497 ± 0.2158	0.9158 ± 0.0717	0.0842 ± 0.0717	0.3418 ± 0.0737
CC_Oc	0.8516 ± 0.0663	0.5287 ± 0.0489	0.5758 ± 0.0635	0.6150 ± 0.2440	0.8278 ± 0.0885	0.1722 ± 0.0885	0.3932 ± 0.0442
CC_Pa	0.9420 ± 0.0178	0.5848 ± 0.0281	0.5633 ± 0.0830	0.3592 ± 0.1676	0.9567 ± 0.0216	0.0433 ± 0.0216	0.4269 ± 0.0658
CC_Pr_Po	0.9355 ± 0.0341	0.5560 ± 0.0428	0.4958 ± 0.0727	0.2805 ± 0.0942	0.9615 ± 0.0162	0.0385 ± 0.0162	0.3774 ± 0.0560
CG_L	0.8914 ± 0.0910	0.4382 ± 0.0831	0.6571 ± 0.0639	1.5003 ± 0.8744	0.8824 ± 0.1431	0.1176 ± 0.1431	0.4751 ± 0.0543
CG_R	0.9055 ± 0.0552	0.4331 ± 0.0765	0.6610 ± 0.0592	1.5706 ± 1.0833	0.9123 ± 0.0717	0.0877 ± 0.0717	0.4788 ± 0.0645
FAT_L	0.8702 ± 0.0627	0.5504 ± 0.0448	0.5753 ± 0.0711	0.5303 ± 0.2840	0.8826 ± 0.0815	0.1174 ± 0.0815	0.4272 ± 0.0533
FAT_R	0.8480 ± 0.0516	0.5239 ± 0.0432	0.5634 ± 0.0750	0.5941 ± 0.2330	0.8675 ± 0.0587	0.1325 ± 0.0587	0.4184 ± 0.0599
FPT_L	0.7563 ± 0.1323	0.4870 ± 0.0659	0.5472 ± 0.0730	0.7568 ± 0.4906	0.7511 ± 0.1355	0.2489 ± 0.1355	0.4240 ± 0.0639
FPT_R	0.7769 ± 0.1017	0.4946 ± 0.0761	0.5611 ± 0.0575	0.8062 ± 0.6539	0.7650 ± 0.1150	0.2350 ± 0.1150	0.4354 ± 0.0442
IFOF_L	0.8548 ± 0.0552	0.4832 ± 0.0316	0.5364 ± 0.0530	0.6971 ± 0.2514	0.8963 ± 0.0486	0.1037 ± 0.0486	0.3730 ± 0.0422
IFOF_R	0.8811 ± 0.0411	0.5004 ± 0.0236	0.5386 ± 0.0454	0.6181 ± 0.1696	0.9217 ± 0.0406	0.0783 ± 0.0406	0.3720 ± 0.0347
ILF_L	0.7876 ± 0.1054	0.4885 ± 0.0686	0.5646 ± 0.0707	0.8207 ± 0.5547	0.8086 ± 0.1227	0.1914 ± 0.1227	0.3742 ± 0.0519
ILF_R	0.7006 ± 0.2435	0.4799 ± 0.0684	0.5771 ± 0.0882	0.8951 ± 0.5654	0.7285 ± 0.2502	0.2715 ± 0.2502	0.3748 ± 0.0689
MCP	0.6850 ± 0.1771	0.4674 ± 0.0662	0.6025 ± 0.1354	0.9755 ± 0.3588	0.7861 ± 0.1311	0.2139 ± 0.1311	0.4316 ± 0.1100
MdLF_L	0.8025 ± 0.1923	0.3904 ± 0.1373	0.6433 ± 0.1247	2.7284 ± 4.9993	0.8178 ± 0.2170	0.1822 ± 0.2170	0.4371 ± 0.0945
MdLF_R	0.7530 ± 0.2253	0.4104 ± 0.1407	0.6292 ± 0.1647	1.7125 ± 1.1728	0.7355 ± 0.2440	0.2645 ± 0.2440	0.4381 ± 0.1223
OR_ML_L	0.9234 ± 0.0521	0.4670 ± 0.0689	0.6785 ± 0.0812	1.3203 ± 0.6718	0.9218 ± 0.0610	0.0782 ± 0.0610	0.4705 ± 0.0671
OR_ML_R	0.8520 ± 0.1234	0.3730 ± 0.1016	0.6928 ± 0.1032	2.2754 ± 1.1133	0.8479 ± 0.1626	0.1521 ± 0.1626	0.4754 ± 0.0725
POPT_L	0.8267 ± 0.0906	0.4973 ± 0.0628	0.5550 ± 0.0760	0.6965 ± 0.2918	0.8161 ± 0.0938	0.1839 ± 0.0938	0.4256 ± 0.0652
POPT_R	0.7955 ± 0.0784	0.4955 ± 0.0762	0.5842 ± 0.0783	0.8341 ± 0.5008	0.7581 ± 0.1239	0.2419 ± 0.1239	0.4496 ± 0.0664
PYT_L	0.8664 ± 0.0944	0.5673 ± 0.0345	0.5306 ± 0.0577	0.3379 ± 0.1038	0.8817 ± 0.0573	0.1183 ± 0.0573	0.4097 ± 0.0488
PYT_R	0.8661 ± 0.0857	0.5698 ± 0.0342	0.5360 ± 0.0446	0.3459 ± 0.0931	0.8913 ± 0.0514	0.1087 ± 0.0514	0.4169 ± 0.0405
SLF_L	0.5883 ± 0.1786	0.3268 ± 0.1011	0.5707 ± 0.1311	2.1464 ± 1.0886	0.5322 ± 0.1935	0.4678 ± 0.1935	0.3934 ± 0.0924
SLF_R	0.6989 ± 0.1487	0.4024 ± 0.1100	0.6033 ± 0.0863	1.6959 ± 1.1546	0.6568 ± 0.2123	0.3432 ± 0.2123	0.4090 ± 0.0634
UF_L	0.6763 ± 0.2320	0.3121 ± 0.1300	0.5507 ± 0.1630	2.2471 ± 1.1113	0.6790 ± 0.2396	0.3210 ± 0.2396	0.4644 ± 0.0975
UF_R	0.7524 ± 0.1792	0.3761 ± 0.1096	0.5588 ± 0.1258	1.5944 ± 0.9047	0.7814 ± 0.1933	0.2186 ± 0.1933	0.3827 ± 0.1084

Tab. S3 | Similarity, coverage, and validity of population bundles generated with ATM. Values are presented as mean ± standard deviation. For each metric, the arrow indicates preference: ↑ higher is better, ↓ lower is better.

Bundles	BA (↑)	Dice (↑)	Coverage Overlap (↑)	Overreach (↓)	Validity VS (↑)	IS (↓)	ACC (↑)
AF_L	0.4972 ± 0.2037	0.3491 ± 0.0681	0.4503 ± 0.0791	1.2223 ± 0.6353	0.5695 ± 0.2216	0.4305 ± 0.2216	0.2573 ± 0.0555
AF_R	0.5316 ± 0.1828	0.3058 ± 0.0862	0.5436 ± 0.1026	2.4976 ± 1.7982	0.5490 ± 0.2407	0.4510 ± 0.2407	0.3118 ± 0.0679
CC_Fr_1	0.7402 ± 0.2111	0.4339 ± 0.1301	0.4257 ± 0.1648	0.4769 ± 0.3904	0.7461 ± 0.2570	0.2539 ± 0.2570	0.2626 ± 0.1043
CC_Fr_2	0.7878 ± 0.1797	0.4118 ± 0.1323	0.3708 ± 0.1521	0.3579 ± 0.2682	0.8793 ± 0.1360	0.1207 ± 0.1360	0.2387 ± 0.0986
CC_Oc	0.5739 ± 0.2737	0.4081 ± 0.0733	0.3907 ± 0.0903	0.5208 ± 0.2446	0.7162 ± 0.2214	0.2838 ± 0.2214	0.2116 ± 0.0553
CC_Pa	0.8306 ± 0.0778	0.4741 ± 0.0512	0.4119 ± 0.0744	0.3178 ± 0.1560	0.9318 ± 0.0658	0.0682 ± 0.0658	0.2627 ± 0.0552
CC_Pr_Po	0.8171 ± 0.1095	0.4509 ± 0.0613	0.3676 ± 0.0730	0.2535 ± 0.1144	0.9162 ± 0.0801	0.0838 ± 0.0801	0.2372 ± 0.0476
CG_L	0.7453 ± 0.2159	0.3804 ± 0.0741	0.5076 ± 0.0799	1.2862 ± 0.7755	0.7597 ± 0.2700	0.2403 ± 0.2700	0.3070 ± 0.0587
CG_R	0.7849 ± 0.1907	0.3860 ± 0.0793	0.5479 ± 0.1191	1.3903 ± 0.8518	0.8092 ± 0.1876	0.1908 ± 0.1876	0.3343 ± 0.0999
FAT_L	0.6981 ± 0.1695	0.4439 ± 0.1202	0.4178 ± 0.1325	0.4142 ± 0.2182	0.8013 ± 0.1198	0.1987 ± 0.1198	0.2543 ± 0.0833
FAT_R	0.7079 ± 0.1700	0.4289 ± 0.1155	0.4306 ± 0.1525	0.5111 ± 0.2746	0.8148 ± 0.1224	0.1852 ± 0.1224	0.2626 ± 0.1002
FPT_L	0.5383 ± 0.1815	0.4203 ± 0.0658	0.4774 ± 0.0762	0.8403 ± 0.4379	0.6112 ± 0.1760	0.3888 ± 0.1760	0.3066 ± 0.0595
FPT_R	0.5186 ± 0.1978	0.4226 ± 0.0708	0.4834 ± 0.0625	0.8942 ± 0.6174	0.5802 ± 0.2092	0.4198 ± 0.2092	0.3120 ± 0.0520
IFOF_L	0.5334 ± 0.2346	0.3883 ± 0.0431	0.3914 ± 0.0560	0.6302 ± 0.2024	0.7586 ± 0.1817	0.2414 ± 0.1817	0.2276 ± 0.0453
IFOF_R	0.6637 ± 0.1795	0.4203 ± 0.0222	0.4422 ± 0.0447	0.6623 ± 0.1456	0.8098 ± 0.1325	0.1902 ± 0.1325	0.2593 ± 0.0278
ILF_L	0.4647 ± 0.2355	0.3793 ± 0.0671	0.3917 ± 0.0619	0.7321 ± 0.4640	0.6535 ± 0.2471	0.3465 ± 0.2471	0.2099 ± 0.0479
ILF_R	0.5136 ± 0.1872	0.3859 ± 0.0478	0.4051 ± 0.0534	0.7195 ± 0.3312	0.6863 ± 0.1998	0.3137 ± 0.1998	0.2212 ± 0.0353
MCP	0.2257 ± 0.2003	0.2593 ± 0.0900	0.3470 ± 0.1217	1.3768 ± 0.5541	0.2722 ± 0.2075	0.7278 ± 0.2075	0.1946 ± 0.0895
MdLF_L	0.5507 ± 0.2734	0.3272 ± 0.1194	0.4919 ± 0.1000	2.3893 ± 2.9293	0.6272 ± 0.3094	0.3728 ± 0.3094	0.2748 ± 0.0699
MdLF_R	0.5814 ± 0.2460	0.3358 ± 0.1260	0.5265 ± 0.1133	3.1412 ± 5.0060	0.6132 ± 0.2908	0.3868 ± 0.2908	0.3050 ± 0.0732
OR_ML_L	0.7133 ± 0.1918	0.3732 ± 0.0820	0.4711 ± 0.1067	1.1130 ± 0.5520	0.8134 ± 0.1872	0.1866 ± 0.1872	0.2679 ± 0.0742
OR_ML_R	0.6056 ± 0.2224	0.2723 ± 0.0804	0.4588 ± 0.1184	2.4736 ± 2.4420	0.6605 ± 0.2243	0.3395 ± 0.2243	0.2674 ± 0.0876
POPT_L	0.5037 ± 0.1883	0.3901 ± 0.1069	0.4100 ± 0.1145	0.6741 ± 0.3649	0.6473 ± 0.2115	0.3527 ± 0.2115	0.2683 ± 0.0804
POPT_R	0.4539 ± 0.2014	0.3854 ± 0.1132	0.4226 ± 0.1190	0.7927 ± 0.5398	0.5876 ± 0.2328	0.4124 ± 0.2328	0.2756 ± 0.0815
PYT_L	0.5486 ± 0.1989	0.4521 ± 0.1007	0.4086 ± 0.1027	0.3651 ± 0.1428	0.7075 ± 0.2088	0.2925 ± 0.2088	0.2664 ± 0.0701
PYT_R	0.6477 ± 0.2030	0.4927 ± 0.0491	0.4653 ± 0.0682	0.4194 ± 0.1368	0.8091 ± 0.1299	0.1909 ± 0.1299	0.3091 ± 0.0455
SLF_L	0.4941 ± 0.2052	0.2696 ± 0.1062	0.5140 ± 0.1481	3.0548 ± 3.0817	0.5111 ± 0.2381	0.4889 ± 0.2381	0.2891 ± 0.0856
SLF_R	0.5760 ± 0.2273	0.3378 ± 0.1159	0.4722 ± 0.1280	1.7756 ± 1.7986	0.6202 ± 0.2685	0.3798 ± 0.2685	0.2680 ± 0.0705
UF_L	0.3520 ± 0.1894	0.2374 ± 0.1007	0.4278 ± 0.0899	3.0130 ± 2.2913	0.4130 ± 0.2245	0.5870 ± 0.2245	0.2277 ± 0.0527
UF_R	0.4706 ± 0.1851	0.2848 ± 0.0984	0.4681 ± 0.0800	2.6749 ± 3.0466	0.5408 ± 0.1865	0.4592 ± 0.1865	0.2595 ± 0.0524

Tab. S4 | Similarity, coverage, and validity of bundles generated from MRtrix with BundleSeg. Values are presented as mean ± standard deviation. For each metric, the arrow indicates preference: ↑ higher is better, ↓ lower is better.

Bundles	BA (↑)	Coverage			Validity		ACC (↑)
		Dice (↑)	Overlap (↑)	Overreach (↓)	VS (↑)	IS (↓)	
AF_L	0.3683 ± 0.1650	0.4492 ± 0.0846	0.7046 ± 0.1479	1.6085 ± 1.1178	0.5412 ± 0.2079	0.4588 ± 0.2079	0.5826 ± 0.1174
AF_R	0.3163 ± 0.1808	0.3656 ± 0.1204	0.7238 ± 0.1652	2.6595 ± 1.8034	0.4552 ± 0.1903	0.5448 ± 0.1903	0.5996 ± 0.1350
CC_Fr_1	0.8905 ± 0.0640	0.5636 ± 0.1282	0.7193 ± 0.1915	0.8672 ± 0.7374	0.9125 ± 0.0768	0.0875 ± 0.0768	0.6034 ± 0.1558
CC_Fr_2	0.8175 ± 0.1572	0.5399 ± 0.1343	0.7068 ± 0.1587	1.0329 ± 0.7882	0.6894 ± 0.3198	0.3106 ± 0.3198	0.6098 ± 0.1351
CC_Oc	0.3968 ± 0.1495	0.3944 ± 0.1252	0.2930 ± 0.1160	0.1356 ± 0.1137	0.7922 ± 0.1725	0.2078 ± 0.1725	0.2634 ± 0.1008
CC_Pa	0.7782 ± 0.0762	0.6095 ± 0.0585	0.7110 ± 0.1243	0.6534 ± 0.4556	0.8724 ± 0.0833	0.1276 ± 0.0833	0.6111 ± 0.0982
CC_Pr_Po	0.7370 ± 0.0973	0.5371 ± 0.0734	0.7707 ± 0.1131	1.1416 ± 0.4426	0.7432 ± 0.3368	0.2568 ± 0.3368	0.6660 ± 0.1010
CG_L	0.6219 ± 0.1240	0.3017 ± 0.0834	0.8114 ± 0.1543	4.1982 ± 2.3738	0.5804 ± 0.1379	0.4196 ± 0.1379	0.6573 ± 0.1217
CG_R	0.6737 ± 0.1189	0.2928 ± 0.0747	0.8484 ± 0.1875	4.7850 ± 3.5378	0.5914 ± 0.1594	0.4086 ± 0.1594	0.6842 ± 0.1489
FAT_L	0.8459 ± 0.0933	0.5357 ± 0.1352	0.7262 ± 0.1840	0.9707 ± 0.5377	0.8286 ± 0.1536	0.1714 ± 0.1536	0.6109 ± 0.1535
FAT_R	0.8614 ± 0.0703	0.5378 ± 0.1085	0.7672 ± 0.1668	1.1163 ± 0.5926	0.8397 ± 0.1240	0.1603 ± 0.1240	0.6436 ± 0.1404
FPT_L	0.5240 ± 0.1923	0.5301 ± 0.1020	0.5573 ± 0.1345	0.5892 ± 0.4804	0.7561 ± 0.1977	0.2439 ± 0.1977	0.4853 ± 0.1165
FPT_R	0.5802 ± 0.1641	0.5528 ± 0.1000	0.6328 ± 0.1372	0.7238 ± 0.6504	0.8074 ± 0.1424	0.1926 ± 0.1424	0.5499 ± 0.1155
IFOF_L	0.3206 ± 0.1565	0.4141 ± 0.0414	0.3397 ± 0.0817	0.2832 ± 0.1592	0.7336 ± 0.2674	0.2664 ± 0.2674	0.2949 ± 0.0636
IFOF_R	0.3830 ± 0.1789	0.4403 ± 0.0476	0.3791 ± 0.0815	0.3304 ± 0.1778	0.7650 ± 0.2371	0.2350 ± 0.2371	0.3260 ± 0.0657
ILF_L	0.6713 ± 0.1454	0.5047 ± 0.1002	0.8328 ± 0.1312	1.6741 ± 1.1211	0.7781 ± 0.1425	0.2219 ± 0.1425	0.6674 ± 0.0964
ILF_R	0.6777 ± 0.1254	0.4990 ± 0.0945	0.8614 ± 0.1290	1.7669 ± 0.9854	0.7398 ± 0.1306	0.2602 ± 0.1306	0.6902 ± 0.0943
MCP	0.5711 ± 0.3029	0.5121 ± 0.1283	0.5946 ± 0.1861	0.6924 ± 0.4555	0.8071 ± 0.1948	0.1929 ± 0.1948	0.5006 ± 0.1588
MdLF_L	0.6611 ± 0.2661	0.4130 ± 0.1593	0.7546 ± 0.1549	3.0138 ± 3.8191	0.6827 ± 0.2972	0.3173 ± 0.2972	0.6176 ± 0.1275
MdLF_R	0.7477 ± 0.1913	0.4072 ± 0.1748	0.8202 ± 0.1388	4.9458 ± 7.3139	0.7009 ± 0.2969	0.2991 ± 0.2969	0.6708 ± 0.1112
OR_ML_L	0.9042 ± 0.0721	0.5308 ± 0.0847	0.7879 ± 0.0908	1.2713 ± 0.6414	0.9267 ± 0.0670	0.0733 ± 0.0670	0.6592 ± 0.0698
OR_ML_R	0.8524 ± 0.1013	0.4447 ± 0.1344	0.8207 ± 0.1111	2.6992 ± 3.0106	0.8444 ± 0.1495	0.1556 ± 0.1495	0.6936 ± 0.0909
POPT_L	0.5587 ± 0.1489	0.5202 ± 0.0501	0.5473 ± 0.1021	0.5738 ± 0.3610	0.7563 ± 0.1735	0.2437 ± 0.1735	0.4826 ± 0.0874
POPT_R	0.5262 ± 0.1475	0.5127 ± 0.0628	0.5632 ± 0.1006	0.7098 ± 0.6573	0.6628 ± 0.2163	0.3372 ± 0.2163	0.4978 ± 0.0863
PYT_L	0.7241 ± 0.1753	0.6260 ± 0.0478	0.6521 ± 0.1193	0.4202 ± 0.1951	0.9008 ± 0.1160	0.0992 ± 0.1160	0.5729 ± 0.1020
PYT_R	0.6987 ± 0.1566	0.6345 ± 0.0359	0.6645 ± 0.1067	0.4277 ± 0.2162	0.8891 ± 0.0832	0.1109 ± 0.0832	0.5845 ± 0.0912
SLF_L	0.4285 ± 0.2163	0.3149 ± 0.1284	0.7706 ± 0.2017	4.2206 ± 4.6380	0.4369 ± 0.2183	0.5631 ± 0.2183	0.6396 ± 0.1649
SLF_R	0.5438 ± 0.2388	0.3932 ± 0.1662	0.7881 ± 0.2052	3.0247 ± 3.0350	0.5999 ± 0.2983	0.4001 ± 0.2983	0.6472 ± 0.1661
UF_L	0.6769 ± 0.2283	0.3499 ± 0.1699	0.7080 ± 0.2067	3.5978 ± 3.6483	0.6795 ± 0.2525	0.3205 ± 0.2525	0.5641 ± 0.1712
UF_R	0.7499 ± 0.1695	0.3942 ± 0.1262	0.7307 ± 0.1888	2.6260 ± 2.5430	0.7690 ± 0.1871	0.2310 ± 0.1871	0.5765 ± 0.1529

Tab. S5 | Similarity, coverage, and validity of population bundles generated with SCIL WM atlas warping. Values are presented as mean ± standard deviation. For each metric, the arrow indicates preference: ↑ higher is better, ↓ lower is better.

Bundles	BA (↑)	Coverage			Validity		ACC (↑)
		Dice (↑)	Overlap (↑)	Overreach (↓)	VS (↑)	IS (↓)	
AF_L	0.6292 ± 0.1495	0.3523 ± 0.0667	0.5329 ± 0.0711	1.6392 ± 0.8364	0.5954 ± 0.2042	0.4046 ± 0.2042	0.3077 ± 0.0644
AF_R	0.5541 ± 0.1369	0.2776 ± 0.0924	0.5788 ± 0.0928	3.2946 ± 2.3145	0.4686 ± 0.1955	0.5314 ± 0.1955	0.3224 ± 0.0892
CC_Fr_1	0.2417 ± 0.2213	0.2709 ± 0.0329	0.2052 ± 0.0450	0.2987 ± 0.1531	0.4619 ± 0.2753	0.5381 ± 0.2753	0.1032 ± 0.0406
CC_Fr_2	0.5255 ± 0.1936	0.3143 ± 0.0540	0.2615 ± 0.0509	0.4174 ± 0.2474	0.7674 ± 0.1836	0.2326 ± 0.1836	0.1560 ± 0.0423
CC_Oc	0.5984 ± 0.2261	0.3213 ± 0.0502	0.2582 ± 0.0470	0.3502 ± 0.1354	0.7690 ± 0.1932	0.2310 ± 0.1932	0.1587 ± 0.0354
CC_Pa	0.7669 ± 0.1166	0.4004 ± 0.0415	0.3813 ± 0.0481	0.5327 ± 0.2108	0.8209 ± 0.0726	0.1791 ± 0.0726	0.2516 ± 0.0398
CC_Pr_Po	0.7306 ± 0.1673	0.3582 ± 0.0624	0.3062 ± 0.0510	0.4139 ± 0.1517	0.7544 ± 0.1748	0.2456 ± 0.1748	0.2127 ± 0.0451
CG_L	0.4952 ± 0.2307	0.2987 ± 0.0799	0.3872 ± 0.0640	1.3788 ± 0.7905	0.5585 ± 0.2572	0.4415 ± 0.2572	0.2324 ± 0.0596
CG_R	0.5821 ± 0.1856	0.2992 ± 0.0716	0.4061 ± 0.0663	1.4719 ± 0.9060	0.5927 ± 0.2088	0.4073 ± 0.2088	0.2392 ± 0.0548
FAT_L	0.4470 ± 0.1651	0.1911 ± 0.0550	0.1397 ± 0.0367	0.3451 ± 0.1503	0.7006 ± 0.2469	0.2994 ± 0.2469	0.0801 ± 0.0295
FAT_R	0.5194 ± 0.2347	0.2456 ± 0.0666	0.1896 ± 0.0483	0.3685 ± 0.1486	0.7825 ± 0.2316	0.2175 ± 0.2316	0.1118 ± 0.0328
FPT_L	0.6110 ± 0.1684	0.3147 ± 0.0713	0.2647 ± 0.0659	0.4382 ± 0.2653	0.8380 ± 0.1700	0.1620 ± 0.1700	0.1766 ± 0.0473
FPT_R	0.6065 ± 0.2053	0.3246 ± 0.0761	0.2717 ± 0.0568	0.4484 ± 0.3410	0.8121 ± 0.2032	0.1879 ± 0.2032	0.1808 ± 0.0403
IFOF_L	0.6790 ± 0.1889	0.4000 ± 0.0352	0.4085 ± 0.0548	0.6391 ± 0.2119	0.8578 ± 0.0951	0.1422 ± 0.0951	0.2428 ± 0.0470
IFOF_R	0.7177 ± 0.1820	0.4146 ± 0.0332	0.4208 ± 0.0465	0.6104 ± 0.1384	0.8627 ± 0.1090	0.1373 ± 0.1090	0.2535 ± 0.0404
ILF_L	0.7083 ± 0.1922	0.4029 ± 0.0689	0.3942 ± 0.0632	0.6187 ± 0.4298	0.8509 ± 0.1859	0.1491 ± 0.1859	0.2217 ± 0.0472
ILF_R	0.6627 ± 0.1771	0.4006 ± 0.0592	0.3969 ± 0.0474	0.6194 ± 0.3238	0.7953 ± 0.1801	0.2047 ± 0.1801	0.2203 ± 0.0332
MCP	0.6261 ± 0.2238	0.4197 ± 0.0866	0.5127 ± 0.1259	0.9374 ± 0.4264	0.8406 ± 0.1717	0.1594 ± 0.1717	0.3127 ± 0.0838
MdLF_L	0.6514 ± 0.2497	0.2961 ± 0.1042	0.3268 ± 0.0808	1.4348 ± 2.0163	0.7699 ± 0.2828	0.2301 ± 0.2828	0.1952 ± 0.0583
MdLF_R	0.7184 ± 0.2584	0.3245 ± 0.1130	0.3534 ± 0.0771	1.5316 ± 2.4494	0.8059 ± 0.2798	0.1941 ± 0.2798	0.2218 ± 0.0528
OR_ML_L	0.5774 ± 0.1592	0.3178 ± 0.0565	0.3937 ± 0.0613	1.1509 ± 0.5152	0.6314 ± 0.1724	0.3686 ± 0.1724	0.2297 ± 0.0472
OR_ML_R	0.4285 ± 0.1937	0.2546 ± 0.0889	0.4000 ± 0.1145	2.2460 ± 2.1154	0.4630 ± 0.1867	0.5370 ± 0.1867	0.2262 ± 0.0831
POPT_L	0.5974 ± 0.2028	0.2622 ± 0.0529	0.2208 ± 0.0473	0.4660 ± 0.1570	0.8217 ± 0.1960	0.1783 ± 0.1960	0.1561 ± 0.0376
POPT_R	0.6190 ± 0.1672	0.2785 ± 0.0393	0.2513 ± 0.0565	0.5452 ± 0.2428	0.7678 ± 0.1786	0.2322 ± 0.1786	0.1777 ± 0.0391
PYT_L	0.8087 ± 0.1652	0.3321 ± 0.0650	0.2333 ± 0.0573	0.1589 ± 0.0673	0.9555 ± 0.0836	0.0445 ± 0.0836	0.1694 ± 0.0462
PYT_R	0.8449 ± 0.1374	0.3365 ± 0.0643	0.2359 ± 0.0557	0.1547 ± 0.0550	0.9738 ± 0.0504	0.0262 ± 0.0504	0.1689 ± 0.0427
SLF_L	0.5144 ± 0.1938	0.2685 ± 0.0975	0.4601 ± 0.0873	2.6943 ± 2.5927	0.5086 ± 0.2261	0.4914 ± 0.2261	0.2471 ± 0.0642
SLF_R	0.4511 ± 0.2167	0.2915 ± 0.0947	0.3881 ± 0.0661	1.7064 ± 1.4795	0.5328 ± 0.2714	0.4672 ± 0.2714	0.1991 ± 0.0526
UF_L	0.4147 ± 0.2847	0.2466 ± 0.1029	0.3379 ± 0.0897	2.0120 ± 1.7374	0.4868 ± 0.3153	0.5132 ± 0.3153	0.1992 ± 0.0563
UF_R	0.5148 ± 0.2697	0.2806 ± 0.0986	0.3496 ± 0.0690	1.6912 ± 2.0572	0.5681 ± 0.3401	0.4319 ± 0.3401	0.2092 ± 0.0535

Tab. S6 | Similarity, coverage, and validity of bundles generated with TractSeg. Values are presented as mean ± standard deviation. For each metric, the arrow indicates preference: ↑ higher is better, ↓ lower is better.

Bundles	BA (↑)	Coverage			Validity		ACC (↑)
		Dice (↑)	Overlap (↑)	Overreach (↓)	VS (↑)	IS (↓)	
AF_L	0.5724 ± 0.1948	0.3913 ± 0.0866	0.3499 ± 0.1043	0.4142 ± 0.1727	0.5853 ± 0.1914	0.4147 ± 0.1914	0.2762 ± 0.0959
AF_R	0.6474 ± 0.1659	0.3430 ± 0.0902	0.3785 ± 0.1442	0.8125 ± 0.5997	0.6952 ± 0.1769	0.3048 ± 0.1769	0.2979 ± 0.1215
CG_L	0.6135 ± 0.2226	0.4357 ± 0.0808	0.5109 ± 0.0899	0.8959 ± 0.5376	0.5451 ± 0.2249	0.4549 ± 0.2249	0.4078 ± 0.0794
CG_R	0.7085 ± 0.1142	0.4719 ± 0.0773	0.5252 ± 0.0821	0.7566 ± 0.4827	0.5784 ± 0.1563	0.4216 ± 0.1563	0.4143 ± 0.0653
FPT_L	0.6786 ± 0.1733	0.4165 ± 0.0940	0.3099 ± 0.0874	0.1680 ± 0.1442	0.7904 ± 0.1814	0.2096 ± 0.1814	0.2668 ± 0.0779
FPT_R	0.6602 ± 0.2044	0.4193 ± 0.0777	0.3131 ± 0.0827	0.1724 ± 0.1677	0.7976 ± 0.2140	0.2024 ± 0.2140	0.2713 ± 0.0727
IFOF_L	0.4519 ± 0.1644	0.3111 ± 0.0914	0.2176 ± 0.0728	0.1643 ± 0.0728	0.7510 ± 0.1899	0.2490 ± 0.1899	0.1770 ± 0.0651
IFOF_R	0.5312 ± 0.2162	0.3354 ± 0.0853	0.2372 ± 0.0742	0.1509 ± 0.0656	0.7783 ± 0.1998	0.2217 ± 0.1998	0.1910 ± 0.0656
ILF_L	0.6701 ± 0.1553	0.2837 ± 0.0808	0.1901 ± 0.0768	0.1192 ± 0.1007	0.8825 ± 0.1192	0.1175 ± 0.1192	0.1577 ± 0.0663
ILF_R	0.6213 ± 0.1360	0.2893 ± 0.0620	0.1889 ± 0.0484	0.1090 ± 0.0743	0.8387 ± 0.1393	0.1613 ± 0.1393	0.1527 ± 0.0440
MCP	0.7205 ± 0.1817	0.5069 ± 0.0865	0.4824 ± 0.1200	0.4119 ± 0.2317	0.7570 ± 0.1241	0.2430 ± 0.1241	0.3967 ± 0.1091
MdLF_L	0.5075 ± 0.1888	0.3049 ± 0.0999	0.4034 ± 0.0909	1.9252 ± 2.5943	0.3182 ± 0.1912	0.6818 ± 0.1912	0.3036 ± 0.0685
MdLF_R	0.5203 ± 0.1581	0.2751 ± 0.1089	0.3404 ± 0.1023	2.0328 ± 3.1140	0.3122 ± 0.1565	0.6878 ± 0.1565	0.2611 ± 0.0874
POPT_L	0.6542 ± 0.1308	0.3936 ± 0.0647	0.3128 ± 0.0756	0.2657 ± 0.1733	0.5605 ± 0.1585	0.4395 ± 0.1585	0.2701 ± 0.0648
POPT_R	0.6414 ± 0.1061	0.4076 ± 0.0702	0.3371 ± 0.0903	0.3063 ± 0.2553	0.5253 ± 0.1330	0.4747 ± 0.1330	0.2923 ± 0.0800
UF_L	0.3124 ± 0.2656	0.2592 ± 0.1110	0.2161 ± 0.0994	0.5523 ± 0.5707	0.2976 ± 0.2775	0.7024 ± 0.2775	0.1715 ± 0.0836
UF_R	0.3555 ± 0.2821	0.3054 ± 0.0943	0.2613 ± 0.1060	0.5263 ± 0.6797	0.2822 ± 0.2667	0.7178 ± 0.2667	0.1968 ± 0.0874

Tab. S7 | Geometry and connectivity of bundles generated with ATM. Absolute percent differences are presented as mean $\pm$ standard deviation. A dash '−' indicates an absent bundle.

Bundles	Geometry APD (↓)				Pearson (↑)	Connectivity APD (↓)			
	Mean Length	Span	Volume	Surface Area		Density	CPL	Efficiency	Modularity
AF_L	16.3782 ± 6.1705	11.9295 ± 9.4784	31.7351 ± 21.8256	48.1792 ± 28.5575	0.4791 ± 0.1895	71.5249 ± 43.9653	113.4541 ± 50.0194	147.1505 ± 30.8938	97.5305 ± 54.6368
AF_R	17.1918 ± 6.3730	10.4354 ± 6.7693	53.6095 ± 34.0503	64.8241 ± 34.2957	0.4818 ± 0.1450	84.7245 ± 39.3843	103.6713 ± 49.5844	162.7604 ± 19.4216	104.1599 ± 57.3431
CC_Fr_1	13.0568 ± 4.7349	12.0143 ± 9.6003	43.5380 ± 27.7355	30.6197 ± 18.7808	0.5115 ± 0.2119	77.4166 ± 75.8007	111.4594 ± 65.6328	116.9450 ± 63.9403	117.8747 ± 90.3340
CC_Fr_2	18.9026 ± 4.6200	23.4412 ± 9.4057	71.5429 ± 31.9900	33.3150 ± 19.6487	0.5941 ± 0.1848	53.6970 ± 44.9040	118.2380 ± 52.1721	120.4965 ± 46.8038	154.7775 ± 65.3555
CC_Oc	11.0030 ± 6.1956	10.9535 ± 7.9614	21.7280 ± 16.1675	29.7788 ± 16.9099	0.5395 ± 0.1194	26.4474 ± 18.3870	59.1651 ± 31.3135	119.3264 ± 24.0460	161.2685 ± 67.0146
CC_Pa	12.0806 ± 4.7389	17.5031 ± 10.8307	44.8484 ± 24.8365	23.9097 ± 18.3151	0.5634 ± 0.0817	22.6523 ± 13.1722	60.9603 ± 17.3834	91.3646 ± 23.7189	161.6402 ± 70.5580
CC_Pr_Po	14.5432 ± 6.2803	20.1826 ± 10.0380	62.9048 ± 18.3201	20.0317 ± 10.2214	0.5122 ± 0.1061	28.3413 ± 20.7427	68.2592 ± 32.2108	91.9094 ± 23.6135	179.1153 ± 45.5032
CG_L	11.7686 ± 8.1369	7.8983 ± 6.0824	43.0467 ± 31.4850	50.6015 ± 25.2450	0.5361 ± 0.1424	63.9521 ± 37.4996	80.8836 ± 57.6416	149.4564 ± 29.5670	105.5089 ± 84.1536
CG_R	14.4626 ± 5.5075	9.1331 ± 5.2334	42.4491 ± 31.5814	50.7069 ± 25.2823	0.4689 ± 0.1448	70.4776 ± 43.0928	91.8669 ± 52.1589	165.4556 ± 18.8717	119.0609 ± 76.6755
FAT_L	22.4657 ± 5.0508	16.0017 ± 5.7259	39.1771 ± 18.4583	33.0898 ± 21.5166	0.5763 ± 0.1146	36.7774 ± 45.0994	137.6900 ± 46.0749	90.8868 ± 38.1938	149.8390 ± 77.2425
FAT_R	24.5213 ± 5.0085	18.3513 ± 6.1703	33.3269 ± 21.8262	33.5119 ± 20.0700	0.6050 ± 0.1024	53.7432 ± 51.0949	135.9431 ± 51.0177	117.7373 ± 34.7561	138.4228 ± 71.0034
FPT_L	12.2648 ± 6.0247	8.7889 ± 6.0314	32.5808 ± 21.2695	29.1909 ± 25.6331	0.3836 ± 0.2600	96.7651 ± 51.7182	126.7067 ± 48.9923	162.8325 ± 34.8590	157.5421 ± 54.7153
FPT_R	12.6060 ± 6.6058	10.2867 ± 6.3087	36.9889 ± 19.8639	25.3915 ± 26.2654	0.3577 ± 0.2355	84.2431 ± 46.7789	138.3907 ± 51.2070	158.0912 ± 34.4026	108.2244 ± 66.8066
IFOF_L	8.1415 ± 5.1921	6.3258 ± 3.8312	22.8766 ± 14.6966	27.1602 ± 15.1998	0.5190 ± 0.1150	41.2266 ± 44.6142	90.4319 ± 47.4766	136.7237 ± 30.9487	99.6624 ± 59.8478
IFOF_R	6.4569 ± 3.9587	5.6194 ± 3.6281	19.9552 ± 11.0831	19.6940 ± 11.2226	0.5350 ± 0.1568	42.6251 ± 25.6825	76.4801 ± 52.7638	133.4644 ± 34.3376	119.1448 ± 53.8623
ILF_L	13.4611 ± 6.1576	8.9987 ± 5.0363	36.7725 ± 16.7823	36.6135 ± 28.1284	0.3397 ± 0.1190	49.4572 ± 44.9010	90.5238 ± 48.4326	130.3446 ± 36.1894	109.2040 ± 67.3970
ILF_R	15.7252 ± 10.8096	10.4027 ± 7.7099	34.3426 ± 19.5810	41.6718 ± 34.7760	0.3617 ± 0.1705	35.0685 ± 31.5976	75.5435 ± 33.9021	116.6163 ± 34.5262	122.6967 ± 75.3571
MCP	12.7934 ± 10.3452	18.9207 ± 15.4606	41.3428 ± 30.9230	58.8321 ± 36.0299	-	-	-	-	-
MdLF_L	9.5037 ± 8.1335	8.1337 ± 6.3170	41.3472 ± 38.9247	67.5858 ± 31.4190	0.4828 ± 0.1114	58.2989 ± 37.5180	74.9776 ± 34.2174	136.8213 ± 23.7961	96.1600 ± 67.7045
MdLF_R	13.5569 ± 6.2466	8.1571 ± 7.1910	34.5415 ± 31.7768	61.2891 ± 28.3313	0.4888 ± 0.1381	55.4073 ± 38.5082	73.2844 ± 24.6850	136.8928 ± 23.3823	112.0057 ± 72.1071
OR_ML_L	8.6944 ± 5.5935	8.0702 ± 5.2010	36.0904 ± 27.1998	53.1422 ± 24.7711	0.5025 ± 0.1239	43.8691 ± 36.3039	73.8708 ± 28.7495	130.0176 ± 27.2128	93.7145 ± 64.2978
OR_ML_R	6.5757 ± 5.3304	9.1249 ± 6.4066	59.6100 ± 30.9532	69.7776 ± 25.9030	0.3820 ± 0.1415	42.5585 ± 37.0258	72.2283 ± 30.7377	129.8328 ± 25.5756	62.2036 ± 59.0656
POPT_L	11.8635 ± 5.1048	7.9826 ± 4.4917	27.9131 ± 16.9057	24.9565 ± 15.8569	0.3151 ± 0.1891	56.1726 ± 42.1884	88.7277 ± 57.0255	127.6431 ± 47.9636	83.6436 ± 73.2118
POPT_R	12.8781 ± 4.3365	10.5900 ± 4.6060	30.6801 ± 19.0879	29.4745 ± 20.2749	0.2805 ± 0.1576	58.3800 ± 56.2500	115.2927 ± 47.8857	149.0068 ± 27.3337	87.8550 ± 57.3309
PYT_L	12.5660 ± 6.8230	10.3589 ± 6.6500	52.9165 ± 16.2892	11.0647 ± 9.7582	0.3818 ± 0.2085	59.2035 ± 51.5608	112.3338 ± 57.5605	118.9285 ± 47.3877	111.2434 ± 69.3786
PYT_R	10.5912 ± 6.6160	8.9615 ± 6.4546	51.4753 ± 13.1687	13.7194 ± 7.5828	0.3414 ± 0.2046	50.9705 ± 39.8734	120.1050 ± 45.0122	133.3500 ± 33.4591	83.5500 ± 63.0634
SLF_L	16.0281 ± 8.4210	10.6847 ± 8.2703	48.6050 ± 29.7418	69.8918 ± 27.8327	0.3965 ± 0.1766	113.5328 ± 53.9868	132.9065 ± 50.1194	175.9793 ± 16.6950	126.1223 ± 68.6660
SLF_R	14.1764 ± 7.4493	7.7023 ± 6.8997	43.9492 ± 29.5444	59.8345 ± 32.0203	0.4961 ± 0.1586	68.8449 ± 48.7939	102.3647 ± 44.0100	154.9991 ± 22.3582	108.8233 ± 66.3844
UF_L	9.5315 ± 7.0647	10.9449 ± 8.8163	48.5047 ± 25.9682	66.7306 ± 30.4244	0.5044 ± 0.1572	91.2972 ± 62.8138	68.1320 ± 59.6366	133.9668 ± 51.3670	106.1518 ± 83.9562
UF_R	12.1006 ± 6.5600	10.9832 ± 7.3216	30.0130 ± 26.1309	46.0246 ± 31.3479	0.4548 ± 0.1819	63.6916 ± 67.1990	70.4086 ± 44.0482	105.7981 ± 56.7679	132.1564 ± 74.7255

Tab. S8 | Geometry and connectivity of bundles generated with ATM-Population. Absolute percent differences are presented as mean ± standard deviation. A dash '−' indicates an absent bundle.

Bundles	Geometry APD (↓)				Pearson (↑)	Connectivity APD (↓)			
	Mean Length	Span	Volume	Surface Area		Density	CPL	Efficiency	Modularity
AF_L	15.1903 ± 7.4526	14.6485 ± 11.5812	35.9787 ± 22.1739	43.4696 ± 32.6299	0.3844 ± 0.1669	67.3214 ± 46.1348	113.9775 ± 52.1029	143.0450 ± 32.6974	87.3707 ± 64.9730
AF_R	14.9581 ± 8.4364	12.7552 ± 6.8572	65.1228 ± 43.3370	68.9540 ± 39.7595	0.4326 ± 0.1512	90.5512 ± 40.3479	95.0792 ± 54.3016	160.9776 ± 26.3262	104.5312 ± 53.8798
CC_Fr_1	15.8534 ± 7.9409	17.8287 ± 15.5669	65.6969 ± 49.4697	44.6863 ± 45.4319	0.5193 ± 0.1558	85.7088 ± 72.6479	135.3560 ± 59.4086	110.8318 ± 66.5151	97.0505 ± 98.3693
CC_Fr_2	12.7780 ± 9.5932	17.6194 ± 20.0278	84.8404 ± 46.4618	48.6147 ± 39.9566	0.6440 ± 0.1734	60.6623 ± 58.4476	99.9237 ± 52.4488	120.8715 ± 51.8214	147.7213 ± 70.1557
CC_Oc	15.7544 ± 7.5255	13.2561 ± 9.9175	42.8328 ± 21.7515	20.8672 ± 14.5946	0.4249 ± 0.1506	32.7311 ± 18.7321	59.9813 ± 31.8156	107.1783 ± 27.0847	161.8904 ± 65.3469
CC_Pa	17.3933 ± 9.1248	16.3454 ± 17.5016	67.6609 ± 24.2470	27.6428 ± 18.0493	0.4745 ± 0.1174	29.8949 ± 18.3837	63.4011 ± 21.1566	86.5678 ± 21.7243	166.8034 ± 62.7219
CC_Pr_Po	18.7061 ± 11.2731	17.3071 ± 15.8513	81.3522 ± 25.8039	30.0435 ± 19.3799	0.4794 ± 0.1305	36.1153 ± 24.9189	74.5614 ± 28.3515	81.3426 ± 22.1037	164.6621 ± 67.0292
CG_L	15.8380 ± 10.0238	11.5765 ± 7.6402	35.5273 ± 27.2557	40.3387 ± 24.7498	0.4735 ± 0.1800	55.3990 ± 44.8968	94.4838 ± 56.4260	137.0613 ± 35.8240	104.3670 ± 88.3807
CG_R	16.2125 ± 7.7357	12.2955 ± 7.4877	32.7588 ± 30.4698	44.2456 ± 25.7716	0.4075 ± 0.1931	69.2777 ± 45.5215	110.7252 ± 46.9127	158.4514 ± 24.6276	135.7073 ± 76.8905
FAT_L	19.0706 ± 8.8019	18.9685 ± 8.8322	66.9717 ± 42.2065	31.3912 ± 39.8544	0.5329 ± 0.1505	28.4296 ± 23.1792	121.1555 ± 49.5196	78.0864 ± 36.0665	144.6450 ± 79.1146
FAT_R	17.7398 ± 11.7693	16.4434 ± 11.1929	62.4829 ± 45.6368	37.0495 ± 34.5284	0.5468 ± 0.1407	48.0135 ± 38.4612	111.2467 ± 53.1970	106.0854 ± 39.8015	133.3885 ± 77.2468
FPT_L	15.9829 ± 7.1604	16.9720 ± 6.7668	31.0011 ± 20.7129	30.4112 ± 23.2669	0.3394 ± 0.2522	90.4185 ± 51.1181	140.1449 ± 38.4490	162.8316 ± 23.5165	144.8132 ± 66.1390
FPT_R	15.1898 ± 7.8461	18.0467 ± 7.5642	36.2902 ± 19.5963	27.9517 ± 23.8032	0.3533 ± 0.2401	93.4268 ± 49.2626	154.6930 ± 39.0232	166.9447 ± 22.4762	116.1346 ± 60.8716
IFOF_L	9.5513 ± 6.2685	7.0166 ± 5.1567	31.6527 ± 20.8527	14.0585 ± 10.0268	0.4649 ± 0.1078	49.2110 ± 50.8075	105.3128 ± 45.2055	131.4743 ± 33.2149	123.8310 ± 58.5869
IFOF_R	6.9876 ± 4.3555	7.5880 ± 5.4508	22.7739 ± 15.3100	19.0003 ± 9.2328	0.4848 ± 0.1854	51.1656 ± 34.7996	86.0917 ± 55.8142	127.0845 ± 36.4778	118.8417 ± 59.7063
ILF_L	14.4210 ± 6.3183	10.5947 ± 5.5977	43.9348 ± 18.0770	20.4695 ± 21.6554	0.2617 ± 0.1143	50.0603 ± 48.5389	109.1577 ± 48.4228	130.5510 ± 36.0233	118.0520 ± 65.4610
ILF_R	11.2728 ± 5.4289	7.9269 ± 5.3997	33.9551 ± 18.9097	19.3101 ± 16.6968	0.3127 ± 0.1335	26.8452 ± 26.0498	95.8546 ± 37.9261	124.1816 ± 21.0852	119.7859 ± 77.0494
MCP	14.9683 ± 11.2561	17.8836 ± 14.9682	32.5357 ± 23.0735	44.0487 ± 36.1709	-	-	-	-	-
MdLF_L	19.8016 ± 10.0455	17.9470 ± 8.6171	55.5269 ± 48.8826	50.5459 ± 32.5481	0.4016 ± 0.1465	63.3060 ± 41.2535	88.6555 ± 38.8299	135.4596 ± 28.6010	97.6667 ± 68.5319
MdLF_R	19.0719 ± 9.7041	16.4447 ± 10.7364	55.1219 ± 51.5649	55.8986 ± 41.4869	0.4476 ± 0.1573	65.5988 ± 45.3818	78.6727 ± 27.0928	138.5169 ± 31.4171	117.2157 ± 67.0735
OR_ML_L	9.9549 ± 5.8899	9.2682 ± 5.6871	33.4464 ± 24.4813	33.2180 ± 24.2768	0.4035 ± 0.1988	44.1277 ± 35.3642	70.0507 ± 34.3818	116.7809 ± 35.4810	112.1680 ± 63.4526
OR_ML_R	6.4981 ± 6.2865	11.4850 ± 6.7212	57.7529 ± 43.6939	54.3059 ± 37.4020	0.2694 ± 0.1658	43.6361 ± 45.2740	67.2715 ± 35.4010	118.9910 ± 37.4315	68.3848 ± 60.4036
POPT_L	20.9558 ± 8.3149	22.6443 ± 7.0660	42.3865 ± 37.4088	25.3714 ± 32.0418	0.2751 ± 0.1665	68.0210 ± 57.2305	119.9637 ± 44.6489	149.1643 ± 29.2007	98.2046 ± 75.6435
POPT_R	18.0135 ± 7.0777	23.1338 ± 7.7626	42.9306 ± 36.2515	27.7317 ± 31.1224	0.2708 ± 0.1782	71.9133 ± 59.4201	131.8678 ± 50.0063	149.8203 ± 31.8575	97.7578 ± 69.3978
PYT_L	19.6281 ± 10.1564	23.6654 ± 9.0217	63.4540 ± 31.9547	21.2143 ± 30.5960	0.2747 ± 0.1685	60.8503 ± 54.6519	138.8670 ± 44.2535	132.3403 ± 39.4252	126.1570 ± 65.5734
PYT_R	13.6734 ± 8.1576	17.4876 ± 8.8137	47.8747 ± 14.9153	14.6446 ± 9.9401	0.3635 ± 0.2185	52.6821 ± 54.5900	133.0168 ± 47.4381	143.4494 ± 27.6561	95.3623 ± 66.7776
SLF_L	11.9386 ± 8.5120	12.3235 ± 8.6830	73.4610 ± 50.5316	79.9947 ± 40.0558	0.3565 ± 0.1669	95.2981 ± 58.8599	92.1867 ± 63.8907	161.9181 ± 38.4239	97.0878 ± 71.7606
SLF_R	11.8938 ± 6.9515	8.5291 ± 8.4121	53.3716 ± 36.9150	52.5401 ± 37.7580	0.4816 ± 0.1593	63.9322 ± 51.4226	92.9967 ± 55.1508	148.2471 ± 26.3525	113.8902 ± 72.1118
UF_L	23.6261 ± 10.5987	13.6764 ± 9.6093	69.5496 ± 47.5604	61.6631 ± 33.2409	0.3876 ± 0.1714	96.6155 ± 65.2664	120.5505 ± 52.3327	148.1419 ± 38.7502	143.3888 ± 78.5278
UF_R	17.9449 ± 6.2685	13.6795 ± 10.1713	52.6053 ± 46.9771	52.8957 ± 35.9998	0.3329 ± 0.1779	76.0534 ± 67.1183	106.8625 ± 54.9917	133.0591 ± 46.0476	140.4326 ± 83.3807

Tab. S9 | Geometry and connectivity of bundles generated from MRtrix with BundleSeg. Absolute percent differences are presented as mean ± standard deviation. A dash '−' indicates an absent bundle.

Bundles	Geometry APD (↓)				Pearson (↑)	Connectivity APD (↓)			
	Mean Length	Span	Volume	Surface Area		Density	CPL	Efficiency	Modularity
AF_L	28.8638 ± 6.2594	13.8137 ± 9.3045	87.9822 ± 50.4303	68.7963 ± 44.9017	0.4425 ± 0.2088	56.2553 ± 38.0790	115.9907 ± 50.9674	92.1982 ± 51.5560	105.2410 ± 73.4941
AF_R	29.3859 ± 6.8291	17.1100 ± 13.3408	73.9471 ± 53.0796	58.2679 ± 44.7810	0.4297 ± 0.2057	44.3435 ± 37.8135	120.6064 ± 49.0598	115.1491 ± 43.6608	97.9310 ± 70.8317
CC_Fr_1	5.7839 ± 3.7027	43.7240 ± 20.1852	78.7807 ± 55.1777	54.2505 ± 54.1674	0.0850 ± 0.1963	112.9959 ± 92.1093	32.3852 ± 55.1185	110.0034 ± 92.7009	95.4459 ± 99.6944
CC_Fr_2	2.7875 ± 2.6557	77.9988 ± 20.6141	44.1374 ± 44.1312	45.1637 ± 37.7295	0.4173 ± 0.2821	84.4714 ± 59.0603	78.1880 ± 57.8595	76.5083 ± 62.6640	133.0280 ± 82.0938
CC_Oc	35.7338 ± 4.4085	32.8104 ± 17.8196	177.0229 ± 19.2431	156.8045 ± 23.9637	0.2808 ± 0.2287	168.4303 ± 26.6293	75.6919 ± 47.6330	121.8974 ± 63.8347	104.2370 ± 97.1341
CC_Pa	14.3383 ± 4.2941	32.4736 ± 13.3728	77.7381 ± 41.4743	48.1654 ± 34.0950	0.4698 ± 0.1117	38.9257 ± 40.9147	60.3066 ± 21.8955	73.7371 ± 33.9708	169.2051 ± 59.6969
CC_Pr_Po	12.9030 ± 4.1850	67.7001 ± 17.2934	29.1074 ± 35.7807	26.2781 ± 27.9477	0.4167 ± 0.1398	39.0192 ± 40.9505	46.9349 ± 31.5004	88.4911 ± 23.5332	164.8780 ± 67.2973
CG_L	17.8938 ± 13.7226	14.0711 ± 11.9538	69.1726 ± 23.1860	75.6729 ± 21.5253	0.4303 ± 0.1663	110.8540 ± 48.8508	107.3108 ± 46.5270	172.8764 ± 23.9367	136.6753 ± 65.7551
CG_R	16.7167 ± 17.4993	12.2994 ± 14.1988	80.6849 ± 28.6274	85.7612 ± 24.1933	0.3165 ± 0.1820	125.1664 ± 42.2362	113.3683 ± 50.5499	179.1848 ± 19.0918	157.8087 ± 63.9693
FAT_L	5.2375 ± 3.7237	8.1201 ± 5.1925	41.3541 ± 42.6404	30.0200 ± 37.0744	0.3467 ± 0.2989	117.9954 ± 64.1643	112.7302 ± 55.6808	82.4727 ± 86.1731	78.5563 ± 94.9835
FAT_R	5.3931 ± 4.0602	7.0737 ± 4.7337	33.0693 ± 44.4301	31.7190 ± 41.1119	0.3900 ± 0.2727	99.1829 ± 67.7925	101.1498 ± 59.5998	91.7444 ± 65.1141	126.3478 ± 91.3138
FPT_L	21.8017 ± 8.4264	18.0467 ± 7.5873	129.1507 ± 35.1542	100.6918 ± 37.1380	0.2330 ± 0.2834	110.6044 ± 64.7949	73.4742 ± 58.0104	97.6367 ± 67.7734	120.6616 ± 94.9560
FPT_R	21.1294 ± 7.9463	17.3374 ± 7.5307	117.0144 ± 47.3696	92.9250 ± 45.1590	0.2453 ± 0.2493	99.7060 ± 60.4912	98.5897 ± 62.4057	81.1510 ± 70.6249	137.8028 ± 87.3037
IFOF_L	41.6731 ± 8.0817	33.0382 ± 8.3303	146.0696 ± 24.1083	137.6828 ± 21.9983	0.2132 ± 0.1760	120.9023 ± 38.4317	127.9149 ± 56.9797	61.3328 ± 53.1011	161.9909 ± 59.4537
IFOF_R	37.8219 ± 8.1011	28.2536 ± 8.8492	143.9668 ± 23.8343	139.3631 ± 19.7904	0.2836 ± 0.1989	92.4370 ± 60.3363	114.7265 ± 55.7990	87.1881 ± 41.8378	161.8784 ± 48.8645
ILF_L	15.8867 ± 6.4041	11.8636 ± 6.1887	31.7209 ± 33.2591	30.6702 ± 31.6747	0.3473 ± 0.1449	39.3282 ± 43.0886	80.9569 ± 35.1834	108.2052 ± 40.1258	122.3668 ± 73.5356
ILF_R	14.7783 ± 6.1654	11.5663 ± 5.0512	33.2335 ± 32.3118	32.5480 ± 31.8193	0.3683 ± 0.1289	26.4232 ± 28.0865	70.8102 ± 34.0117	103.4886 ± 31.6855	142.5396 ± 62.3275
MCP	28.2028 ± 10.7673	22.4281 ± 23.0801	146.4465 ± 61.9727	125.2056 ± 54.0225	-	-	-	-	-
MdLF_L	8.7013 ± 6.9419	8.6567 ± 7.7469	51.6726 ± 33.2609	39.0602 ± 29.6036	0.4715 ± 0.1562	42.1907 ± 33.0752	85.1519 ± 36.7559	105.0234 ± 37.3388	104.6511 ± 66.9734
MdLF_R	6.3598 ± 4.8500	6.6968 ± 4.8696	57.4872 ± 46.5521	47.8694 ± 39.0933	0.4932 ± 0.1539	53.7005 ± 37.4463	68.7817 ± 20.6471	118.9414 ± 40.5299	116.3487 ± 70.6585
OR_ML_L	5.0850 ± 4.4191	5.8998 ± 4.8042	53.9135 ± 32.0633	31.1518 ± 22.3581	0.3963 ± 0.1263	54.2445 ± 21.1140	74.9794 ± 30.5771	74.2082 ± 36.6191	85.3263 ± 66.2089
OR_ML_R	6.1623 ± 7.3072	8.2874 ± 7.1831	63.4450 ± 40.7029	37.5376 ± 31.3197	0.2923 ± 0.1551	63.8084 ± 39.9575	77.1344 ± 34.6310	79.4531 ± 48.7820	96.9684 ± 57.8442
POPT_L	19.0844 ± 5.3474	19.3754 ± 6.5343	120.3868 ± 40.6702	94.4559 ± 34.5649	0.3025 ± 0.2467	121.6281 ± 54.9378	73.1873 ± 55.7223	82.3611 ± 69.6629	105.3239 ± 77.2181
POPT_R	18.8785 ± 4.7430	20.5395 ± 4.8500	115.9403 ± 44.8431	93.6892 ± 34.9535	0.3039 ± 0.2123	99.3844 ± 62.0769	73.5053 ± 60.3639	77.7852 ± 61.7575	115.3905 ± 78.0092
PYT_L	17.0738 ± 7.7422	15.1706 ± 8.3321	88.5464 ± 36.6111	72.6984 ± 34.1138	0.3723 ± 0.2440	117.2104 ± 60.4074	82.2833 ± 64.3473	91.9005 ± 66.2040	108.1071 ± 92.0837
PYT_R	17.5932 ± 7.2573	16.5858 ± 7.6242	91.4248 ± 27.7694	74.7841 ± 24.3140	0.3465 ± 0.2431	115.0554 ± 56.7531	77.4424 ± 64.5653	83.1131 ± 65.8201	115.0921 ± 75.0838
SLF_L	26.7878 ± 36.5661	24.5800 ± 36.9272	75.9983 ± 53.6756	62.5151 ± 49.2701	0.3507 ± 0.2348	101.9027 ± 56.8448	101.5612 ± 59.8188	159.8084 ± 44.2256	103.7727 ± 71.2417
SLF_R	23.1124 ± 36.9801	18.8120 ± 37.7264	64.1922 ± 56.0286	57.3220 ± 48.9318	0.5139 ± 0.1740	67.5001 ± 55.2840	110.9925 ± 52.5646	147.4819 ± 32.0273	98.8109 ± 73.5318
UF_L	7.9091 ± 9.8992	15.3006 ± 12.0420	76.9889 ± 45.3026	56.9821 ± 41.7263	0.2605 ± 0.2369	57.4282 ± 70.8179	56.1935 ± 51.6789	97.5877 ± 65.2284	102.2076 ± 84.5329
UF_R	6.3512 ± 6.0436	12.6905 ± 9.1482	52.6721 ± 43.3370	47.1954 ± 37.9493	0.3177 ± 0.2492	59.0160 ± 68.4713	22.7814 ± 18.9467	93.2299 ± 62.8052	88.8137 ± 77.5006

Tab. S10 | Geometry and connectivity of bundles generated with SCIL WM atlas warping. Absolute percent differences are presented as mean ± standard deviation. A dash '−' indicates an absent bundle.

Bundles	Geometry APD (↓)				Pearson (↑)	Connectivity APD (↓)			
	Mean Length	Span	Volume	Surface Area		Density	CPL	Efficiency	Modularity
AF_L	6.8671 ± 5.1370	12.9578 ± 9.6855	80.4248 ± 31.5824	65.8603 ± 29.4192	0.5117 ± 0.1435	65.3658 ± 54.7625	72.8656 ± 45.7535	139.0153 ± 37.6715	113.2092 ± 59.4608
AF_R	6.2998 ± 5.2393	11.7393 ± 6.2468	110.7677 ± 43.0565	87.1237 ± 37.6548	0.4812 ± 0.1484	87.0591 ± 34.9837	59.9066 ± 41.0402	163.0339 ± 17.7238	105.9860 ± 60.0702
CC_Fr_1	15.3850 ± 5.3386	69.1439 ± 14.0994	52.5735 ± 29.4224	28.1593 ± 21.1176	0.2810 ± 0.0934	120.0259 ± 91.4889	36.0125 ± 56.2544	113.0662 ± 92.8543	86.7505 ± 98.9151
CC_Fr_2	6.7779 ± 4.2808	79.2540 ± 17.3526	34.3718 ± 26.8075	34.7595 ± 18.6882	0.3184 ± 0.3133	93.9333 ± 54.4729	85.7562 ± 61.1966	57.1404 ± 59.0518	125.5198 ± 86.6299
CC_Oc	5.8269 ± 4.1057	26.1639 ± 11.7008	31.4334 ± 19.6175	34.0720 ± 15.8905	0.3774 ± 0.1972	55.4983 ± 42.8273	45.2238 ± 29.1175	75.1446 ± 36.5914	177.0998 ± 45.6462
CC_Pa	5.0622 ± 4.3203	69.1572 ± 13.2717	24.1413 ± 17.6771	39.7097 ± 23.2796	0.4106 ± 0.1169	43.7868 ± 16.2084	60.3170 ± 16.2961	73.3030 ± 24.1981	162.7212 ± 69.0877
CC_Pr_Po	7.2905 ± 3.6814	76.4518 ± 15.2194	20.2895 ± 10.3475	19.0045 ± 12.5899	0.4542 ± 0.1735	62.5391 ± 36.8505	52.4721 ± 36.9441	68.2240 ± 38.0621	165.5790 ± 65.4351
CG_L	9.3606 ± 7.1440	7.1445 ± 5.4248	65.1877 ± 32.5148	54.4920 ± 26.2758	0.3232 ± 0.2259	68.1916 ± 43.3152	73.1483 ± 58.6951	154.2046 ± 28.2237	120.0022 ± 74.5257
CG_R	7.5502 ± 4.7267	5.5390 ± 3.7249	70.8563 ± 28.5890	57.4128 ± 24.9052	0.1967 ± 0.1724	88.1679 ± 41.3946	79.2521 ± 61.9854	165.4979 ± 21.3220	169.8746 ± 51.8653
FAT_L	21.2143 ± 4.4211	27.4959 ± 4.9851	61.4267 ± 22.5394	40.8125 ± 15.7842	0.2899 ± 0.1970	147.8708 ± 65.1646	114.3985 ± 58.8828	131.7010 ± 76.2037	57.5395 ± 86.2745
FAT_R	18.0987 ± 6.1302	22.9826 ± 8.6943	47.2526 ± 22.3038	33.9039 ± 18.4856	0.3415 ± 0.1170	136.1283 ± 66.4507	111.4051 ± 59.7472	112.0758 ± 80.1887	117.4574 ± 98.2727
FPT_L	6.4878 ± 4.9733	5.1643 ± 5.2063	28.3680 ± 19.1025	24.6312 ± 15.9789	0.2449 ± 0.2240	140.3164 ± 72.0895	68.1552 ± 63.2570	132.9945 ± 69.4190	124.9392 ± 96.7776
FPT_R	5.3322 ± 4.2567	4.8863 ± 5.2827	30.3603 ± 20.3129	20.4533 ± 16.1621	0.2556 ± 0.2291	134.5413 ± 66.5576	70.5679 ± 61.7485	114.2451 ± 75.5957	149.5326 ± 82.3574
IFOF_L	5.5881 ± 4.1287	9.4986 ± 5.5994	28.1587 ± 19.2804	18.2430 ± 10.9580	0.4457 ± 0.1790	63.6361 ± 30.7008	46.5834 ± 40.0148	69.5504 ± 37.9557	139.7234 ± 64.4818
IFOF_R	7.7613 ± 5.6856	13.3749 ± 8.2729	24.2648 ± 15.8628	14.9966 ± 9.9397	0.5059 ± 0.1928	70.2414 ± 38.9035	56.6767 ± 46.5375	72.2860 ± 42.7081	131.4996 ± 70.1214
ILF_L	7.8329 ± 4.6974	5.2944 ± 3.6101	29.3315 ± 26.3861	23.8331 ± 17.6178	0.2635 ± 0.1469	54.2988 ± 49.7338	56.0230 ± 40.7227	80.4280 ± 48.8916	120.3754 ± 68.8230
ILF_R	5.1991 ± 4.1538	6.1044 ± 2.7182	24.9717 ± 21.1794	17.5200 ± 14.3292	0.2726 ± 0.1521	38.4853 ± 27.7240	50.6188 ± 30.0618	90.4429 ± 29.0305	127.2172 ± 74.1863
MCP	10.5857 ± 6.3202	17.3298 ± 11.1574	51.4537 ± 30.1220	43.3855 ± 33.6452	-	-	-	-	-
MdLF_L	14.3665 ± 8.3194	8.4157 ± 5.5276	56.8422 ± 49.5838	36.7739 ± 31.1822	0.4163 ± 0.1573	50.8289 ± 30.1481	61.8143 ± 23.6356	97.4048 ± 46.8142	125.1156 ± 66.5788
MdLF_R	10.7450 ± 7.9964	8.2246 ± 5.9636	55.3305 ± 50.2630	36.1239 ± 34.6153	0.4632 ± 0.1531	45.8801 ± 36.1392	71.4388 ± 28.5265	104.3784 ± 42.4640	135.6276 ± 68.1296
OR_ML_L	4.1400 ± 3.8627	10.0678 ± 5.7946	59.0603 ± 31.4590	42.5010 ± 24.2010	0.3573 ± 0.1703	45.0228 ± 31.4545	59.9386 ± 31.6911	95.1105 ± 36.5221	88.5938 ± 69.5327
OR_ML_R	5.5102 ± 7.1274	12.7676 ± 7.6223	86.4971 ± 42.1039	62.5457 ± 34.9648	0.2138 ± 0.1196	47.8760 ± 35.7274	53.2716 ± 34.1940	111.9585 ± 39.9669	69.9509 ± 58.2121
POPT_L	4.5857 ± 3.1448	5.1290 ± 3.8614	23.6069 ± 13.4042	21.2768 ± 11.9264	0.2908 ± 0.2180	144.0642 ± 64.5311	99.7508 ± 63.4215	121.5503 ± 77.3041	126.2808 ± 88.1962
POPT_R	4.4651 ± 3.0735	5.7789 ± 4.2919	26.3258 ± 19.7795	22.0600 ± 14.7985	0.3237 ± 0.1824	125.5377 ± 67.2363	113.6394 ± 65.2036	99.3050 ± 75.7996	153.7215 ± 75.8021
PYT_L	3.8525 ± 3.7411	6.0467 ± 3.6747	63.3981 ± 21.2531	36.9747 ± 14.4668	0.4169 ± 0.2487	165.0297 ± 48.0601	99.1033 ± 68.9259	145.0567 ± 67.5981	146.3560 ± 83.8815
PYT_R	3.6086 ± 3.4015	5.9533 ± 3.4104	63.3580 ± 17.7112	35.7914 ± 12.2064	0.3720 ± 0.2331	166.4927 ± 38.0673	79.4674 ± 65.5698	133.1660 ± 68.2547	155.3007 ± 75.5170
SLF_L	11.0423 ± 7.2426	9.7943 ± 7.3305	96.2303 ± 43.7705	66.0393 ± 36.0735	0.4064 ± 0.1902	79.7213 ± 77.7249	65.8042 ± 49.2349	152.9229 ± 33.4092	121.1710 ± 75.6789
SLF_R	6.9994 ± 5.5779	11.6982 ± 6.1827	68.6048 ± 47.6555	47.0659 ± 33.1078	0.5068 ± 0.1499	53.3537 ± 31.5136	74.2599 ± 49.0805	113.8505 ± 43.8950	126.6906 ± 70.0115
UF_L	12.1606 ± 8.3958	14.4482 ± 11.9936	77.7074 ± 49.4321	43.2770 ± 33.8591	0.4197 ± 0.1874	36.2327 ± 46.3920	62.0342 ± 59.2523	61.0680 ± 57.6517	108.2708 ± 90.1743
UF_R	5.9925 ± 4.5454	13.8315 ± 10.2998	59.7495 ± 47.9775	36.6240 ± 34.8001	0.3359 ± 0.2197	69.6248 ± 57.9120	76.1830 ± 57.7207	62.0682 ± 57.6067	122.7808 ± 84.0423

Tab. S11 | Geometry and connectivity of bundles generated with TractSeg. Absolute percent differences are presented as mean ± standard deviation. A dash '−' indicates an absent bundle.

Bundles	Geometry APD (↓)				Pearson (↑)	Connectivity APD (↓)			
	Mean Length	Span	Volume	Surface Area		Density	CPL	Efficiency	Modularity
AF_L	25.9307 ± 7.1867	24.2242 ± 16.2981	31.8824 ± 21.0098	20.4990 ± 16.9734	0.3509 ± 0.1771	130.7930 ± 72.4398	159.0896 ± 43.0805	164.1861 ± 44.4708	150.2655 ± 65.7205
AF_R	14.7723 ± 7.3218	11.0559 ± 7.5228	52.7674 ± 32.2260	40.3864 ± 28.2097	0.3988 ± 0.1670	125.1083 ± 79.8707	145.3272 ± 56.5834	167.6320 ± 36.8620	160.5039 ± 60.8569
CG_L	18.7440 ± 12.7914	14.2218 ± 10.0264	38.7323 ± 28.7776	47.8650 ± 23.4019	0.3249 ± 0.1296	135.2870 ± 75.4661	141.3610 ± 70.0386	170.3936 ± 37.8569	152.3122 ± 82.8314
CG_R	22.5000 ± 10.5192	16.5651 ± 10.0830	33.9811 ± 27.1874	36.3449 ± 23.2912	0.3087 ± 0.1261	141.5595 ± 66.1656	142.2118 ± 58.8784	180.0850 ± 23.3998	136.0995 ± 80.5869
FPT_L	13.4802 ± 7.2197	9.5510 ± 8.4750	64.5067 ± 30.8731	57.5199 ± 26.4426	0.1680 ± 0.2294	158.9462 ± 63.2939	141.9863 ± 58.4294	160.8648 ± 62.5728	114.9763 ± 97.4752
FPT_R	11.5853 ± 7.1216	8.0825 ± 8.3687	67.1759 ± 27.5980	63.1457 ± 22.2177	0.2598 ± 0.1107	152.9840 ± 75.4014	133.0723 ± 54.7477	166.1270 ± 57.0859	150.7430 ± 84.0820
IFOF_L	26.7511 ± 5.9866	22.5532 ± 7.7486	86.7965 ± 22.1776	66.7327 ± 26.5753	0.5757 ± 0.1183	177.6029 ± 48.6158	176.1019 ± 45.8698	191.9643 ± 19.3959	153.1065 ± 76.6459
IFOF_R	20.6587 ± 9.8153	15.1711 ± 8.8905	86.3342 ± 27.3037	59.7589 ± 24.1881	0.6215 ± 0.0737	172.7907 ± 54.3458	190.6885 ± 7.7336	192.0111 ± 15.6011	181.3627 ± 51.0550
ILF_L	13.7164 ± 7.1017	9.9979 ± 5.2927	104.9650 ± 32.9202	97.1996 ± 28.6238	0.2580 ± 0.1279	143.1780 ± 74.5215	124.8479 ± 65.6551	146.1193 ± 68.6983	112.3669 ± 89.6710
ILF_R	15.4987 ± 5.2703	11.1992 ± 4.9452	105.8440 ± 23.1539	100.1605 ± 18.1797	0.2536 ± 0.1253	148.1518 ± 66.3389	127.4731 ± 66.1078	137.1687 ± 76.4524	116.5496 ± 88.0632
MCP	23.5557 ± 10.4028	18.2712 ± 10.1143	27.4852 ± 20.7930	31.6378 ± 20.6518	-	-	-	-	-
MdLF_L	24.7503 ± 11.2406	27.8340 ± 11.2193	59.1514 ± 53.3226	76.3347 ± 37.5720	0.1647 ± 0.1745	140.1730 ± 60.7228	153.2225 ± 45.6543	169.1587 ± 32.9949	166.2077 ± 50.8270
MdLF_R	22.8927 ± 10.1179	23.0922 ± 12.9674	54.8791 ± 53.7793	70.3347 ± 38.4399	0.2992 ± 0.1727	133.8281 ± 72.7731	140.3029 ± 56.5685	167.3518 ± 37.8551	150.1924 ± 71.9429
POPT_L	14.8860 ± 7.2696	14.0702 ± 8.4787	50.0688 ± 26.9983	36.6492 ± 19.9329	0.1577 ± 0.1431	136.1473 ± 77.0702	163.3863 ± 46.7693	158.3369 ± 58.5575	138.0840 ± 77.3743
POPT_R	17.1744 ± 5.8879	16.5471 ± 7.4166	46.3896 ± 26.6055	35.9621 ± 22.3454	0.0783 ± 0.0640	136.9786 ± 79.0322	180.9065 ± 15.7748	157.1115 ± 60.3991	141.0503 ± 67.6567
UF_L	51.4600 ± 13.1133	53.1904 ± 17.0309	60.3029 ± 34.1972	78.7776 ± 30.5903	0.2849 ± 0.1600	110.6975 ± 91.1430	121.1829 ± 77.2268	114.8551 ± 88.6648	74.8395 ± 92.6888
UF_R	47.0855 ± 16.1410	49.4637 ± 18.4856	60.7744 ± 30.4797	80.1602 ± 30.7219	0.3391 ± 0.2352	115.4410 ± 87.1842	123.9890 ± 75.5244	132.9097 ± 73.7247	114.4904 ± 89.8244

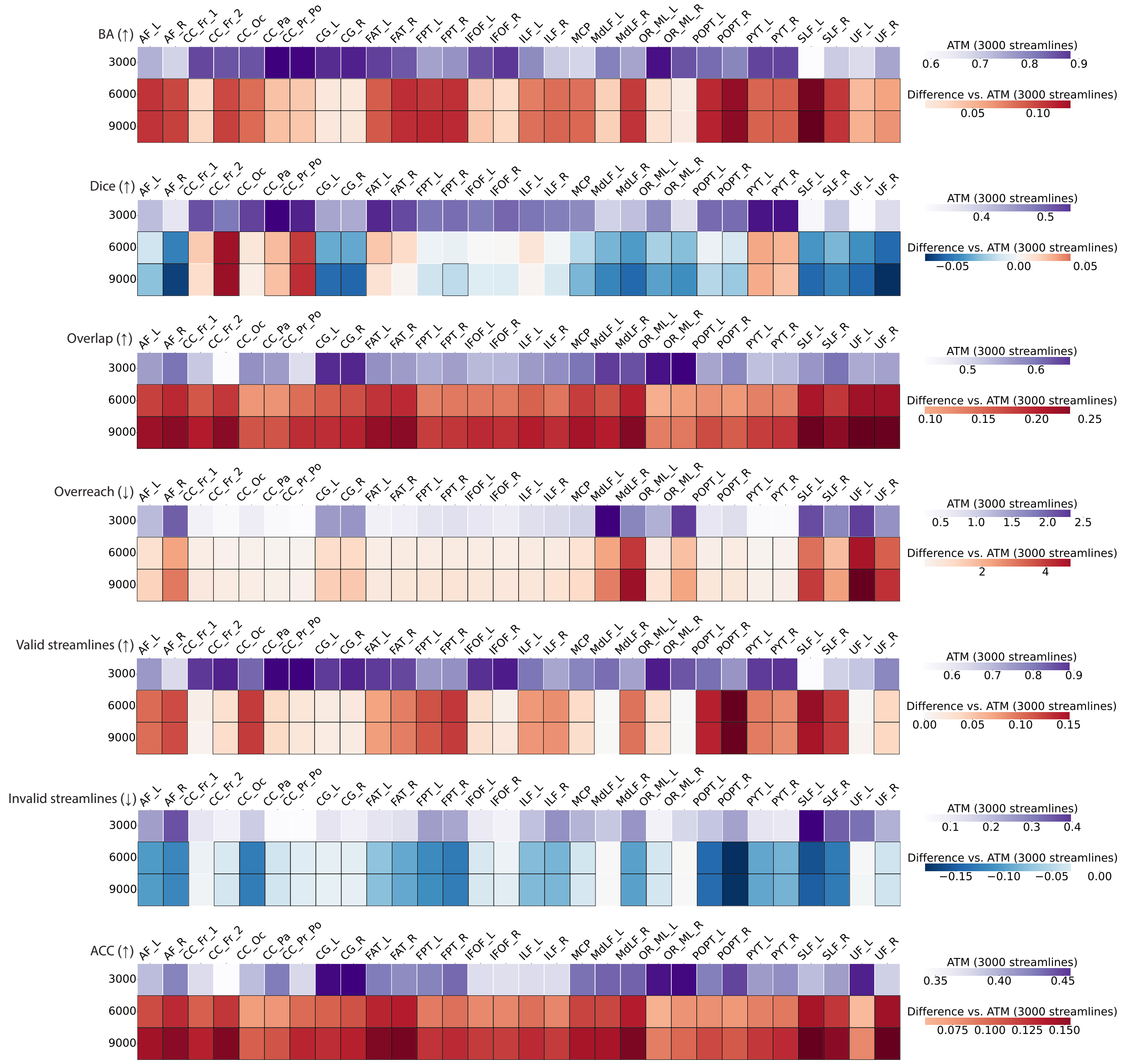

Fig. S12 | Similarity and coverage of bundles generated with ATM using 3000, 6000, and 9000 streamlines per bundle. The first row of each heatmap shows the values for 3,000 streamlines per bundle as the baseline. The subsequent rows display the paired performance differences of ATM with other streamline counts relative to this baseline. The arrow indicates preference: ↑ higher is better, ↓ lower is better. Cells outlined with squares indicate statistically significant differences.

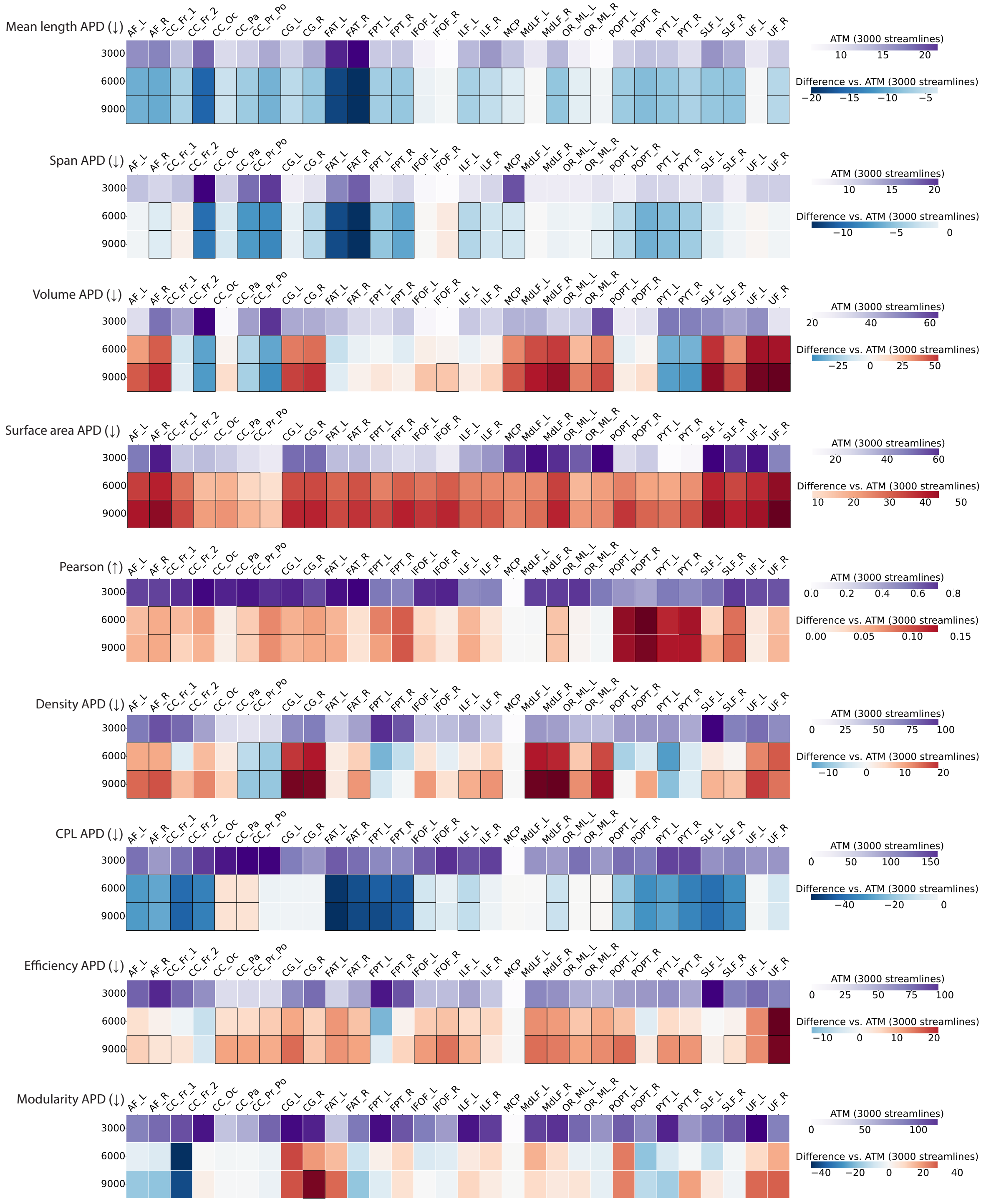

Fig. S13 | Geometry and connectivity of bundles generated with ATM using 3,000, 6,000, and 9,000 streamlines per bundle. The first row of each heatmap shows the values for 3,000 streamlines per bundle as the baseline. The subsequent rows display the paired performance differences of ATM with other streamline counts relative to this baseline. The arrow indicates preference: ↑ higher is better, ↓ lower is better. Cells outlined with squares indicate statistically significant differences.

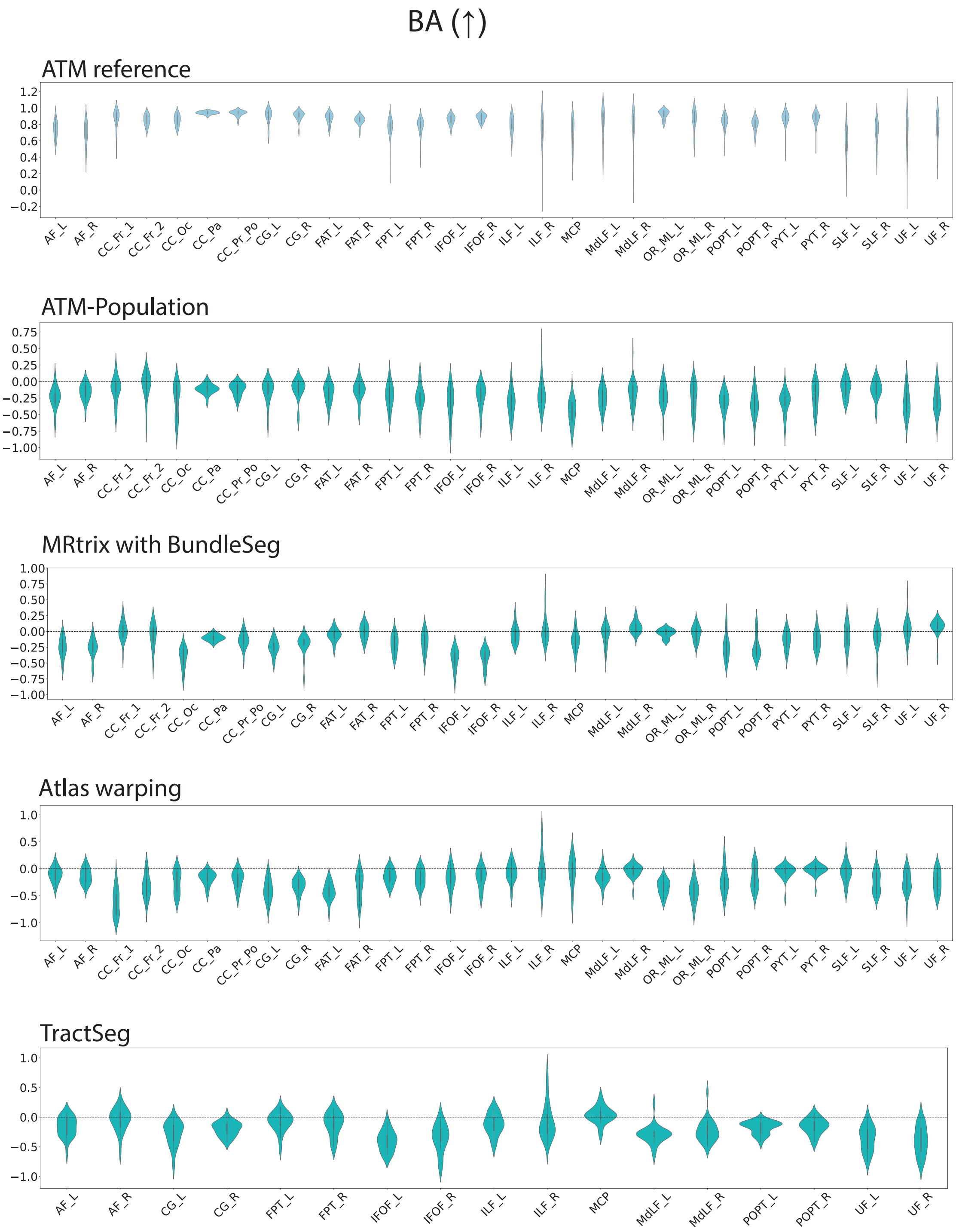

Fig. S14 | BA of bundles generated with ATM, ATM-Population, MRtrix with BundleSeg, SCIL WM atlas warping, and TractSeg. The first violin plot shows the ATM values as the baseline. The subsequent rows display the paired performance differences of other methods relative to this baseline. For each metric, the arrow indicates preference: ↑ higher is better and ↓ lower is better.

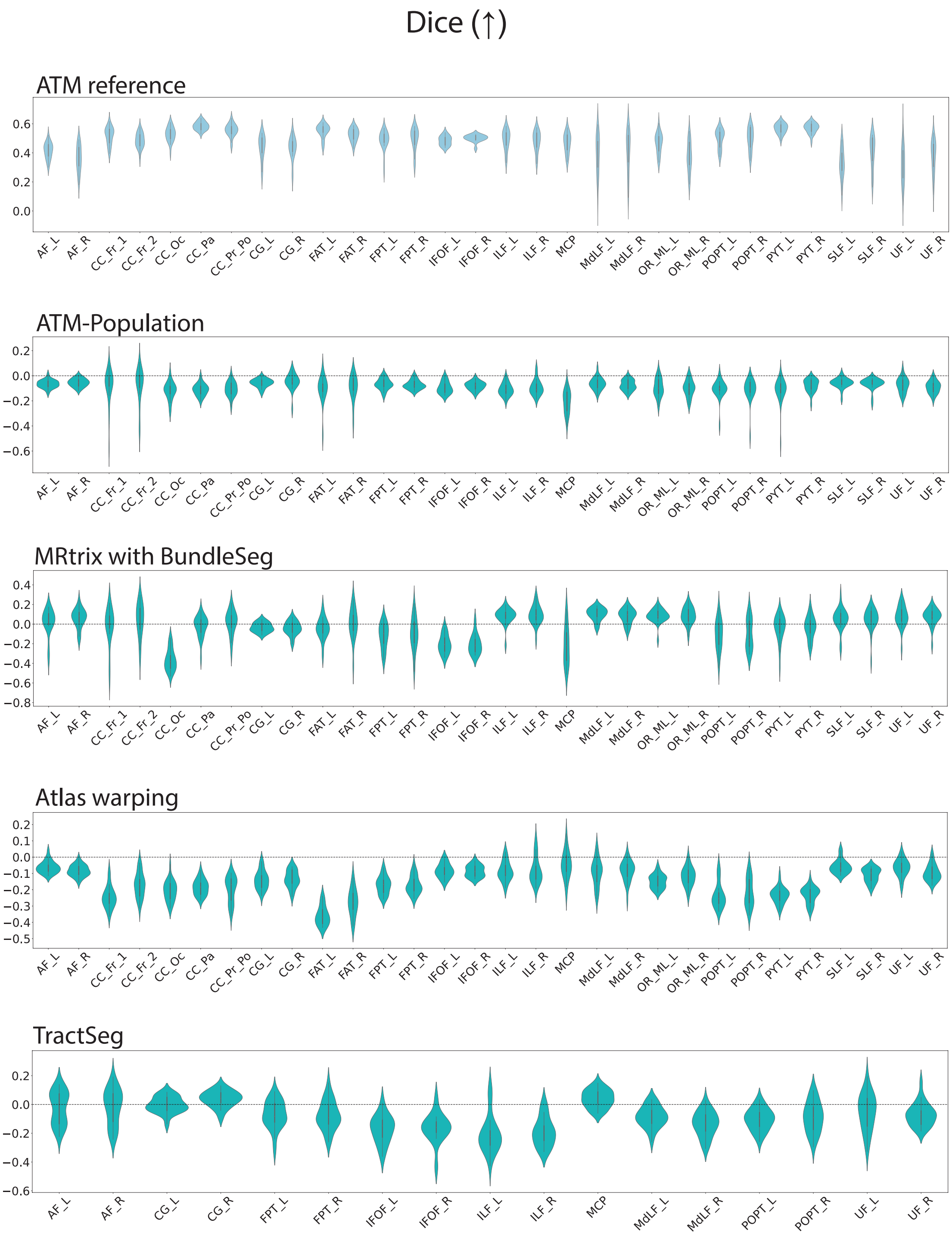

Fig. S15 | Dice scores of bundles generated with ATM, ATM-Population, MRtrix with BundleSeg, SCIL WM atlas warping, and TractSeg. The first violin plot shows the ATM values as the baseline. The subsequent rows display the paired performance differences of other methods relative to this baseline. For each metric, the arrow indicates preference: ↑ higher is better and ↓ lower is better.

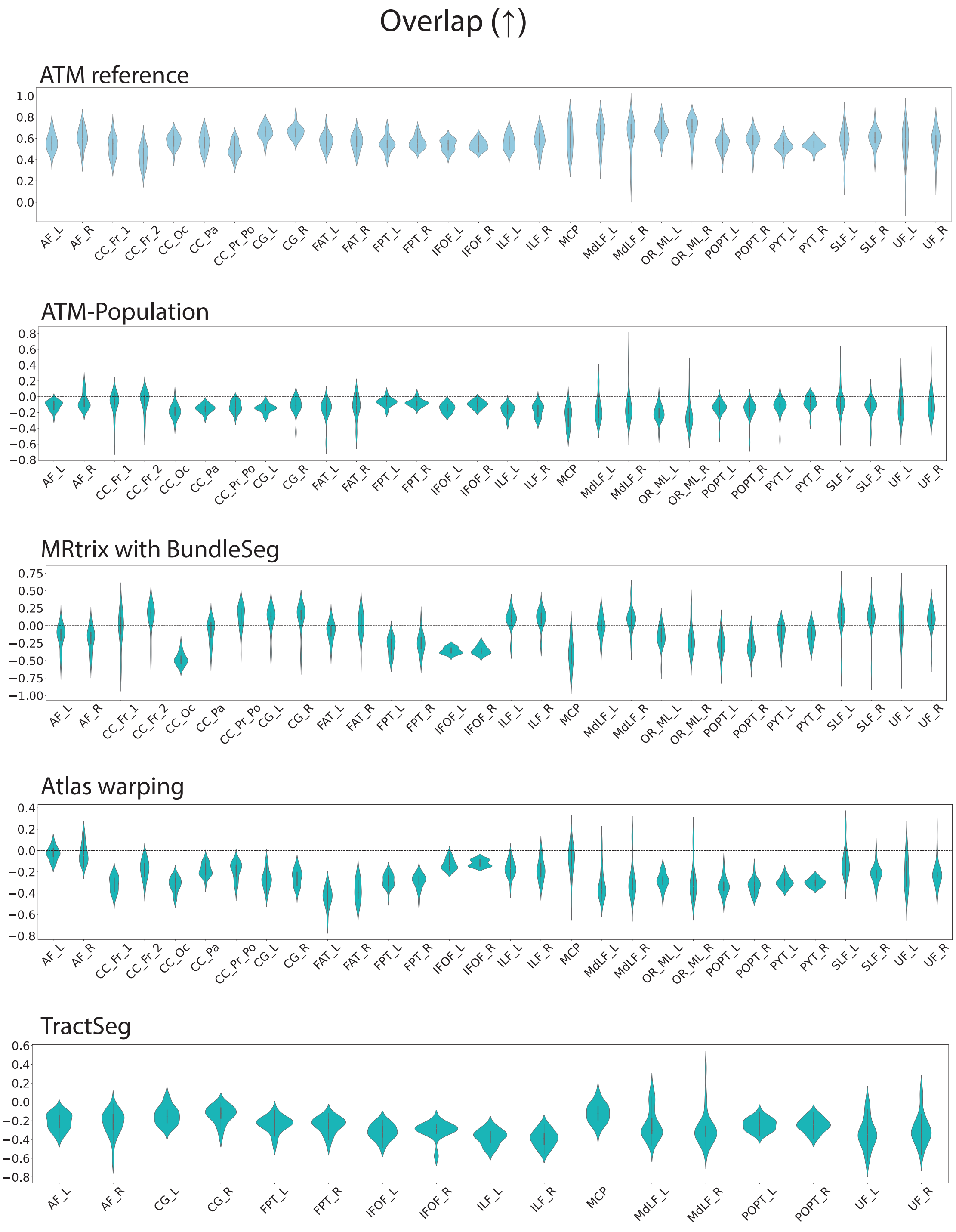

Fig. S16 | Overlap of bundles generated with ATM, ATM-Population, MRtrix, SCIL WM atlas warping, and TractSeg. The first violin plot shows the ATM values as the baseline. The subsequent rows display the paired performance differences of other methods relative to this baseline. For each metric, the arrow indicates preference: ↑ higher is better and ↓ lower is better.

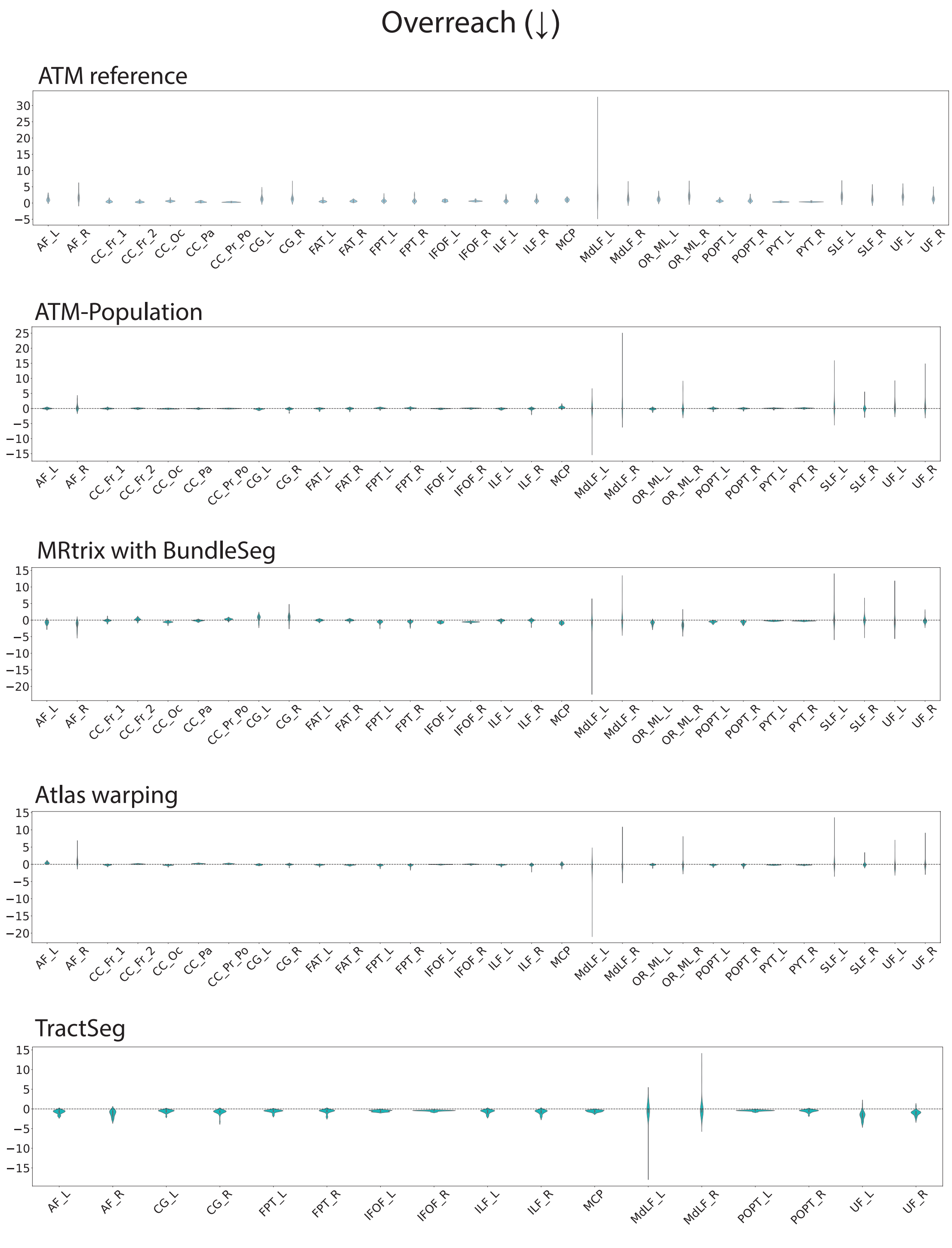

Fig. S17 | Overreach of bundles generated with ATM, ATM-Population, MRtrix with BundleSeg, SCIL WM atlas warping, and TractSeg. The first violin plot shows the ATM values as the baseline. The subsequent rows display the paired performance differences of other methods relative to this baseline. For each metric, the arrow indicates preference: ↑ higher is better and ↓ lower is better.

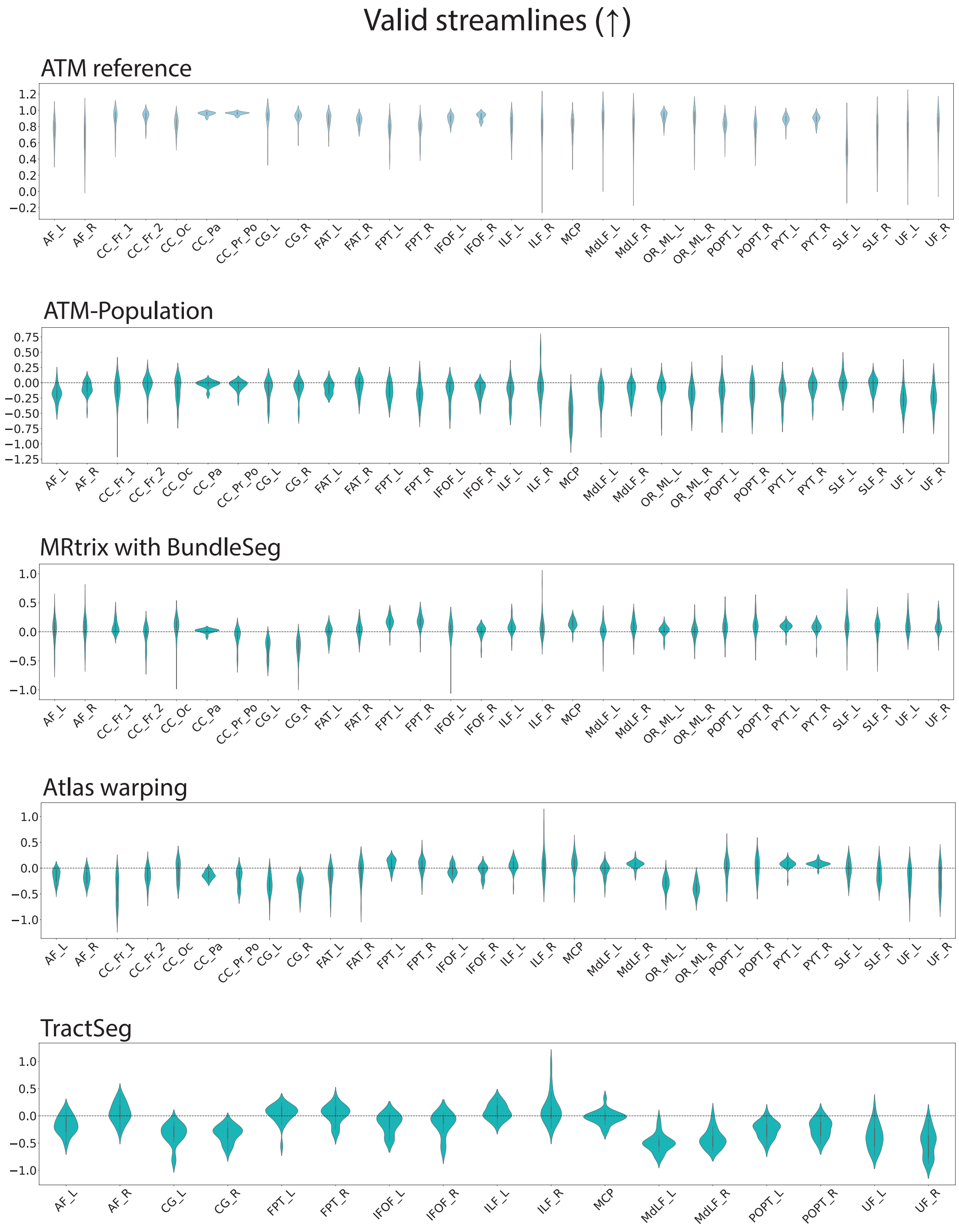

Fig. S18 | Ratio of valid streamlines in bundles generated with ATM, ATM-Population, MRtrix with BundleSeg, SCIL WM atlas warping, and TractSeg. The first violin plot shows the ATM values as the baseline. The subsequent rows display the paired performance differences of other methods relative to this baseline. For each metric, the arrow indicates preference: ↑ higher is better and ↓ lower is better.

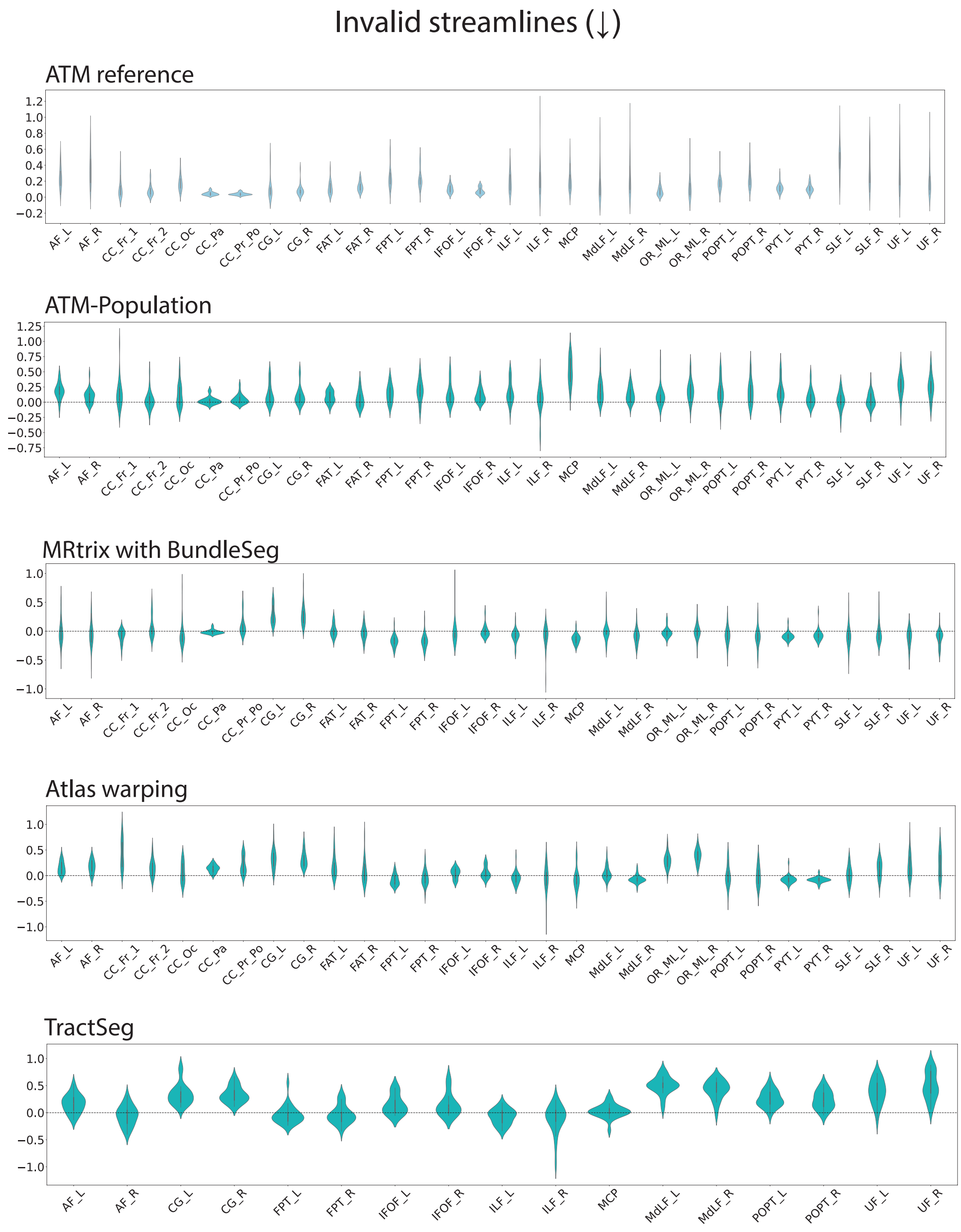

Fig. S19 | Ratio of invalid streamlines in bundles generated with ATM, ATM-Population, MRtrix with BundleSeg, SCIL WM atlas warping, and TractSeg. The first violin plot shows the ATM values as the baseline. The subsequent rows display the paired performance differences of other methods relative to this baseline. For each metric, the arrow indicates preference: ↑ higher is better and ↓ lower is better.

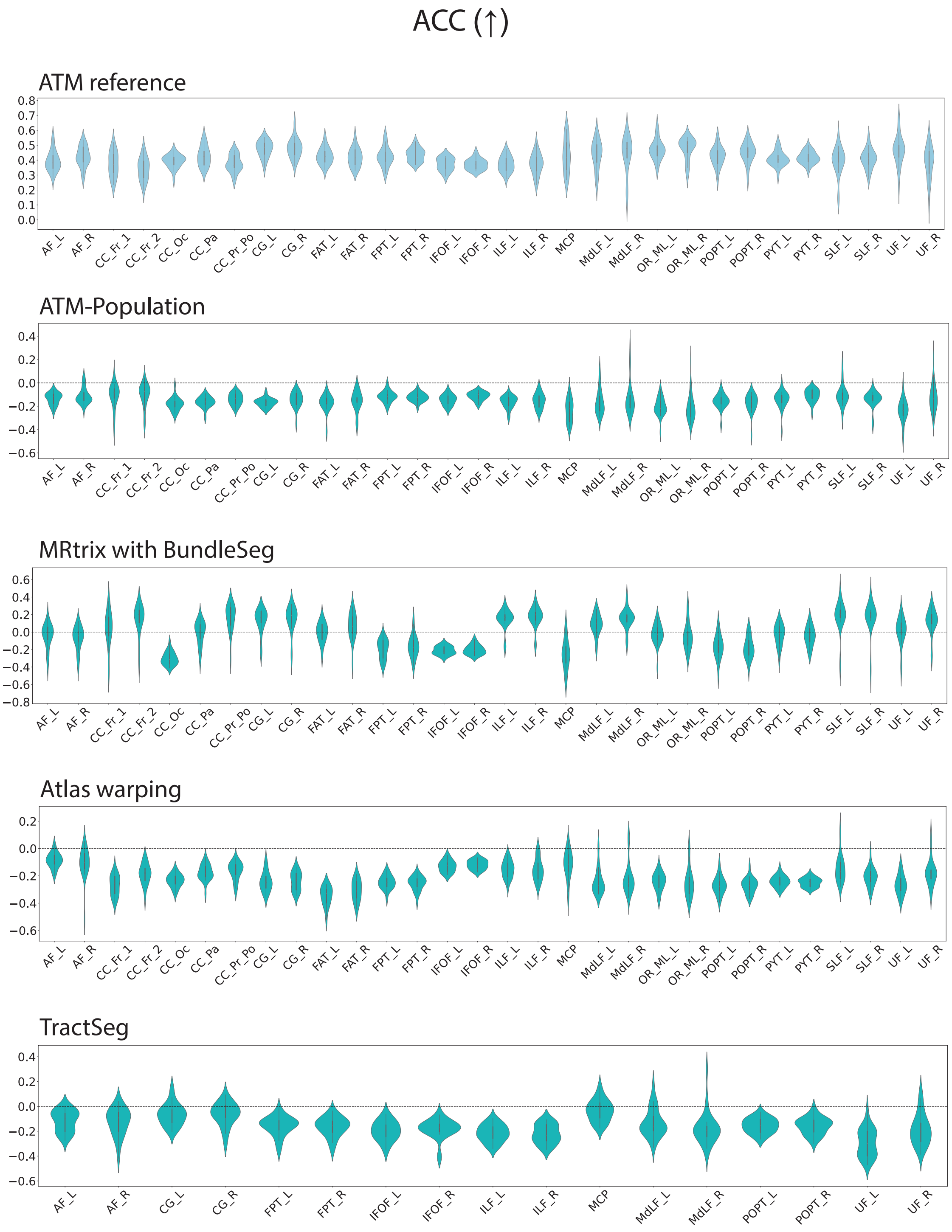

Fig. S20 | Angular correlation coefficient of bundles generated with ATM, ATM-Population, MRtrix with BundleSeg, SCIL WM atlas warping, and TractSeg. The first violin plot shows the ATM values as the baseline. The subsequent rows display the paired performance differences of other methods relative to this baseline. For each metric, the arrow indicates preference: ↑ higher is better and ↓ lower is better.

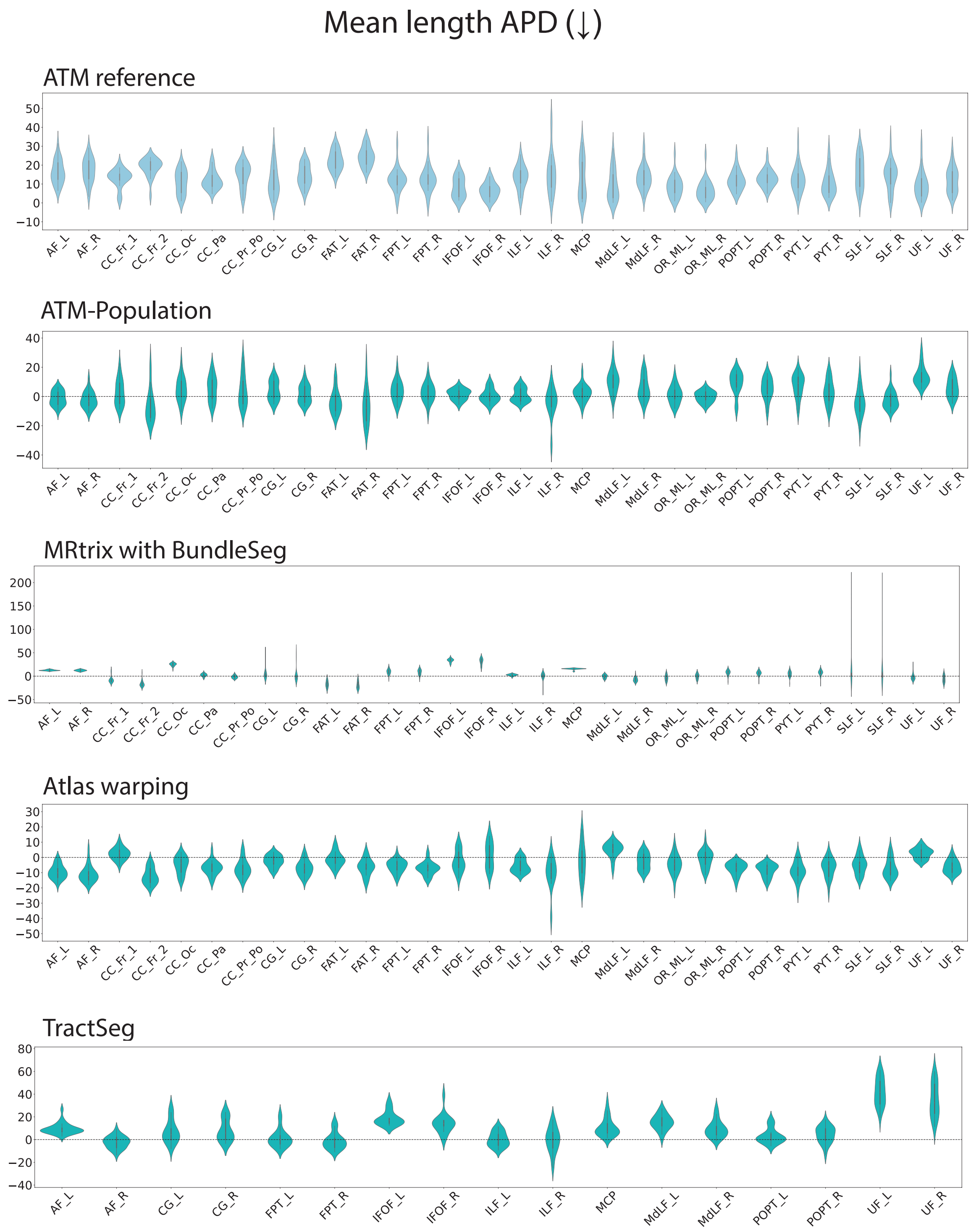

Fig. S21 | Mean length APD of bundles generated with ATM, ATM-Population, MRtrix with BundleSeg, SCIL WM atlas warping, and TractSeg. The first violin plot shows the ATM values as the baseline. The subsequent rows display the paired performance differences of other methods relative to this baseline. For each metric, the arrow indicates preference: ↑ higher is better and ↓ lower is better.

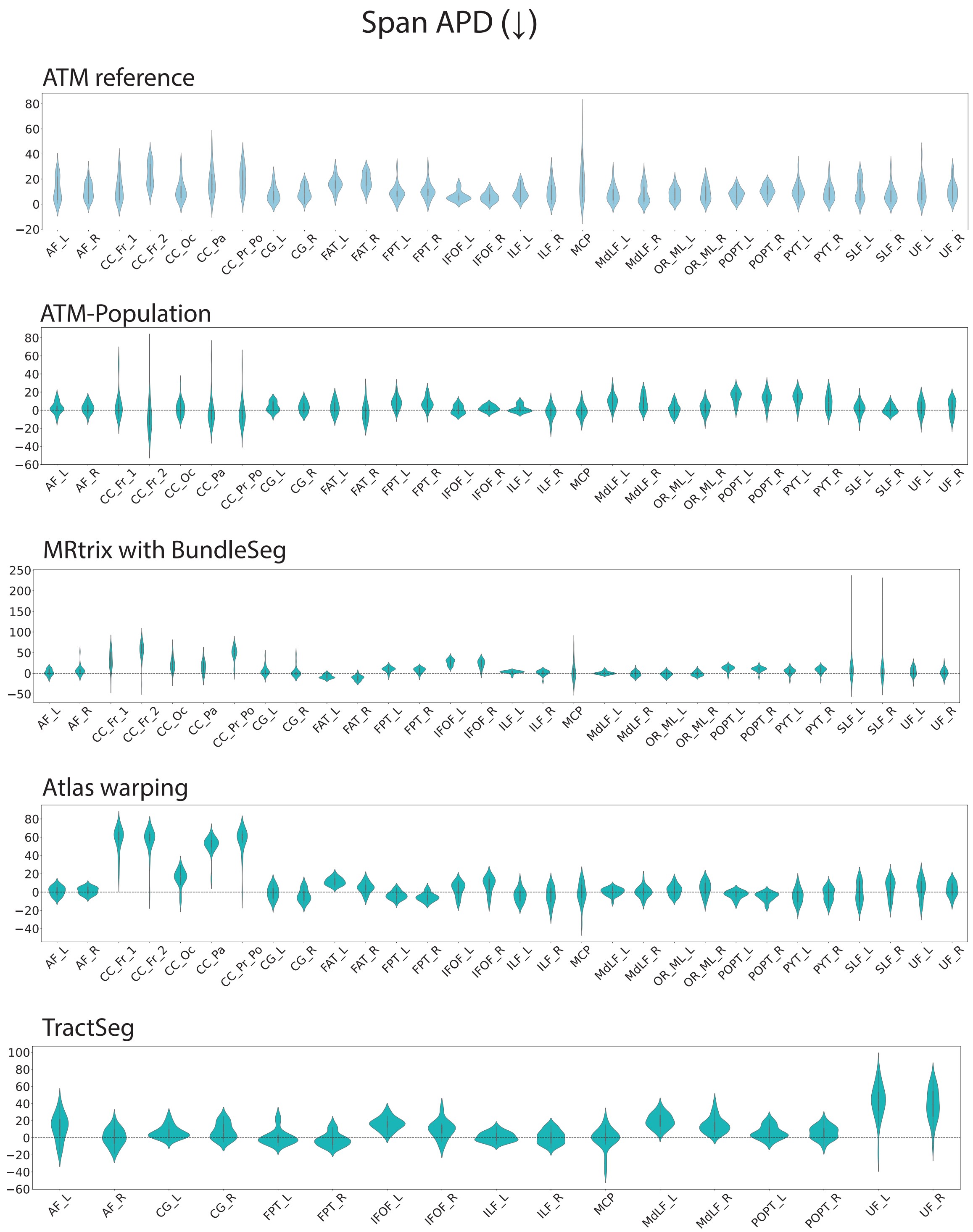

Fig. S22 | Span APD of bundles generated with ATM, ATM-Population, MRtrix with BundleSeg, SCIL WM atlas warping, and TractSeg. The first violin plot shows the ATM values as the baseline. The subsequent rows display the paired performance differences of other methods relative to this baseline. For each metric, the arrow indicates preference: ↑ higher is better and ↓ lower is better.

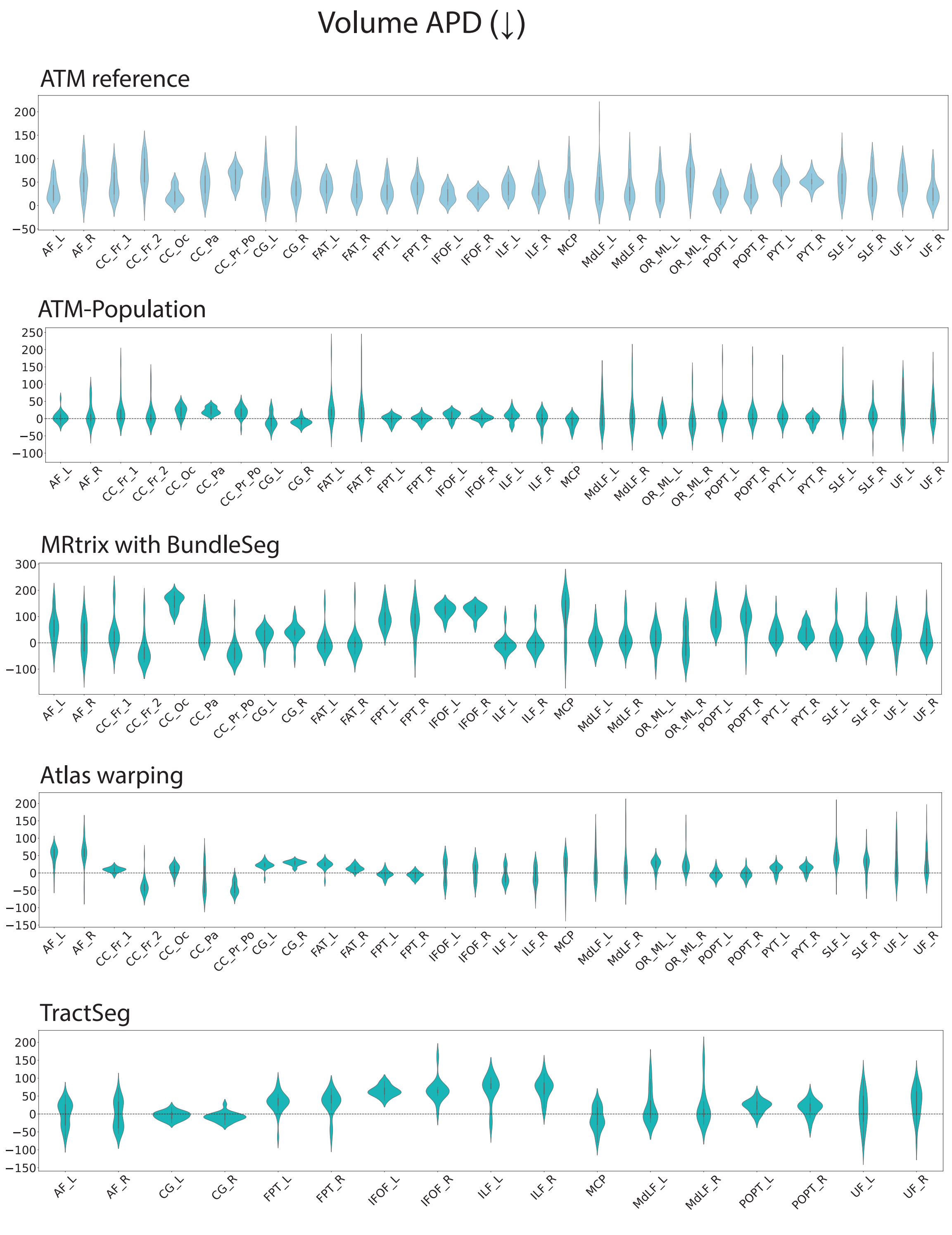

Fig. S23 | Volume APD of bundles generated with ATM, ATM-Population, MRtrix with BundleSeg, SCIL WM atlas warping, and TractSeg. The first violin plot shows the ATM values as the baseline. The subsequent rows display the paired performance differences of other methods relative to this baseline. For each metric, the arrow indicates preference: ↑ higher is better and ↓ lower is better.

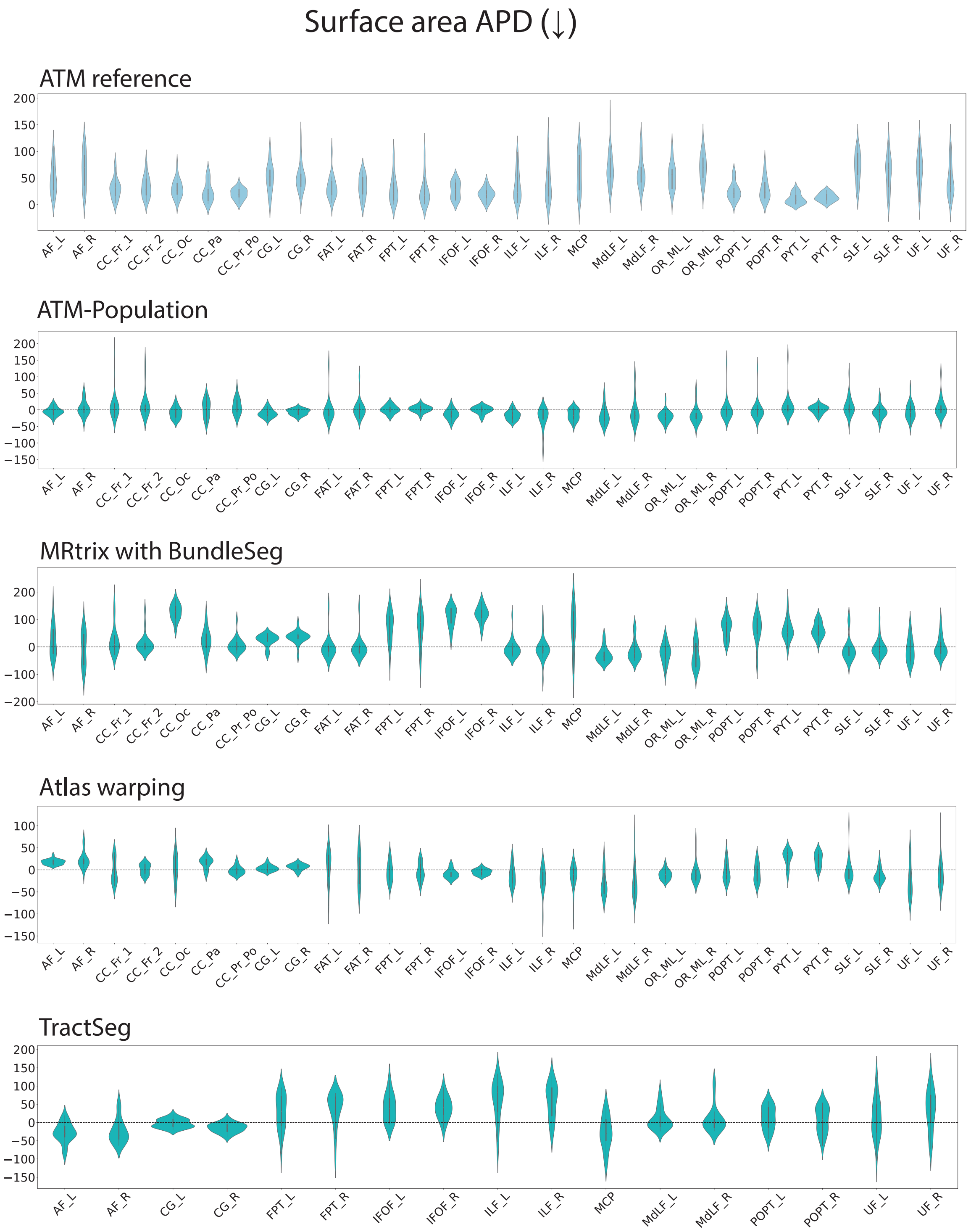

Fig. S24 | Surface area APD of bundles generated with ATM, ATM-Population, MRtrix with BundleSeg, SCIL WM atlas warping, and TractSeg. The first violin plot shows the ATM values as the baseline. The subsequent rows display the paired performance differences of other methods relative to this baseline. For each metric, the arrow indicates preference: ↑ higher is better and ↓ lower is better.

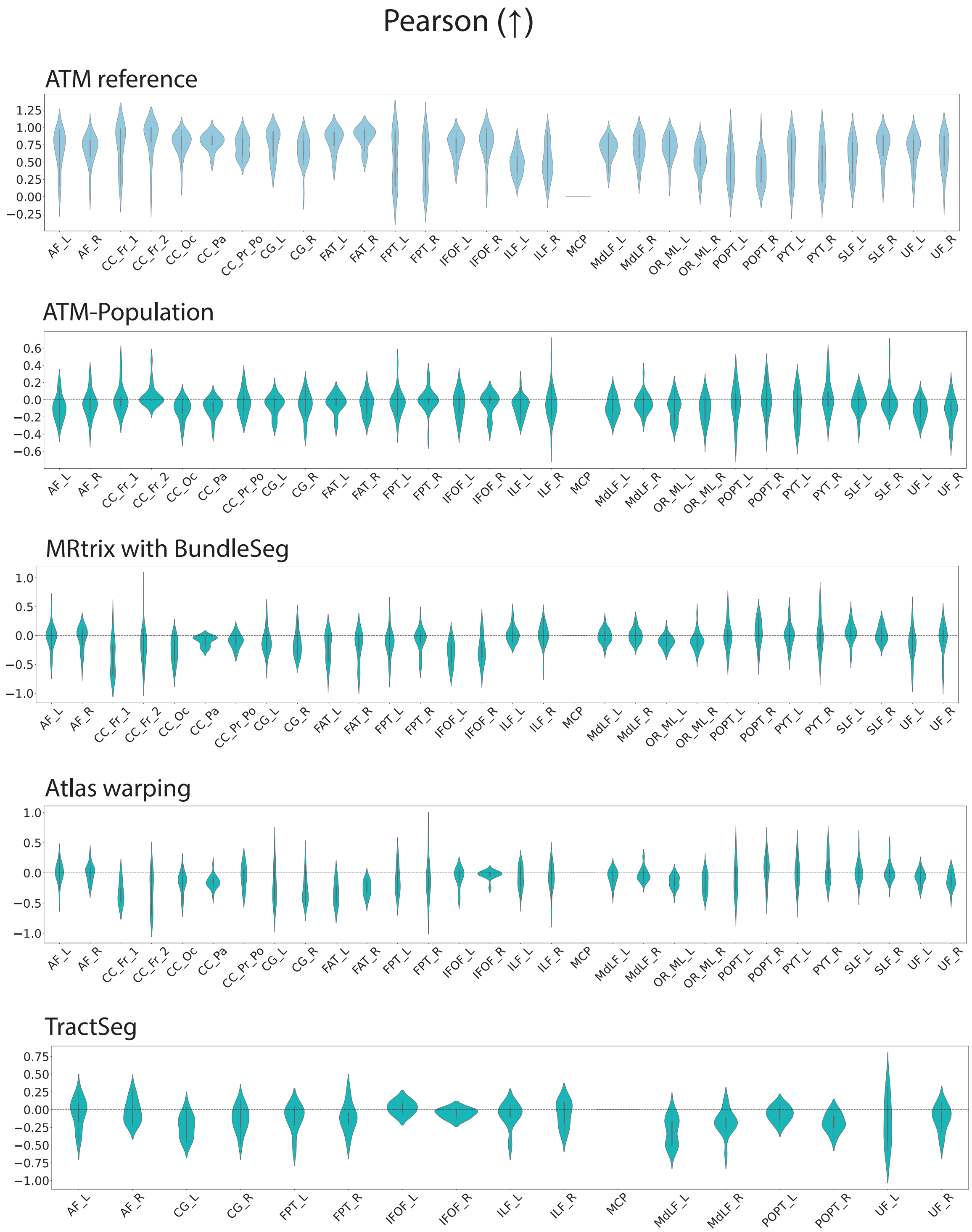

Fig. S25 | Pearson corerlation of bundle connectomes generated with ATM, ATM-Population, MRtrix with BundleSeg, SCIL WM atlas warping, and TractSeg. The first violin plot shows the ATM values as the baseline. The subsequent rows display the paired performance differences of other methods relative to this baseline. For each metric, the arrow indicates preference: ↑ higher is better and ↓ lower is better.

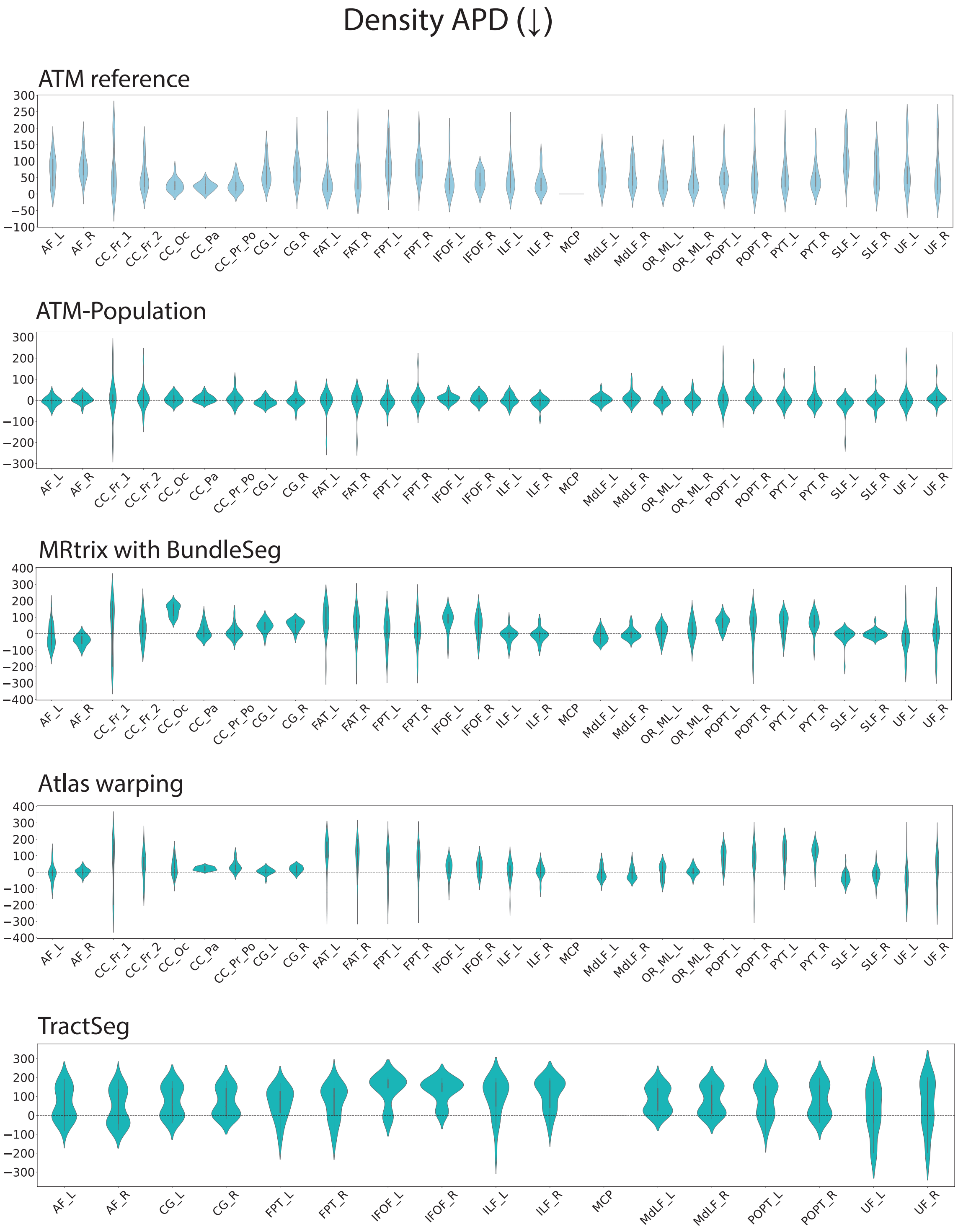

Fig. S26 | Network density APD of bundle connectomes generated with ATM, ATM-Population, MRtrix with BundleSeg, SCIL WM atlas warping, and TractSeg. The first violin plot shows the ATM values as the baseline. The subsequent rows display the paired performance differences of other methods relative to this baseline. For each metric, the arrow indicates preference: ↑ higher is better and ↓ lower is better.

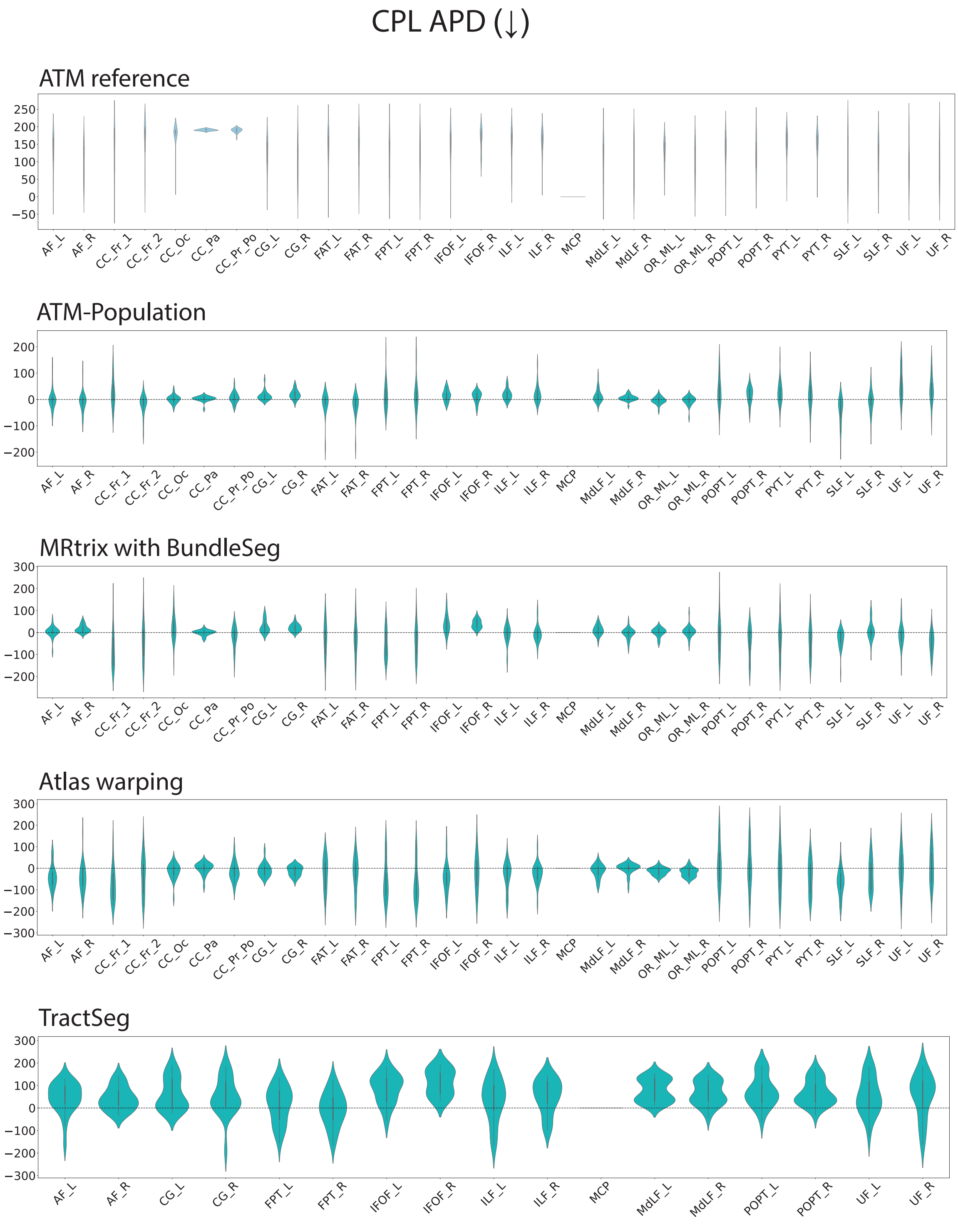

Fig. S27 | Network CPL APD of bundle connectomes generated with ATM, ATM-Population, MRtrix with BundleSeg, SCIL WM atlas warping, and TractSeg. The first violin plot shows the ATM values as the baseline. The subsequent rows display the paired performance differences of other methods relative to this baseline. For each metric, the arrow indicates preference: ↑ higher is better and ↓ lower is better.

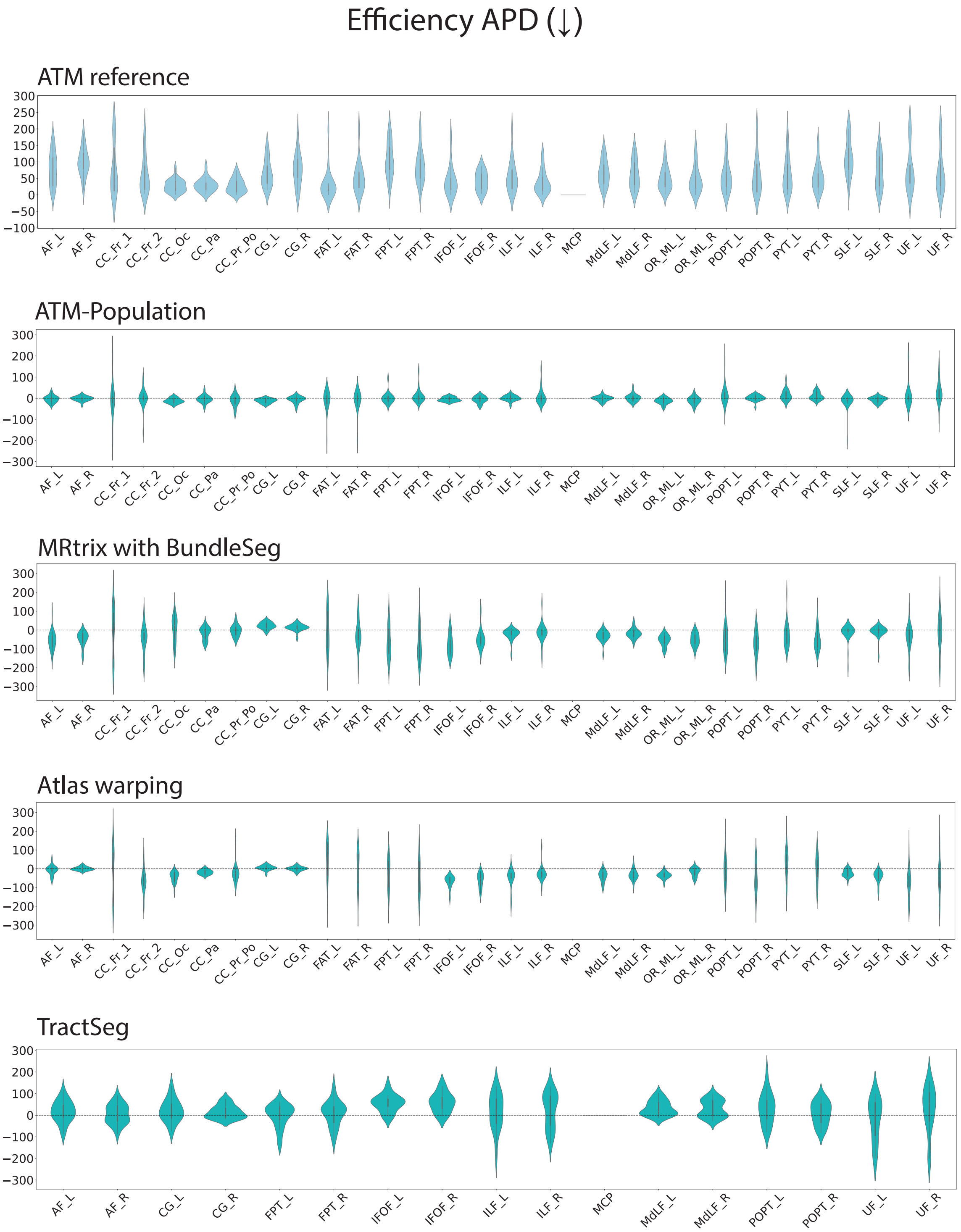

Fig. S28 | Network global efficiency APD of bundle connectomes generated with ATM, ATM-Population, MRtrix with BundleSeg, SCIL WM atlas warping, and TractSeg. The first violin plot shows the ATM values as the baseline. The subsequent rows display the paired performance differences of other methods relative to this baseline. For each metric, the arrow indicates preference: ↑ higher is better and ↓ lower is better.

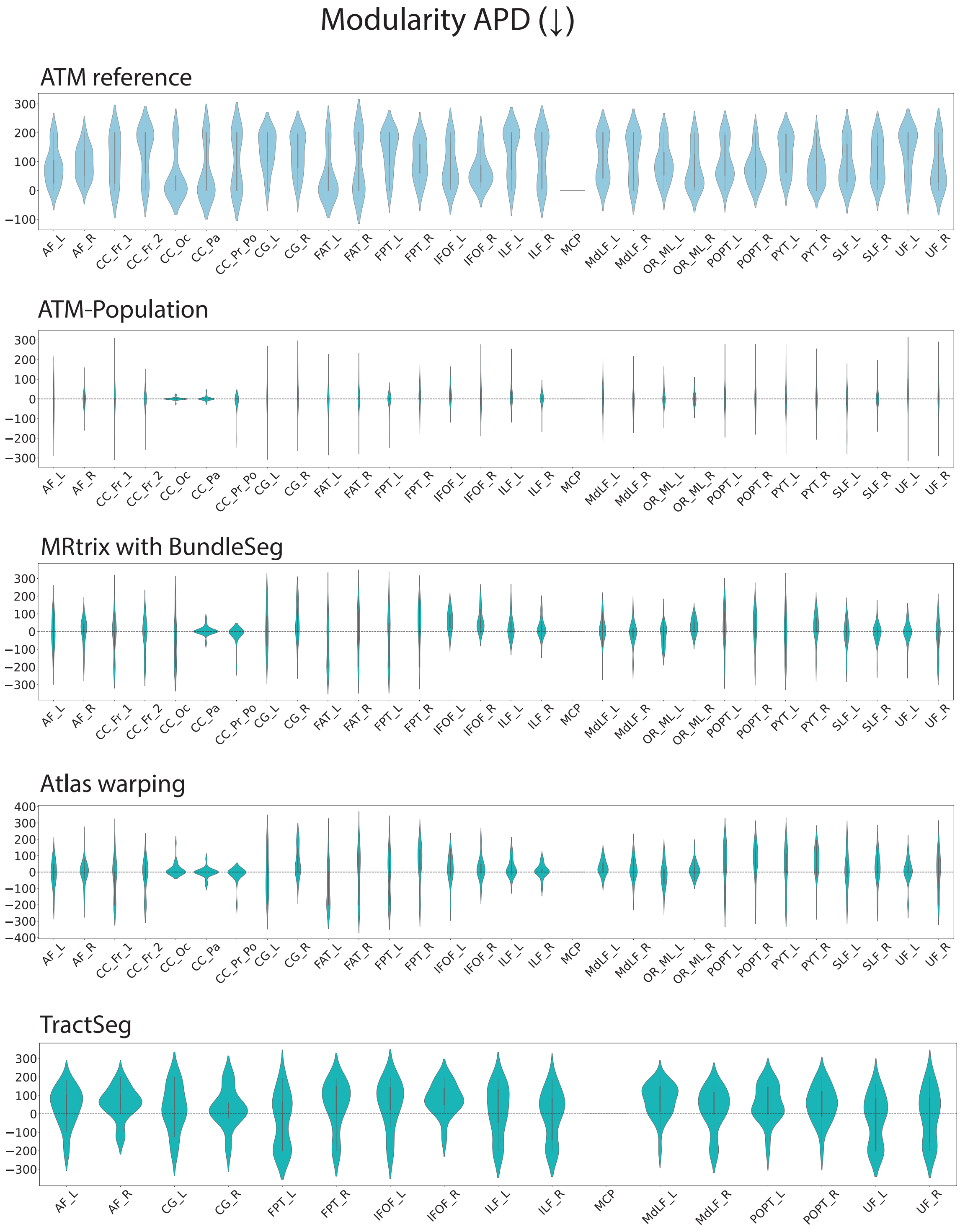

Fig. S29 | Network modularity APD of bundle connectomes generated with ATM, ATM-Population, MRtrix with BundleSeg, SCIL WM atlas warping, and TractSeg. The first violin plot shows the ATM values as the baseline. The subsequent rows display the paired performance differences of other methods relative to this baseline. For each metric, the arrow indicates preference: ↑ higher is better and ↓ lower is better.
