# Supplementary material for: Anatomy-to-Tract Mapping: Inferring White Matter Pathways Without Diffusion Streamline Propagation
Source: bioRxiv. 2025 Jul 16:2024.12.24.630267. Preprint. [Version 2] doi: 10.1101/2024.12.24.630267 (PMC12338524; doi:10.1101/2024.12.24.630267)
Supplement: Supplementary file 1 [file media-1.pdf]

Supplementary Note: Architectural Details of ATM

The segmentation module of ATM comprises a total of C 3D convolutional downsampling blocks, followed by $C-1$ upsampling blocks. The entire module is complemented by a long-skip connection for precise bundle segmentation. These convolutional blocks employ a kernel size of 3, and each block is equipped with filters specified as {32, 64, 128, 256}, followed by upsampling blocks with filter sizes of {128, 64, 32}. The final upsampling block is followed by a 3D convolutional output layer with a filter size of 1. The encoder-decoder component of the streamline VAE module is structured with C 1D convolutional downsampling and upsampling blocks. This module utilizes a kernel size of 63, a latent space dimensionality of 64, and filter size for each downsampling and upsampling block are set as {32, 64, 128, 256} and {256, 128, 64, 32}, respectively. Each upsampling block is coupled with a FiLM layer[52] for adaptive feature modulation except the output layer with a filter size of 3. These FiLM layers receive input from the FiLM shared-weight block, which consists of two layers of multi-layer perceptron (MLP).

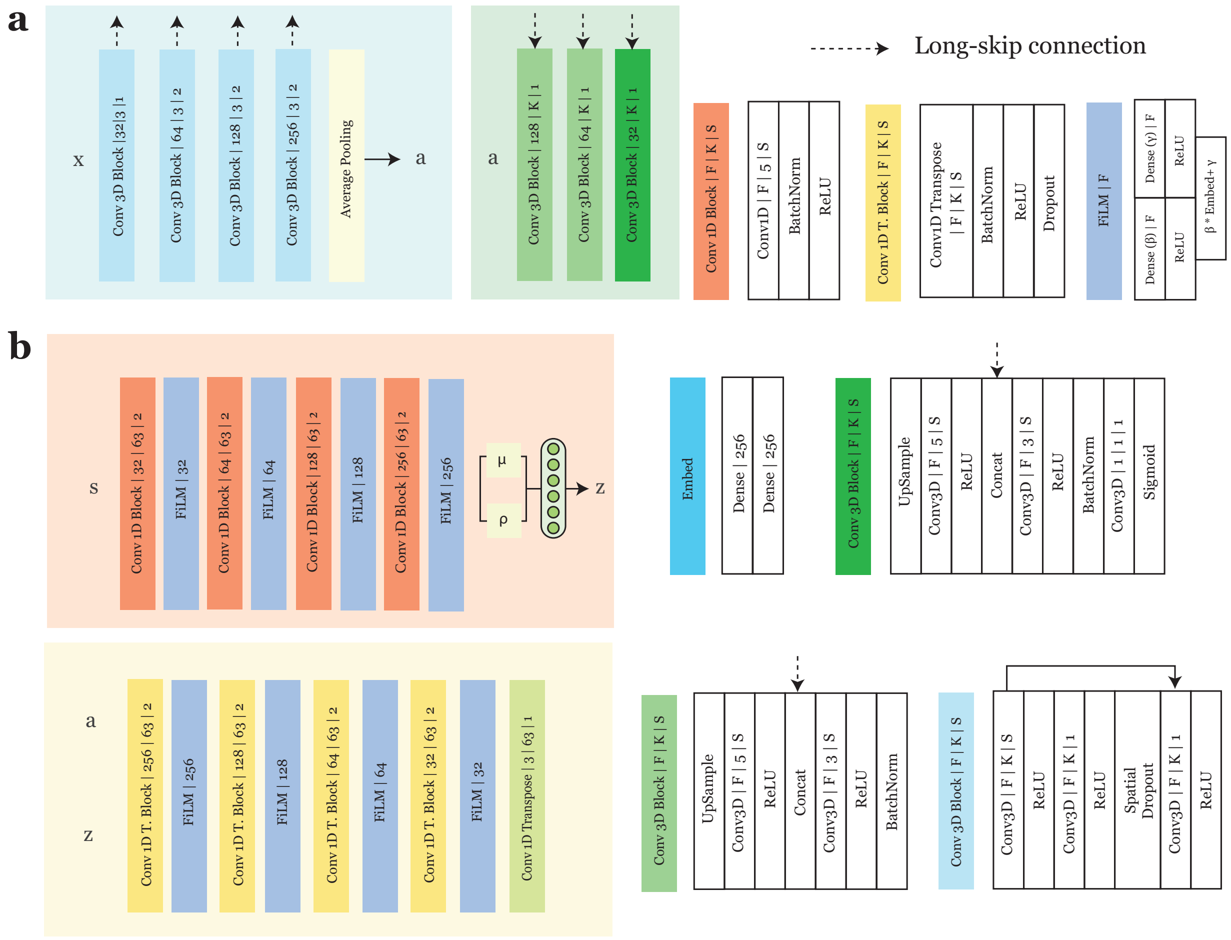

Fig. S1 | **ATM architectural details**. **a**, Network architecture of T1w encoder, E_A, and decoder, D_A. **b**, Network architectures of streamline encoder, E_S, and decoder, D_S.

Tab. S1 | White matter bundles.

**Bundle**	**Abbreviation**
Arcuate fascicle	AF
Corpus callosum, Frontal lobe (most anterior part)	CC_Fr_1
Corpus callosum, Frontal lobe (most anterior part)	CC_Fr_2
Corpus callosum, Occipital lobe	CC_Oc
Corpus callosum, Parietal lobe	CC_Pa
Corpus callosum, Pre/Post central gyri	CC_Pr_Po
Cingulum	CG
Frontal aslant tract	FAT
Fronto-pontine tract	FPT
Inferior fronto-occipital fasciculus	IFOF
Inferior longitudinal fasciculus	ILF
Middle cerebellar peduncle	MCP
Middle longitudinal fascicle	MdLF
Optic radiation and Meyer's loop	OR_ML
Parieto-occipito pontine tract	POPT
Pyramidal tract	PYT
Superior longitudinal fasciculus	SLF
Uncinate fasciculus	UF

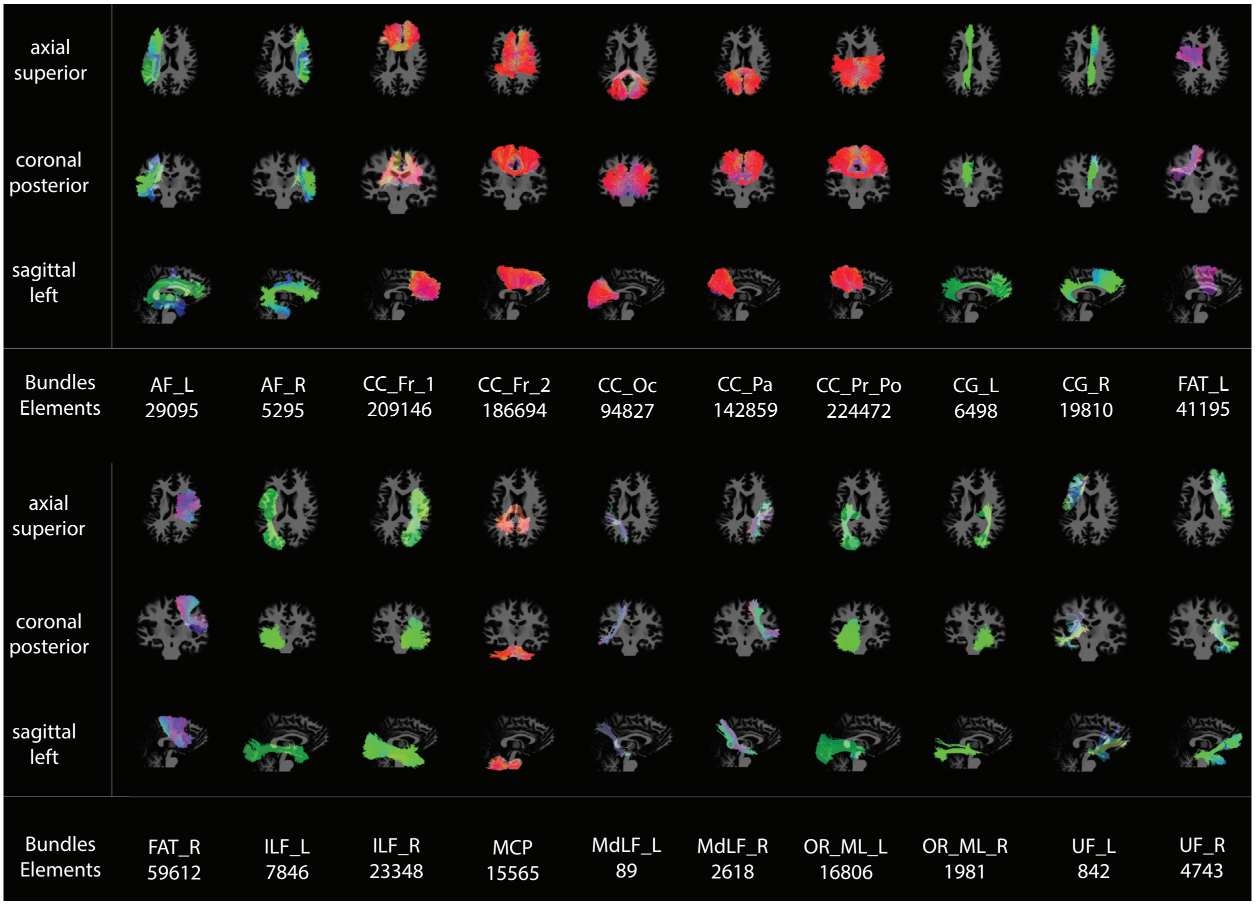

Fig. S2 | **Bundle comparison.** Ground truth bundles from Tractoinferno for a single representative subject.

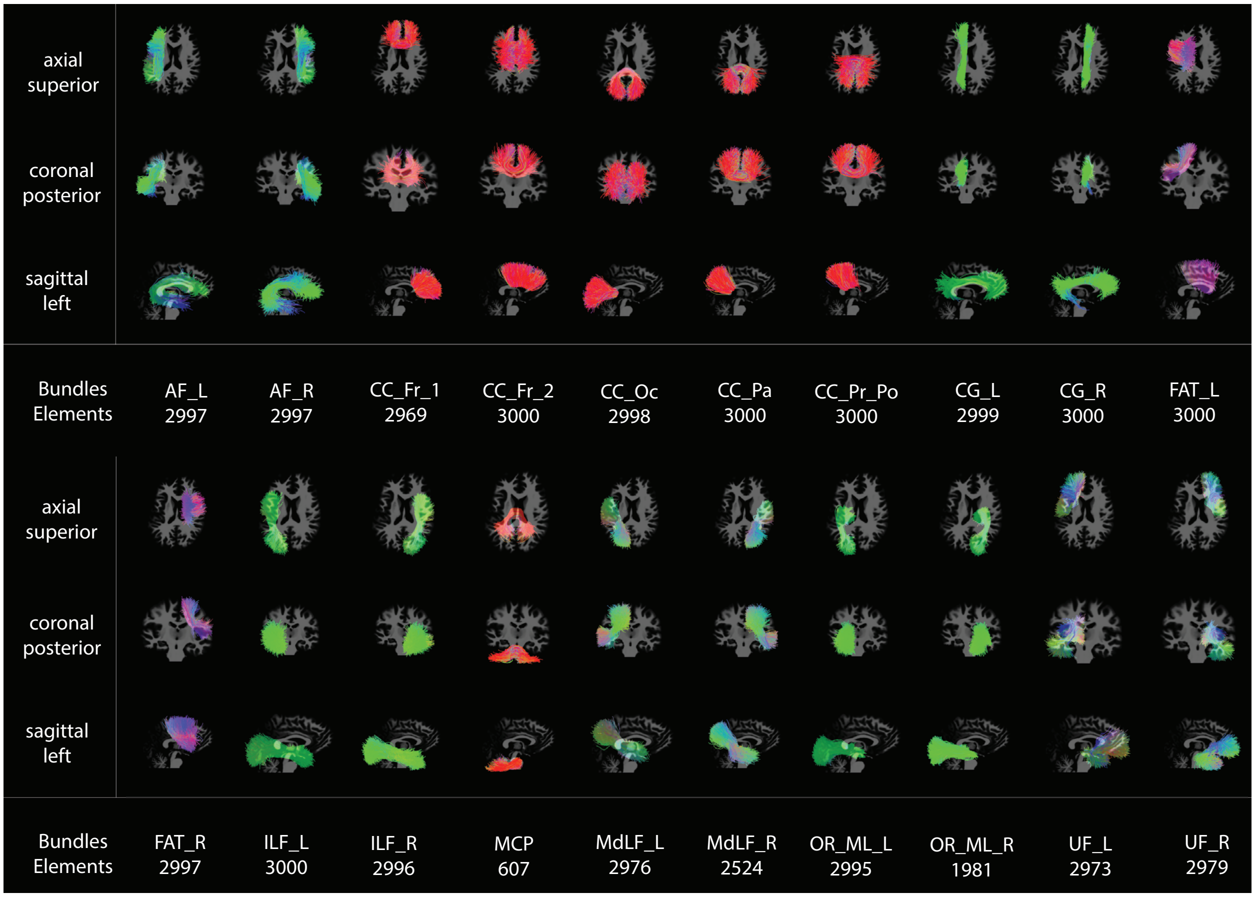

Fig. S3 | **Bundle comparison.** Bundles generated with ATM for a single representative subject.

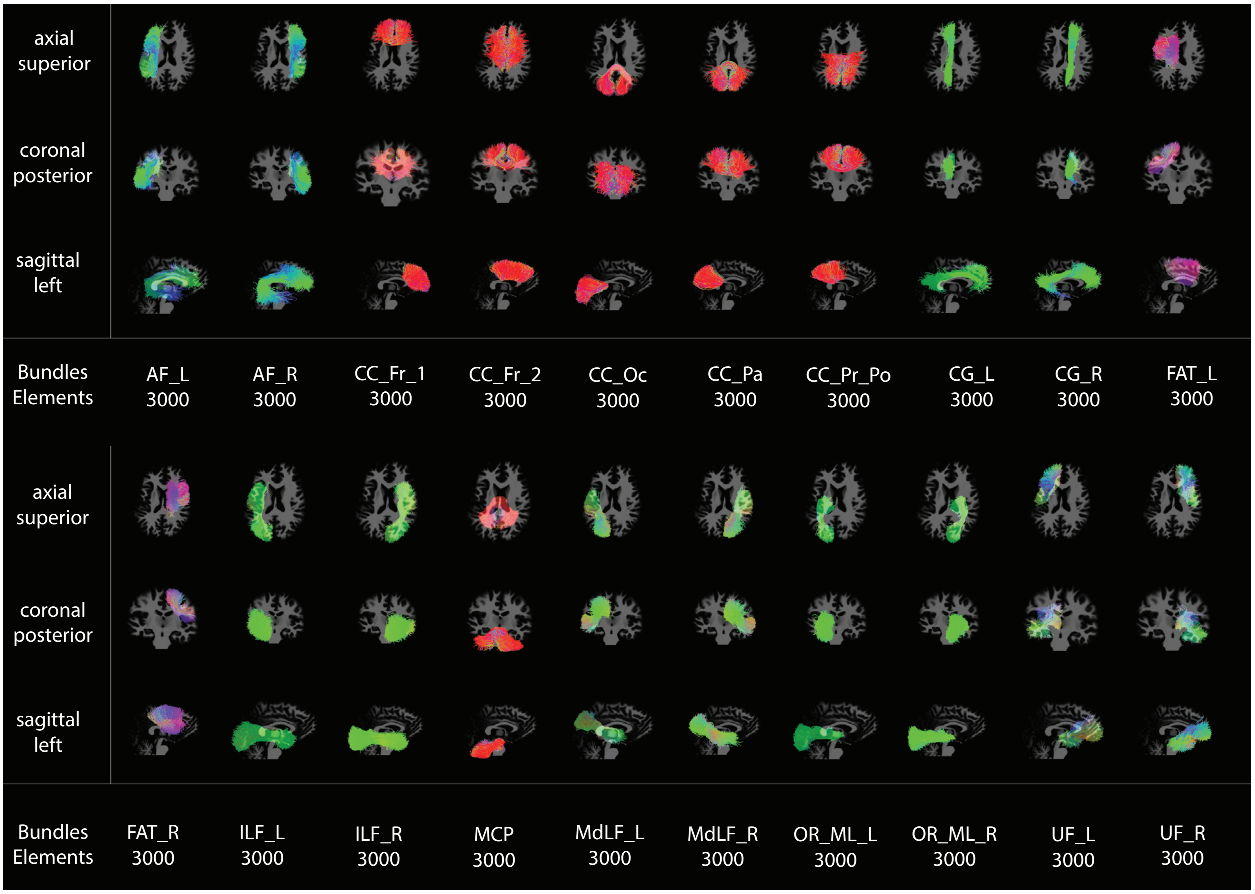

Fig. S4 | Bundle comparison. Bundles generated with ATM Population for a single representative subject.

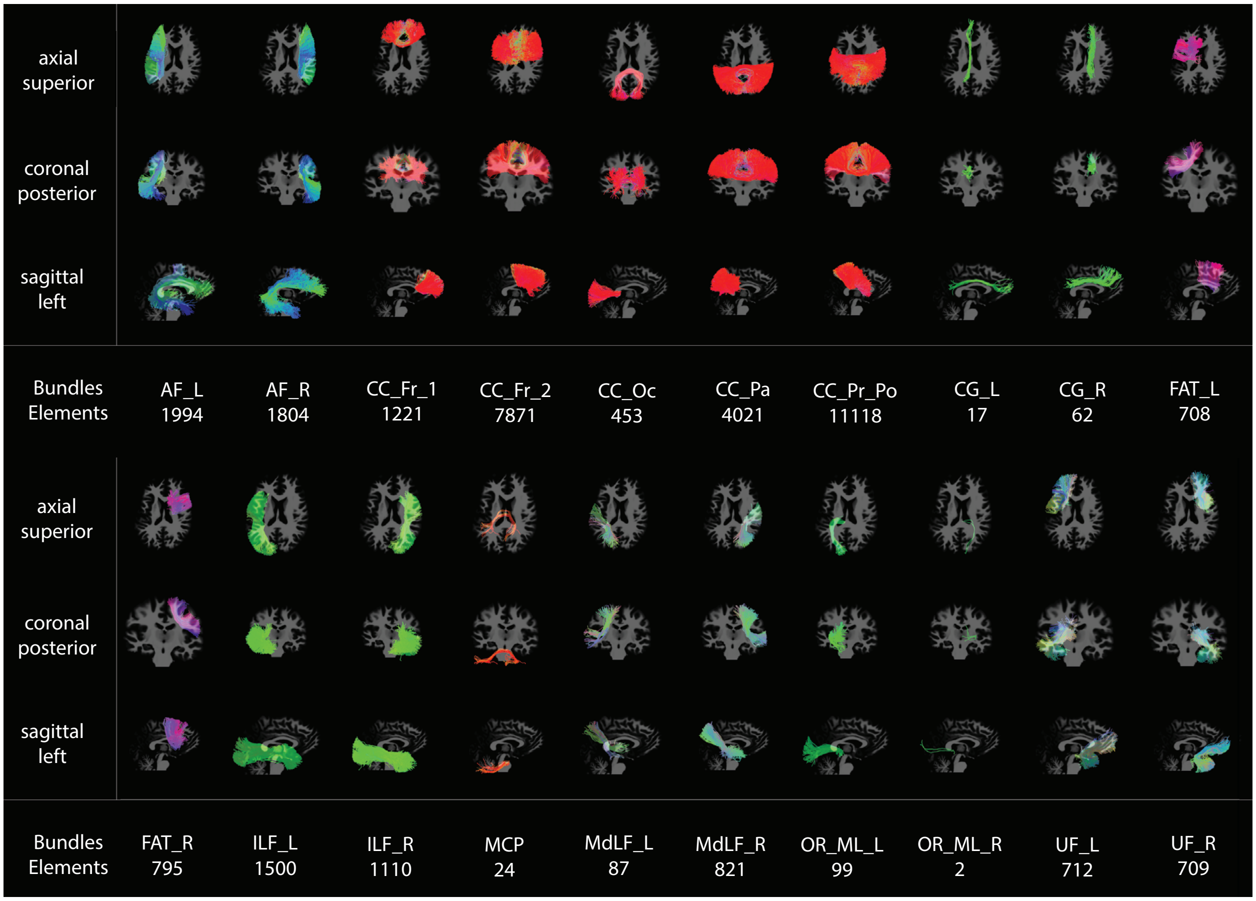

Fig. S5 | **Bundle comparison.** Bundles generated with MRtrix for a single representative subject.

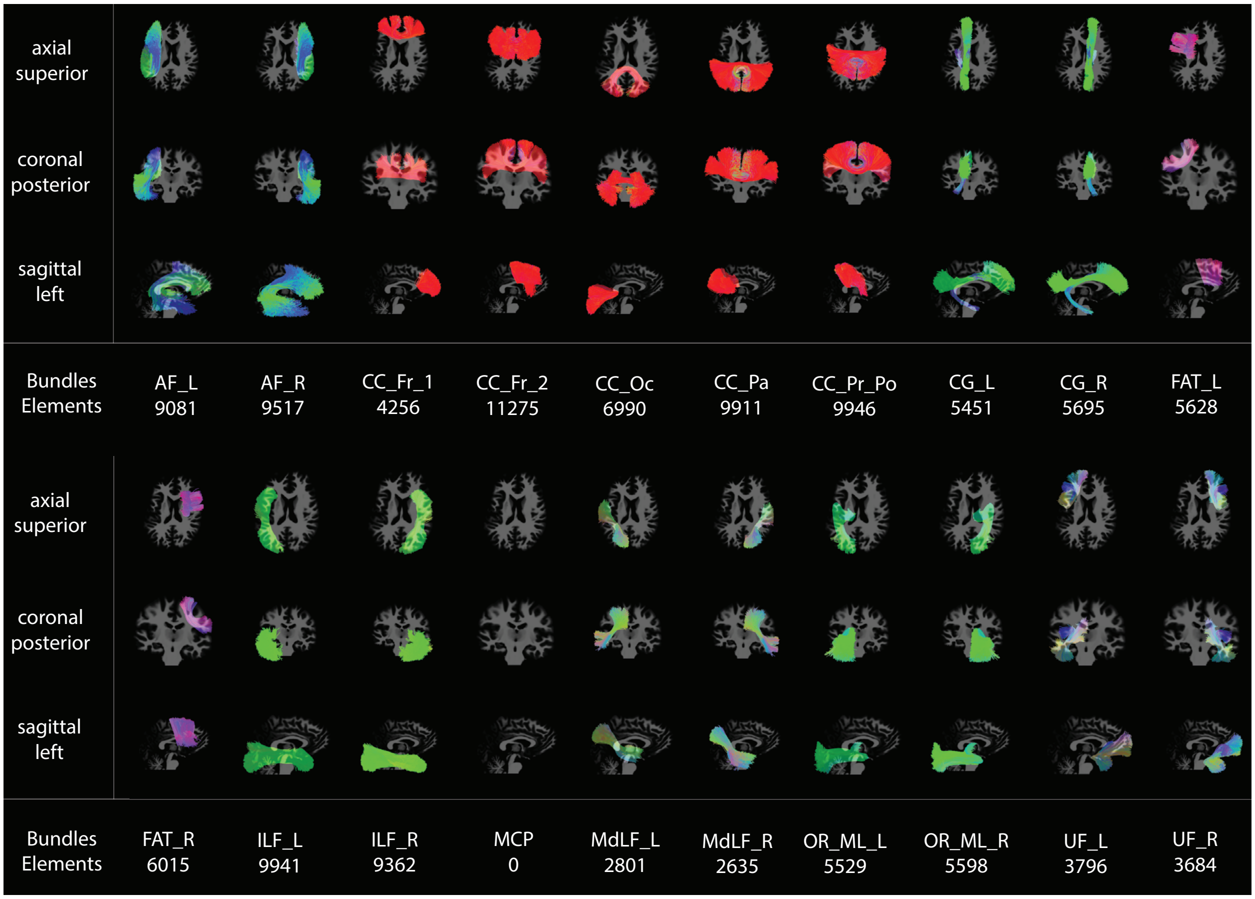

Fig. S6 | **Bundle comparison.** Bundles generated with RecobundlesX atlas warping for a single representative subject.

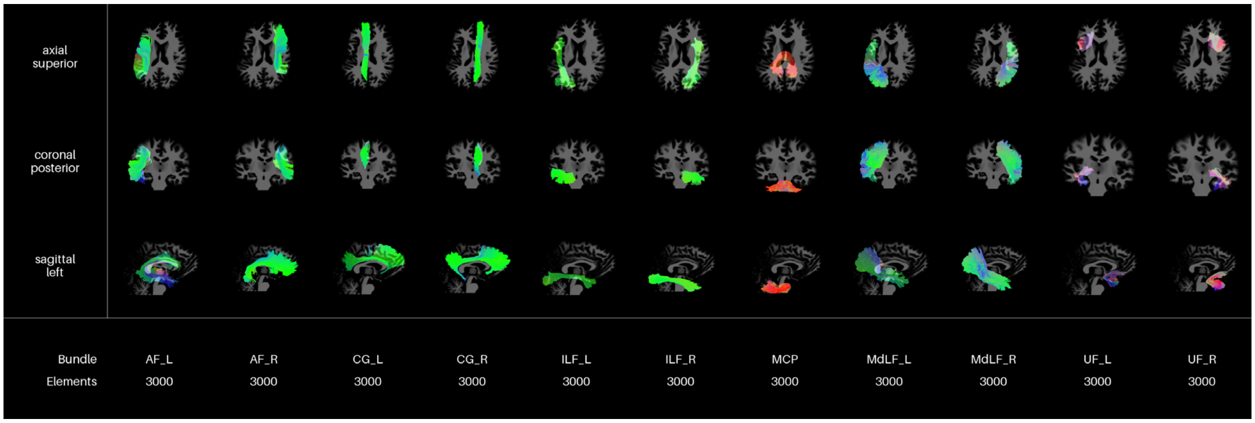

Fig. S7 | **Bundle comparison.** Bundles generated with TractSeg for a single representative subject.

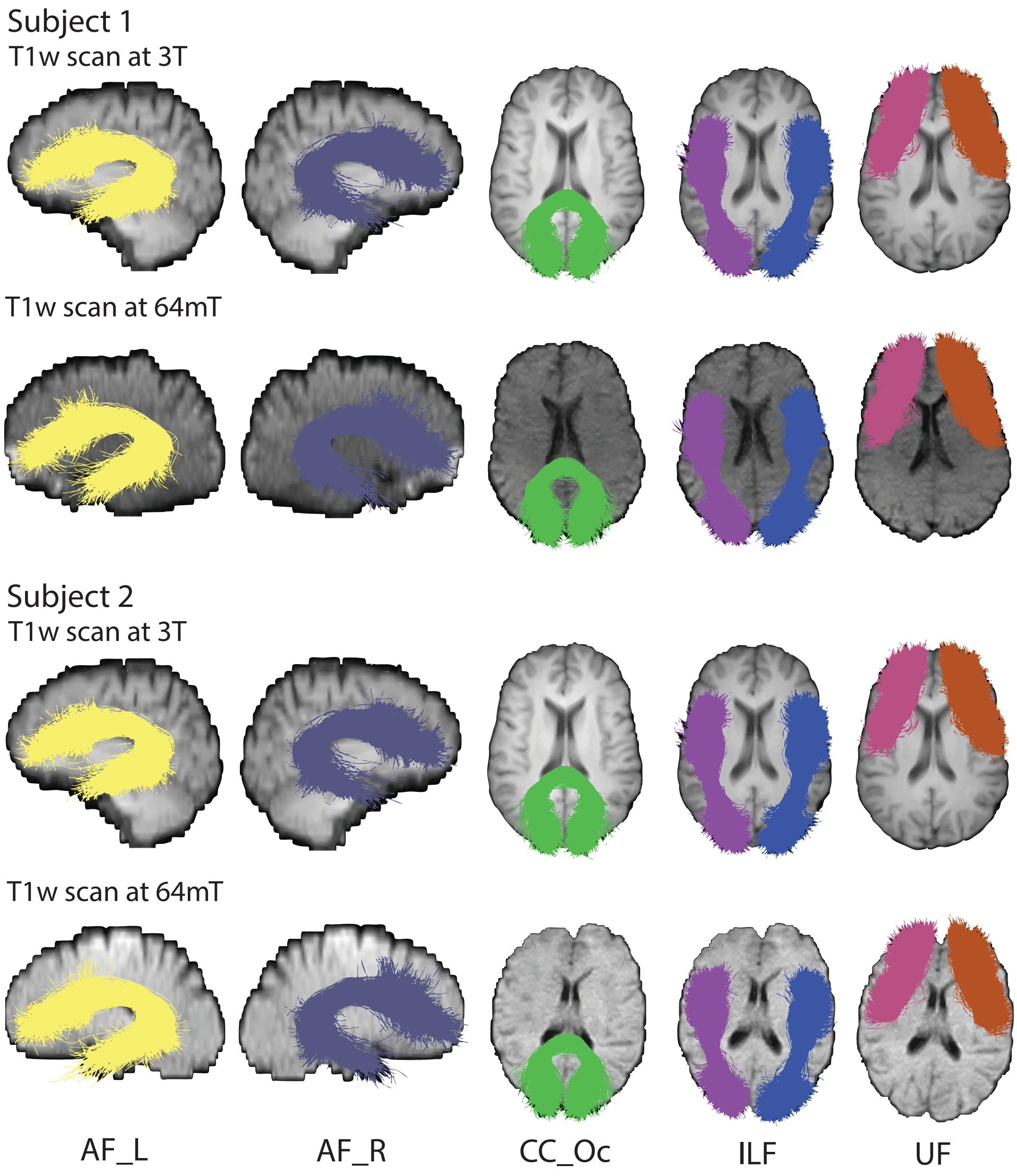

Fig. S8 | **Clinical scans.** Bundles generated with ATM for clincal T1w scans acquired at 3T with 1.5 mm × 1.5 mm × 5 mm resolution and at 64mT with 1.6 mm × 1.6 mm × 5 mm resolution.

Tab. S2 | Similarity, coverage, and validity of individual bundles generated with ATM. Values are presented as mean ± standard deviation. For each metric, the arrow indicates preference: ↑ higher is better, ↓ lower is better.

Bundles	BA (↑)	Coverage			Validity		ACC (↑)
		Dice (↑)	Overlap (↑)	Overreach (↓)	VS (↑)	IS (↓)	
AF_L	0.7365 ± 0.1000	0.4149 ± 0.0540	0.5621 ± 0.0817	1.2206 ± 0.6170	0.7524 ± 0.1310	0.2476 ± 0.1310	0.3934 ± 0.0665
AF_R	0.6876 ± 0.1392	0.3607 ± 0.0878	0.6036 ± 0.0939	2.0266 ± 1.2352	0.6403 ± 0.2039	0.3597 ± 0.2039	0.4239 ± 0.0667
CC_Fr_1	0.8637 ± 0.1135	0.5175 ± 0.0585	0.5203 ± 0.0983	0.4976 ± 0.2881	0.8855 ± 0.1199	0.1145 ± 0.1199	0.3742 ± 0.0819
CC_Fr_2	0.8498 ± 0.0653	0.4857 ± 0.0509	0.4369 ± 0.0965	0.3497 ± 0.2158	0.9158 ± 0.0717	0.0842 ± 0.0717	0.3418 ± 0.0737
CC_Oc	0.8516 ± 0.0663	0.5287 ± 0.0489	0.5758 ± 0.0635	0.6150 ± 0.2440	0.8278 ± 0.0885	0.1722 ± 0.0885	0.3932 ± 0.0442
CC_Pa	0.9420 ± 0.0178	0.5848 ± 0.0281	0.5633 ± 0.0830	0.3592 ± 0.1676	0.9567 ± 0.0216	0.0433 ± 0.0216	0.4269 ± 0.0658
CC_Pr_Po	0.9355 ± 0.0341	0.5560 ± 0.0428	0.4958 ± 0.0727	0.2805 ± 0.0942	0.9615 ± 0.0162	0.0385 ± 0.0162	0.3774 ± 0.0560
CG_L	0.8914 ± 0.0910	0.4382 ± 0.0831	0.6571 ± 0.0639	1.5003 ± 0.8744	0.8824 ± 0.1431	0.1176 ± 0.1431	0.4751 ± 0.0543
CG_R	0.9055 ± 0.0552	0.4331 ± 0.0765	0.6610 ± 0.0592	1.5706 ± 1.0833	0.9123 ± 0.0717	0.0877 ± 0.0717	0.4788 ± 0.0645
FAT_L	0.8702 ± 0.0627	0.5504 ± 0.0448	0.5753 ± 0.0711	0.5303 ± 0.2840	0.8826 ± 0.0815	0.1174 ± 0.0815	0.4272 ± 0.0533
FAT_R	0.8480 ± 0.0516	0.5239 ± 0.0432	0.5634 ± 0.0750	0.5941 ± 0.2330	0.8675 ± 0.0587	0.1325 ± 0.0587	0.4184 ± 0.0599
FPT_L	0.7563 ± 0.1323	0.4870 ± 0.0659	0.5472 ± 0.0730	0.7568 ± 0.4906	0.7511 ± 0.1355	0.2489 ± 0.1355	0.4240 ± 0.0639
FPT_R	0.7769 ± 0.1017	0.4946 ± 0.0761	0.5611 ± 0.0575	0.8062 ± 0.6539	0.7650 ± 0.1150	0.2350 ± 0.1150	0.4354 ± 0.0442
IFOF_L	0.8548 ± 0.0552	0.4832 ± 0.0316	0.5364 ± 0.0530	0.6971 ± 0.2514	0.8963 ± 0.0486	0.1037 ± 0.0486	0.3730 ± 0.0422
IFOF_R	0.8811 ± 0.0411	0.5004 ± 0.0236	0.5386 ± 0.0454	0.6181 ± 0.1696	0.9217 ± 0.0406	0.0783 ± 0.0406	0.3720 ± 0.0347
ILF_L	0.7876 ± 0.1054	0.4885 ± 0.0686	0.5646 ± 0.0707	0.8207 ± 0.5547	0.8086 ± 0.1227	0.1914 ± 0.1227	0.3742 ± 0.0519
ILF_R	0.7006 ± 0.2435	0.4799 ± 0.0684	0.5771 ± 0.0882	0.8951 ± 0.5654	0.7285 ± 0.2502	0.2715 ± 0.2502	0.3748 ± 0.0689
MCP	0.6850 ± 0.1771	0.4674 ± 0.0662	0.6025 ± 0.1354	0.9755 ± 0.3588	0.7861 ± 0.1311	0.2139 ± 0.1311	0.4316 ± 0.1100
MdLF_L	0.8025 ± 0.1923	0.3904 ± 0.1373	0.6433 ± 0.1247	2.7284 ± 4.9993	0.8178 ± 0.2170	0.1822 ± 0.2170	0.4371 ± 0.0945
MdLF_R	0.7530 ± 0.2253	0.4104 ± 0.1407	0.6292 ± 0.1647	1.7125 ± 1.1728	0.7355 ± 0.2440	0.2645 ± 0.2440	0.4381 ± 0.1223
OR_ML_L	0.9234 ± 0.0521	0.4670 ± 0.0689	0.6785 ± 0.0812	1.3203 ± 0.6718	0.9218 ± 0.0610	0.0782 ± 0.0610	0.4705 ± 0.0671
OR_ML_R	0.8520 ± 0.1234	0.3730 ± 0.1016	0.6928 ± 0.1032	2.2754 ± 1.1133	0.8479 ± 0.1626	0.1521 ± 0.1626	0.4754 ± 0.0725
POPT_L	0.8267 ± 0.0906	0.4973 ± 0.0628	0.5550 ± 0.0760	0.6965 ± 0.2918	0.8161 ± 0.0938	0.1839 ± 0.0938	0.4256 ± 0.0652
POPT_R	0.7955 ± 0.0784	0.4955 ± 0.0762	0.5842 ± 0.0783	0.8341 ± 0.5008	0.7581 ± 0.1239	0.2419 ± 0.1239	0.4496 ± 0.0664
PYT_L	0.8664 ± 0.0944	0.5673 ± 0.0345	0.5306 ± 0.0577	0.3379 ± 0.1038	0.8817 ± 0.0573	0.1183 ± 0.0573	0.4097 ± 0.0488
PYT_R	0.8661 ± 0.0857	0.5698 ± 0.0342	0.5360 ± 0.0446	0.3459 ± 0.0931	0.8913 ± 0.0514	0.1087 ± 0.0514	0.4169 ± 0.0405
SLF_L	0.5883 ± 0.1786	0.3268 ± 0.1011	0.5707 ± 0.1311	2.1464 ± 1.0886	0.5322 ± 0.1935	0.4678 ± 0.1935	0.3934 ± 0.0924
SLF_R	0.6989 ± 0.1487	0.4024 ± 0.1100	0.6033 ± 0.0863	1.6959 ± 1.1546	0.6568 ± 0.2123	0.3432 ± 0.2123	0.4090 ± 0.0634
UF_L	0.6763 ± 0.2320	0.3121 ± 0.1300	0.5507 ± 0.1630	2.2471 ± 1.1113	0.6790 ± 0.2396	0.3210 ± 0.2396	0.4644 ± 0.0975
UF_R	0.7524 ± 0.1792	0.3761 ± 0.1096	0.5588 ± 0.1258	1.5944 ± 0.9047	0.7814 ± 0.1933	0.2186 ± 0.1933	0.3827 ± 0.1084

Tab. S3 | Similarity, coverage, and validity of population bundles generated with ATM. Values are presented as mean ± standard deviation. For each metric, the arrow indicates preference: ↑ higher is better, ↓ lower is better.

Bundles	BA (↑)	Coverage			Validity		ACC (↑)
		Dice (↑)	Overlap (↑)	Overreach (↓)	VS (↑)	IS (↓)	
AF_L	0.5896 ± 0.1968	0.3597 ± 0.0730	0.5106 ± 0.0801	1.4501 ± 0.7261	0.6660 ± 0.2096	0.3340 ± 0.2096	0.2857 ± 0.0564
AF_R	0.5975 ± 0.1907	0.3038 ± 0.0909	0.5949 ± 0.1069	2.8967 ± 2.0491	0.6267 ± 0.2511	0.3733 ± 0.2511	0.3356 ± 0.0710
CC_Fr_1	0.8301 ± 0.2294	0.4825 ± 0.0743	0.5008 ± 0.1101	0.5910 ± 0.3828	0.8806 ± 0.2203	0.1194 ± 0.2203	0.3046 ± 0.0760
CC_Fr_2	0.8686 ± 0.1476	0.4584 ± 0.0672	0.4222 ± 0.1053	0.4092 ± 0.2475	0.9243 ± 0.1233	0.0757 ± 0.1233	0.2681 ± 0.0702
CC_Oc	0.6481 ± 0.2763	0.4421 ± 0.0730	0.4643 ± 0.0932	0.6429 ± 0.2858	0.7993 ± 0.2014	0.2007 ± 0.2014	0.2446 ± 0.0582
CC_Pa	0.8864 ± 0.0695	0.5000 ± 0.0498	0.4662 ± 0.0782	0.3944 ± 0.1725	0.9677 ± 0.0539	0.0323 ± 0.0539	0.2897 ± 0.0567
CC_Pr_Po	0.8833 ± 0.0926	0.4882 ± 0.0560	0.4277 ± 0.0651	0.3236 ± 0.1348	0.9523 ± 0.0874	0.0477 ± 0.0874	0.2690 ± 0.0426
CG_L	0.7990 ± 0.2109	0.3744 ± 0.0752	0.5560 ± 0.0808	1.5826 ± 0.9405	0.8330 ± 0.2343	0.1670 ± 0.2343	0.3307 ± 0.0609
CG_R	0.8279 ± 0.1766	0.3811 ± 0.0768	0.6124 ± 0.0983	1.7782 ± 1.1112	0.8668 ± 0.1639	0.1332 ± 0.1639	0.3675 ± 0.0941
FAT_L	0.8750 ± 0.0986	0.5141 ± 0.0579	0.5641 ± 0.0806	0.6427 ± 0.2650	0.9392 ± 0.0550	0.0608 ± 0.0550	0.3361 ± 0.0607
FAT_R	0.8730 ± 0.1084	0.4923 ± 0.0560	0.5678 ± 0.0813	0.7534 ± 0.2757	0.9327 ± 0.0524	0.0673 ± 0.0524	0.3429 ± 0.0704
FPT_L	0.6493 ± 0.1950	0.4229 ± 0.0687	0.5014 ± 0.0830	0.9276 ± 0.5102	0.7647 ± 0.1758	0.2353 ± 0.1758	0.3200 ± 0.0643
FPT_R	0.6438 ± 0.1783	0.4263 ± 0.0754	0.5108 ± 0.0599	0.9945 ± 0.6819	0.7557 ± 0.1566	0.2443 ± 0.1566	0.3275 ± 0.0514
IFOF_L	0.5622 ± 0.2387	0.3981 ± 0.0446	0.4345 ± 0.0588	0.7589 ± 0.2397	0.7816 ± 0.1733	0.2184 ± 0.1733	0.2475 ± 0.0474
IFOF_R	0.6791 ± 0.1807	0.4254 ± 0.0242	0.4772 ± 0.0437	0.7708 ± 0.1830	0.8228 ± 0.1277	0.1772 ± 0.1277	0.2743 ± 0.0268
ILF_L	0.5218 ± 0.2511	0.3987 ± 0.0739	0.4533 ± 0.0661	0.9044 ± 0.5662	0.7024 ± 0.2466	0.2976 ± 0.2466	0.2352 ± 0.0502
ILF_R	0.5636 ± 0.1846	0.4001 ± 0.0533	0.4610 ± 0.0590	0.8820 ± 0.4082	0.7354 ± 0.1965	0.2646 ± 0.1965	0.2439 ± 0.0371
MCP	0.2679 ± 0.2247	0.2730 ± 0.0919	0.3895 ± 0.1286	1.5204 ± 0.6076	0.3534 ± 0.2273	0.6466 ± 0.2273	0.2125 ± 0.0945
MdLF_L	0.6222 ± 0.2553	0.3324 ± 0.1275	0.5314 ± 0.0899	2.7915 ± 3.7243	0.7076 ± 0.2957	0.2924 ± 0.2957	0.2936 ± 0.0658
MdLF_R	0.6922 ± 0.2331	0.3437 ± 0.1372	0.5959 ± 0.1059	3.7042 ± 5.6339	0.7362 ± 0.2850	0.2638 ± 0.2850	0.3395 ± 0.0729
OR_ML_L	0.7308 ± 0.1932	0.3737 ± 0.0838	0.5202 ± 0.1143	1.3397 ± 0.6173	0.8252 ± 0.1911	0.1748 ± 0.1911	0.2868 ± 0.0763
OR_ML_R	0.6258 ± 0.2260	0.2692 ± 0.0876	0.5034 ± 0.1184	2.9722 ± 2.9050	0.6710 ± 0.2433	0.3290 ± 0.2433	0.2853 ± 0.0860
POPT_L	0.6901 ± 0.2109	0.4031 ± 0.1121	0.4618 ± 0.1268	0.8140 ± 0.4214	0.8272 ± 0.1876	0.1728 ± 0.1876	0.2956 ± 0.0890
POPT_R	0.6309 ± 0.2113	0.3951 ± 0.1170	0.4713 ± 0.1304	0.9424 ± 0.6161	0.7784 ± 0.1964	0.2216 ± 0.1964	0.3013 ± 0.0886
PYT_L	0.7064 ± 0.1879	0.4709 ± 0.1044	0.4503 ± 0.1099	0.4261 ± 0.1554	0.8831 ± 0.1152	0.1169 ± 0.1152	0.2895 ± 0.0748
PYT_R	0.7117 ± 0.2190	0.4863 ± 0.1078	0.4788 ± 0.1075	0.4548 ± 0.1500	0.8657 ± 0.1888	0.1343 ± 0.1888	0.3142 ± 0.0740
SLF_L	0.5743 ± 0.1829	0.2829 ± 0.1013	0.5723 ± 0.0913	3.4273 ± 3.3740	0.5893 ± 0.2436	0.4107 ± 0.2436	0.3196 ± 0.0581
SLF_R	0.6784 ± 0.1883	0.3542 ± 0.1168	0.5664 ± 0.0816	2.2660 ± 2.0499	0.6990 ± 0.2626	0.3010 ± 0.2626	0.3162 ± 0.0487
UF_L	0.4234 ± 0.2208	0.2367 ± 0.1100	0.4738 ± 0.1225	3.3522 ± 2.7458	0.5161 ± 0.2658	0.4839 ± 0.2658	0.2469 ± 0.0706
UF_R	0.5414 ± 0.2132	0.2866 ± 0.1050	0.5283 ± 0.0836	3.2576 ± 3.7658	0.6440 ± 0.2089	0.3560 ± 0.2089	0.2888 ± 0.0576

Tab. S4 | Similarity, coverage, and validity of bundles generated with MRtrix. Values are presented as mean ± standard deviation. For each metric, the arrow indicates preference: ↑ higher is better, ↓ lower is better.

Bundles	BA (↑)	Coverage			Validity		ACC (↑)
		Dice (↑)	Overlap (↑)	Overreach (↓)	VS (↑)	IS (↓)	
AF_L	0.4549 ± 0.3371	0.2663 ± 0.2359	0.3421 ± 0.3366	0.5477 ± 0.7266	0.7137 ± 0.3372	0.2863 ± 0.3372	0.2809 ± 0.2761
AF_R	0.2737 ± 0.2896	0.1879 ± 0.1989	0.2953 ± 0.3216	0.8315 ± 1.1268	0.4125 ± 0.3904	0.5875 ± 0.3904	0.2459 ± 0.2663
CC_Fr_1	0.3922 ± 0.3396	0.2511 ± 0.2337	0.2459 ± 0.2755	0.2418 ± 0.3791	0.6648 ± 0.3464	0.3352 ± 0.3464	0.2019 ± 0.2335
CC_Fr_2	0.3598 ± 0.2485	0.2236 ± 0.2097	0.2360 ± 0.2404	0.3767 ± 0.4791	0.6894 ± 0.3198	0.3106 ± 0.3198	0.1993 ± 0.2089
CC_Oc	0.4421 ± 0.4148	0.2490 ± 0.2509	0.1971 ± 0.2134	0.0818 ± 0.1049	0.5333 ± 0.4942	0.4667 ± 0.4942	0.1689 ± 0.1798
CC_Pa	0.4193 ± 0.2575	0.2483 ± 0.2557	0.2773 ± 0.2909	0.3523 ± 0.4663	0.7073 ± 0.3504	0.2927 ± 0.3504	0.2377 ± 0.2472
CC_Pr_Po	0.4346 ± 0.3115	0.2592 ± 0.2525	0.2893 ± 0.2939	0.3488 ± 0.4163	0.7432 ± 0.3368	0.2568 ± 0.3368	0.2506 ± 0.2536
CG_L	0.2420 ± 0.1963	0.1540 ± 0.1228	0.1029 ± 0.0904	0.1099 ± 0.1007	0.4874 ± 0.3260	0.5126 ± 0.3260	0.0863 ± 0.0741
CG_R	0.2915 ± 0.1757	0.1575 ± 0.1106	0.1035 ± 0.0837	0.1245 ± 0.1404	0.6501 ± 0.3078	0.3499 ± 0.3078	0.0863 ± 0.0686
FAT_L	0.3268 ± 0.2273	0.2446 ± 0.1324	0.1992 ± 0.1268	0.3268 ± 0.1688	0.5870 ± 0.2809	0.4130 ± 0.2809	0.1387 ± 0.1199
FAT_R	0.3842 ± 0.2323	0.2438 ± 0.1558	0.2069 ± 0.1742	0.3102 ± 0.1838	0.6175 ± 0.2739	0.3825 ± 0.2739	0.1489 ± 0.1563
FPT_L	0.3752 ± 0.3919	0.2449 ± 0.2731	0.2401 ± 0.2703	0.2041 ± 0.2738	0.4817 ± 0.4829	0.5183 ± 0.4829	0.2062 ± 0.2318
FPT_R	0.3774 ± 0.3828	0.2455 ± 0.2643	0.2734 ± 0.3093	0.3078 ± 0.4459	0.5300 ± 0.4605	0.4700 ± 0.4605	0.2342 ± 0.2651
IFOF_L	0.3330 ± 0.3610	0.1813 ± 0.2534	0.2147 ± 0.3251	0.2455 ± 0.4379	0.6758 ± 0.3263	0.3242 ± 0.3263	0.1753 ± 0.2629
IFOF_R	0.3481 ± 0.3431	0.1939 ± 0.2426	0.2358 ± 0.3385	0.2813 ± 0.4158	0.7088 ± 0.3170	0.2912 ± 0.3170	0.1886 ± 0.2713
ILF_L	0.5659 ± 0.2647	0.3942 ± 0.1948	0.3947 ± 0.2508	0.4124 ± 0.3797	0.8032 ± 0.2316	0.1968 ± 0.2316	0.3002 ± 0.2032
ILF_R	0.5628 ± 0.2940	0.4024 ± 0.2029	0.4139 ± 0.2626	0.4260 ± 0.3289	0.8132 ± 0.2615	0.1868 ± 0.2615	0.3144 ± 0.2158
MCP	0.3373 ± 0.3643	0.2495 ± 0.2673	0.2823 ± 0.3177	0.3248 ± 0.3911	0.4891 ± 0.4728	0.5109 ± 0.4728	0.2269 ± 0.2516
MdLF_L	0.3488 ± 0.2931	0.2168 ± 0.1948	0.2150 ± 0.2321	0.4616 ± 0.7588	0.6198 ± 0.3705	0.3802 ± 0.3705	0.1702 ± 0.1916
MdLF_R	0.4589 ± 0.2826	0.2369 ± 0.2252	0.2779 ± 0.2721	0.7979 ± 1.4877	0.7180 ± 0.3843	0.2820 ± 0.3843	0.2211 ± 0.2249
OR_ML_L	0.2795 ± 0.3064	0.0681 ± 0.0989	0.0405 ± 0.0624	0.0197 ± 0.0315	0.4650 ± 0.4643	0.5350 ± 0.4643	0.0357 ± 0.0526
OR_ML_R	0.2628 ± 0.2712	0.0676 ± 0.0909	0.0399 ± 0.0549	0.0303 ± 0.0661	0.4434 ± 0.4451	0.5566 ± 0.4451	0.0352 ± 0.0473
POPT_L	0.3535 ± 0.3461	0.2329 ± 0.2307	0.2057 ± 0.2071	0.1951 ± 0.2571	0.5008 ± 0.4808	0.4992 ± 0.4808	0.1774 ± 0.1793
POPT_R	0.3828 ± 0.3772	0.2556 ± 0.2490	0.2474 ± 0.2486	0.2426 ± 0.3085	0.4842 ± 0.4678	0.5158 ± 0.4678	0.2124 ± 0.2134
PYT_L	0.4668 ± 0.4498	0.3359 ± 0.3240	0.3242 ± 0.3169	0.1559 ± 0.1649	0.5191 ± 0.4988	0.4809 ± 0.4988	0.2794 ± 0.2738
PYT_R	0.5850 ± 0.3974	0.3490 ± 0.3331	0.3464 ± 0.3357	0.1662 ± 0.1688	0.7184 ± 0.4481	0.2816 ± 0.4481	0.2994 ± 0.2893
SLF_L	0.4033 ± 0.2520	0.2403 ± 0.1752	0.3442 ± 0.3181	1.0933 ± 1.8578	0.6725 ± 0.3218	0.3275 ± 0.3218	0.2776 ± 0.2604
SLF_R	0.3886 ± 0.2646	0.2575 ± 0.2063	0.3155 ± 0.2964	0.6777 ± 1.0080	0.6862 ± 0.3593	0.3138 ± 0.3593	0.2520 ± 0.2415
UF_L	0.3474 ± 0.2888	0.2530 ± 0.1772	0.3435 ± 0.2573	1.2927 ± 1.5054	0.5910 ± 0.3180	0.4090 ± 0.3180	0.2622 ± 0.2113
UF_R	0.4388 ± 0.2874	0.2897 ± 0.1755	0.3755 ± 0.2702	1.1238 ± 1.7107	0.6868 ± 0.2841	0.3132 ± 0.2841	0.2799 ± 0.2206

Tab. S5 | Similarity, coverage, and validity of population bundles generated with RecobundlesX. Values are presented as mean ± standard deviation. For each metric, the arrow indicates preference: ↑ higher is better, ↓ lower is better.

Bundles	BA (↑)	Coverage			Validity		ACC (↑)
		Dice (↑)	Overlap (↑)	Overreach (↓)	VS (↑)	IS (↓)	
AF_L	0.6292 ± 0.1495	0.3523 ± 0.0667	0.5329 ± 0.0711	1.6392 ± 0.8364	0.5954 ± 0.2042	0.4046 ± 0.2042	0.3077 ± 0.0644
AF_R	0.5541 ± 0.1369	0.2776 ± 0.0924	0.5788 ± 0.0928	3.2946 ± 2.3145	0.4686 ± 0.1955	0.5314 ± 0.1955	0.3224 ± 0.0892
CC_Fr_1	0.2417 ± 0.2213	0.2709 ± 0.0329	0.2052 ± 0.0450	0.2987 ± 0.1531	0.4619 ± 0.2753	0.5381 ± 0.2753	0.1032 ± 0.0406
CC_Fr_2	0.5255 ± 0.1936	0.3143 ± 0.0540	0.2615 ± 0.0509	0.4174 ± 0.2474	0.7674 ± 0.1836	0.2326 ± 0.1836	0.1560 ± 0.0423
CC_Oc	0.5984 ± 0.2261	0.3213 ± 0.0502	0.2582 ± 0.0470	0.3502 ± 0.1354	0.7690 ± 0.1932	0.2310 ± 0.1932	0.1587 ± 0.0354
CC_Pa	0.7669 ± 0.1166	0.4004 ± 0.0415	0.3813 ± 0.0481	0.5327 ± 0.2108	0.8209 ± 0.0726	0.1791 ± 0.0726	0.2516 ± 0.0398
CC_Pr_Po	0.7306 ± 0.1673	0.3582 ± 0.0624	0.3062 ± 0.0510	0.4139 ± 0.1517	0.7544 ± 0.1748	0.2456 ± 0.1748	0.2127 ± 0.0451
CG_L	0.4952 ± 0.2307	0.2987 ± 0.0799	0.3872 ± 0.0640	1.3788 ± 0.7905	0.5585 ± 0.2572	0.4415 ± 0.2572	0.2324 ± 0.0596
CG_R	0.5821 ± 0.1856	0.2992 ± 0.0716	0.4061 ± 0.0663	1.4719 ± 0.9060	0.5927 ± 0.2088	0.4073 ± 0.2088	0.2392 ± 0.0548
FAT_L	0.4470 ± 0.1651	0.1911 ± 0.0550	0.1397 ± 0.0367	0.3451 ± 0.1503	0.7006 ± 0.2469	0.2994 ± 0.2469	0.0801 ± 0.0295
FAT_R	0.5194 ± 0.2347	0.2456 ± 0.0666	0.1896 ± 0.0483	0.3685 ± 0.1486	0.7825 ± 0.2316	0.2175 ± 0.2316	0.1118 ± 0.0328
FPT_L	0.6110 ± 0.1684	0.3147 ± 0.0713	0.2647 ± 0.0659	0.4382 ± 0.2653	0.8380 ± 0.1700	0.1620 ± 0.1700	0.1766 ± 0.0473
FPT_R	0.6065 ± 0.2053	0.3246 ± 0.0761	0.2717 ± 0.0568	0.4484 ± 0.3410	0.8121 ± 0.2032	0.1879 ± 0.2032	0.1808 ± 0.0403
IFOF_L	0.6790 ± 0.1889	0.4000 ± 0.0352	0.4085 ± 0.0548	0.6391 ± 0.2119	0.8578 ± 0.0951	0.1422 ± 0.0951	0.2428 ± 0.0470
IFOF_R	0.7177 ± 0.1820	0.4146 ± 0.0332	0.4208 ± 0.0465	0.6104 ± 0.1384	0.8627 ± 0.1090	0.1373 ± 0.1090	0.2535 ± 0.0404
ILF_L	0.7083 ± 0.1922	0.4029 ± 0.0689	0.3942 ± 0.0632	0.6187 ± 0.4298	0.8509 ± 0.1859	0.1491 ± 0.1859	0.2217 ± 0.0472
ILF_R	0.6627 ± 0.1771	0.4006 ± 0.0592	0.3969 ± 0.0474	0.6194 ± 0.3238	0.7953 ± 0.1801	0.2047 ± 0.1801	0.2203 ± 0.0332
MCP	0.6261 ± 0.2238	0.4197 ± 0.0866	0.5127 ± 0.1259	0.9374 ± 0.4264	0.8406 ± 0.1717	0.1594 ± 0.1717	0.3127 ± 0.0838
MdLF_L	0.6514 ± 0.2497	0.2961 ± 0.1042	0.3268 ± 0.0808	1.4348 ± 2.0163	0.7699 ± 0.2828	0.2301 ± 0.2828	0.1952 ± 0.0583
MdLF_R	0.7184 ± 0.2584	0.3245 ± 0.1130	0.3534 ± 0.0771	1.5316 ± 2.4494	0.8059 ± 0.2798	0.1941 ± 0.2798	0.2218 ± 0.0528
OR_ML_L	0.5774 ± 0.1592	0.3178 ± 0.0565	0.3937 ± 0.0613	1.1509 ± 0.5152	0.6314 ± 0.1724	0.3686 ± 0.1724	0.2297 ± 0.0472
OR_ML_R	0.4285 ± 0.1937	0.2546 ± 0.0889	0.4000 ± 0.1145	2.2460 ± 2.1154	0.4630 ± 0.1867	0.5370 ± 0.1867	0.2262 ± 0.0831
POPT_L	0.5974 ± 0.2028	0.2622 ± 0.0529	0.2208 ± 0.0473	0.4660 ± 0.1570	0.8217 ± 0.1960	0.1783 ± 0.1960	0.1561 ± 0.0376
POPT_R	0.6190 ± 0.1672	0.2785 ± 0.0393	0.2513 ± 0.0565	0.5452 ± 0.2428	0.7678 ± 0.1786	0.2322 ± 0.1786	0.1777 ± 0.0391
PYT_L	0.8087 ± 0.1652	0.3321 ± 0.0650	0.2333 ± 0.0573	0.1589 ± 0.0673	0.9555 ± 0.0836	0.0445 ± 0.0836	0.1694 ± 0.0462
PYT_R	0.8449 ± 0.1374	0.3365 ± 0.0643	0.2359 ± 0.0557	0.1547 ± 0.0550	0.9738 ± 0.0504	0.0262 ± 0.0504	0.1689 ± 0.0427
SLF_L	0.5144 ± 0.1938	0.2685 ± 0.0975	0.4601 ± 0.0873	2.6943 ± 2.5927	0.5086 ± 0.2261	0.4914 ± 0.2261	0.2471 ± 0.0642
SLF_R	0.4511 ± 0.2167	0.2915 ± 0.0947	0.3881 ± 0.0661	1.7064 ± 1.4795	0.5328 ± 0.2714	0.4672 ± 0.2714	0.1991 ± 0.0526
UF_L	0.4147 ± 0.2847	0.2466 ± 0.1029	0.3379 ± 0.0897	2.0120 ± 1.7374	0.4868 ± 0.3153	0.5132 ± 0.3153	0.1992 ± 0.0563
UF_R	0.5148 ± 0.2697	0.2806 ± 0.0986	0.3496 ± 0.0690	1.6912 ± 2.0572	0.5681 ± 0.3401	0.4319 ± 0.3401	0.2092 ± 0.0535

Tab. S6 | Similarity, coverage, and validity of bundles generated with TractSeg. Values are presented as mean ± standard deviation. For each metric, the arrow indicates preference: ↑ higher is better, ↓ lower is better.

Bundles	BA (↑)		Coverage		Validity		ACC (↑)
		Dice (↑)	Overlap (↑)	Overreach (↓)	VS (↑)	IS (↓)	
AF_L	0.3536 ± 0.3486	0.2439 ± 0.2362	0.2555 ± 0.2494	0.3186 ± 0.3487	0.3350 ± 0.3441	0.6650 ± 0.3441	0.2065 ± 0.2017
AF_R	0.3712 ± 0.3652	0.1944 ± 0.1966	0.2676 ± 0.2733	0.6834 ± 0.8672	0.3642 ± 0.3805	0.6358 ± 0.3805	0.2158 ± 0.2199
CG_L	0.3466 ± 0.3655	0.2263 ± 0.2341	0.3033 ± 0.3100	0.5803 ± 0.7515	0.2796 ± 0.3156	0.7204 ± 0.3156	0.2393 ± 0.2458
CG_R	0.3654 ± 0.3709	0.2358 ± 0.2445	0.3112 ± 0.3160	0.5664 ± 0.7405	0.2886 ± 0.3105	0.7114 ± 0.3105	0.2438 ± 0.2471
FPT_L	0.2819 ± 0.3316	0.1971 ± 0.2260	0.1584 ± 0.1857	0.1206 ± 0.1926	0.3333 ± 0.3822	0.6667 ± 0.3822	0.1370 ± 0.1604
FPT_R	0.2753 ± 0.3345	0.2018 ± 0.2307	0.1727 ± 0.2042	0.1483 ± 0.2281	0.3417 ± 0.3977	0.6583 ± 0.3977	0.1490 ± 0.1761
IFOF_L	0.1281 ± 0.2306	0.1131 ± 0.1791	0.0825 ± 0.1316	0.0480 ± 0.0896	0.1983 ± 0.3388	0.8017 ± 0.3388	0.0703 ± 0.1124
IFOF_R	0.1491 ± 0.2568	0.1209 ± 0.1930	0.0921 ± 0.1474	0.0572 ± 0.0941	0.2312 ± 0.3700	0.7688 ± 0.3700	0.0774 ± 0.1237
ILF_L	0.3871 ± 0.3579	0.1831 ± 0.1730	0.1317 ± 0.1353	0.0945 ± 0.1324	0.4890 ± 0.4432	0.5110 ± 0.4432	0.1109 ± 0.1131
ILF_R	0.3672 ± 0.3364	0.1844 ± 0.1701	0.1255 ± 0.1194	0.0701 ± 0.0839	0.4670 ± 0.4289	0.5330 ± 0.4289	0.1034 ± 0.0975
MCP	0.4208 ± 0.4082	0.3439 ± 0.2269	0.3299 ± 0.2612	0.2996 ± 0.3271	0.4227 ± 0.4099	0.5773 ± 0.4099	0.2578 ± 0.2254
MdLF_L	0.2658 ± 0.2915	0.1677 ± 0.1880	0.2656 ± 0.2725	1.5319 ± 3.3567	0.1494 ± 0.2144	0.8506 ± 0.2144	0.2071 ± 0.2098
MdLF_R	0.2855 ± 0.3033	0.1549 ± 0.1817	0.2426 ± 0.2494	1.6453 ± 3.5937	0.1606 ± 0.1908	0.8394 ± 0.1908	0.1898 ± 0.1946
POPT_L	0.3718 ± 0.3617	0.2355 ± 0.2287	0.1964 ± 0.1980	0.1455 ± 0.1979	0.3144 ± 0.3160	0.6856 ± 0.3160	0.1706 ± 0.1716
POPT_R	0.3557 ± 0.3421	0.2375 ± 0.2292	0.2012 ± 0.2011	0.1558 ± 0.2052	0.3011 ± 0.2994	0.6989 ± 0.2994	0.1762 ± 0.1757
UF_L	0.1692 ± 0.2455	0.1617 ± 0.1613	0.1450 ± 0.1481	0.3237 ± 0.4695	0.1426 ± 0.2190	0.8574 ± 0.2190	0.1173 ± 0.1201
UF_R	0.2145 ± 0.2932	0.1763 ± 0.1786	0.1716 ± 0.1875	0.4238 ± 0.8262	0.1419 ± 0.2387	0.8581 ± 0.2387	0.1324 ± 0.1455

Tab. S7 | Geometry and connectivity of bundles generated with ATM. Absolute percent differences are presented as mean ± standard deviation. A dash ′′−′ indicates an absent bundle.

Bundles	Geometry APD (↓)				Pearson (↑)	Connectivity APD (↓)			
	Mean Length	Span	Volume	Surface Area		Density	CPL	Efficiency	Modularity
AF_L	16.3782 ± 6.1705	11.9295 ± 9.4784	31.7351 ± 21.8256	48.1792 ± 28.5575	0.4791 ± 0.1895	71.5249 ± 43.9653	113.4541 ± 50.0194	147.1505 ± 30.8938	97.5305 ± 54.6368
AF_R	17.1918 ± 6.3730	10.4354 ± 6.7693	53.6095 ± 34.0503	64.8241 ± 34.2957	0.4818 ± 0.1450	84.7245 ± 39.3843	103.6713 ± 49.5844	162.7604 ± 19.4216	104.1599 ± 57.3431
CC_Fr_1	13.0568 ± 4.7349	12.0143 ± 9.6003	43.5380 ± 27.7355	30.6197 ± 18.7808	0.5115 ± 0.2119	77.4166 ± 75.8007	111.4594 ± 65.6328	116.9450 ± 63.9403	117.8747 ± 90.3340
CC_Fr_2	18.9026 ± 4.6200	23.4412 ± 9.4057	71.5429 ± 31.9900	33.3150 ± 19.6487	0.5941 ± 0.1848	53.6970 ± 44.9040	118.2380 ± 52.1721	120.4965 ± 46.8038	154.7775 ± 65.3555
CC_Oc	11.0030 ± 6.1956	10.9535 ± 7.9614	21.7280 ± 16.1675	29.7788 ± 16.9099	0.5395 ± 0.1194	26.4474 ± 18.3870	59.1651 ± 31.3135	119.3264 ± 24.0460	161.2685 ± 67.0146
CC_Pa	12.0806 ± 4.7389	17.5031 ± 10.8307	44.8484 ± 24.8365	23.9097 ± 18.3151	0.5634 ± 0.0817	22.6523 ± 13.1722	60.9603 ± 17.3834	91.3646 ± 23.7189	161.6402 ± 70.5580
CC_Pr_Po	14.5432 ± 6.2803	20.1826 ± 10.0380	62.9048 ± 18.3201	20.0317 ± 10.2214	0.5122 ± 0.1061	28.3413 ± 20.7427	68.2592 ± 32.2108	91.9094 ± 23.6135	179.1153 ± 45.5032
CG_L	11.7686 ± 8.1369	7.8983 ± 6.0824	43.0467 ± 31.4850	50.6015 ± 25.2450	0.5361 ± 0.1424	63.9521 ± 37.4996	80.8836 ± 57.6416	149.4564 ± 29.5670	105.5089 ± 84.1536
CG_R	14.4626 ± 5.5075	9.1331 ± 5.2334	42.4491 ± 31.5814	50.7069 ± 25.2823	0.4689 ± 0.1448	70.4776 ± 43.0928	91.8669 ± 52.1589	165.4556 ± 18.8717	119.0609 ± 76.6755
FAT_L	22.4657 ± 5.0508	16.0017 ± 5.7259	39.1771 ± 18.4583	33.0898 ± 21.5166	0.5763 ± 0.1146	36.7774 ± 45.0994	137.6900 ± 46.0749	90.8868 ± 38.1938	149.8390 ± 77.2425
FAT_R	24.5213 ± 5.0085	18.3513 ± 6.1703	33.3269 ± 21.8262	33.5119 ± 20.0700	0.6050 ± 0.1024	53.7432 ± 51.0949	135.9431 ± 51.0177	117.7373 ± 34.7561	138.4228 ± 71.0034
FPT_L	12.2648 ± 6.0247	8.7889 ± 6.0314	32.5808 ± 21.2695	29.1909 ± 25.6331	0.3836 ± 0.2600	96.7651 ± 51.7182	126.7067 ± 48.9923	162.8325 ± 34.8590	157.5421 ± 54.7153
FPT_R	12.6060 ± 6.6058	10.2867 ± 6.3087	36.9889 ± 19.8639	25.3915 ± 26.2654	0.3577 ± 0.2355	84.2431 ± 46.7789	138.3907 ± 51.2070	158.0912 ± 34.4026	108.2244 ± 66.8066
IFOF_L	8.1415 ± 5.1921	6.3258 ± 3.8312	22.8766 ± 14.6966	27.1602 ± 15.1998	0.5190 ± 0.1150	41.2266 ± 44.6142	90.4319 ± 47.4766	136.7237 ± 30.9487	99.6624 ± 59.8478
IFOF_R	6.4569 ± 3.9587	5.6194 ± 3.6281	19.9552 ± 11.0831	19.6940 ± 11.2226	0.5350 ± 0.1568	42.6251 ± 25.6825	76.4801 ± 52.7638	133.4644 ± 34.3376	119.1448 ± 53.8623
ILF_L	13.4611 ± 6.1576	8.9987 ± 5.0363	36.7725 ± 16.7823	36.6135 ± 28.1284	0.3397 ± 0.1190	49.4572 ± 44.9010	90.5238 ± 48.4326	130.3446 ± 36.1894	109.2040 ± 67.3970
ILF_R	15.7252 ± 10.8096	10.4027 ± 7.7099	34.3426 ± 19.5810	41.6718 ± 34.7760	0.3617 ± 0.1705	35.0685 ± 31.5976	75.5435 ± 33.9021	116.6163 ± 34.5262	122.6967 ± 75.3571
MCP	12.7934 ± 10.3452	18.9207 ± 15.4606	41.3428 ± 30.9230	58.8321 ± 36.0299	-	-	-	-	-
MdLF_L	9.5037 ± 8.1335	8.1337 ± 6.3170	41.3472 ± 38.9247	67.5858 ± 31.4190	0.4828 ± 0.1114	58.2989 ± 37.5180	74.9776 ± 34.2174	136.8213 ± 23.7961	96.1600 ± 67.7045
MdLF_R	13.5569 ± 6.2466	8.1571 ± 7.1910	34.5415 ± 31.7768	61.2891 ± 28.3313	0.4888 ± 0.1381	55.4073 ± 38.5082	73.2844 ± 24.6850	136.8928 ± 23.3823	112.0057 ± 72.1071
OR_ML_L	8.6944 ± 5.5935	8.0702 ± 5.2010	36.0904 ± 27.1998	53.1422 ± 24.7711	0.5025 ± 0.1239	43.8691 ± 36.3039	73.8708 ± 28.7495	130.0176 ± 27.2128	93.7145 ± 64.2978
OR_ML_R	6.5757 ± 5.3304	9.1249 ± 6.4066	59.6100 ± 30.9532	69.7776 ± 25.9030	0.3820 ± 0.1415	42.5585 ± 37.0258	72.2283 ± 30.7377	129.8328 ± 25.5756	62.2036 ± 59.0656
POPT_L	11.8635 ± 5.1048	7.9826 ± 4.4917	27.9131 ± 16.9057	24.9565 ± 15.8569	0.3151 ± 0.1891	56.1726 ± 42.1884	88.7277 ± 57.0255	127.6431 ± 47.9636	83.6436 ± 73.2118
POPT_R	12.8781 ± 4.3365	10.5900 ± 4.6060	30.6801 ± 19.0879	29.4745 ± 20.2749	0.2805 ± 0.1576	58.3800 ± 56.2500	115.2927 ± 47.8857	149.0068 ± 27.3337	87.8550 ± 57.3309
PYT_L	12.5660 ± 6.8230	10.3589 ± 6.6500	52.9165 ± 16.2892	11.0647 ± 9.7582	0.3818 ± 0.2085	59.2035 ± 51.5608	112.3338 ± 57.5605	118.9285 ± 47.3877	111.2434 ± 69.3786
PYT_R	10.5912 ± 6.6160	8.9615 ± 6.4546	51.4753 ± 13.1687	13.7194 ± 7.5828	0.3414 ± 0.2046	50.9705 ± 39.8734	120.1050 ± 45.0122	133.3500 ± 33.4591	83.5500 ± 63.0634
SLF_L	16.0281 ± 8.4210	10.6847 ± 8.2703	48.6050 ± 29.7418	69.8918 ± 27.8327	0.3965 ± 0.1766	113.5328 ± 53.9868	132.9065 ± 50.1194	175.9793 ± 16.6950	126.1223 ± 68.6660
SLF_R	14.1764 ± 7.4493	7.7023 ± 6.8997	43.9492 ± 29.5444	59.8345 ± 32.0203	0.4961 ± 0.1586	68.8449 ± 48.7939	102.3647 ± 44.0100	154.9991 ± 22.3582	108.8233 ± 66.3844
UF_L	9.5315 ± 7.0647	10.9449 ± 8.8163	48.5047 ± 25.9682	66.7306 ± 30.4244	0.5044 ± 0.1572	91.2972 ± 62.8138	68.1320 ± 59.6366	133.9668 ± 51.3670	106.1518 ± 83.9562
UF_R	12.1006 ± 6.5600	10.9832 ± 7.3216	30.0130 ± 26.1309	46.0246 ± 31.3479	0.4548 ± 0.1819	63.6916 ± 67.1990	70.4086 ± 44.0482	105.7981 ± 56.7679	132.1564 ± 74.7255

Tab. S8 | Geometry and connectivity of bundles generated with ATM Population. Absolute percent differences are presented as mean ± standard deviation. A dash ′′−′ indicates an absent bundle.

Bundles	Geometry APD (↓)				Pearson (↑)	Connectivity APD (↓)			
	Mean Length	Span	Volume	Surface Area		Density	CPL	Efficiency	Modularity
AF_L	8.2681 ± 5.5412	14.2664 ± 11.3771	36.5587 ± 27.8512	58.2076 ± 30.1191	0.6093 ± 0.2563	68.3826 ± 45.9000	65.8058 ± 53.2852	73.2295 ± 51.7421	100.3317 ± 61.3653
AF_R	8.7775 ± 6.7585	11.2794 ± 6.2682	72.4627 ± 42.1247	80.6858 ± 38.2663	0.6770 ± 0.2179	91.6035 ± 41.0358	60.8566 ± 50.2756	105.4585 ± 44.3858	115.3084 ± 51.3482
CC_Fr_1	9.6153 ± 4.6670	17.6100 ± 13.7836	43.5457 ± 27.9191	39.0434 ± 23.6111	0.7110 ± 0.3005	57.2013 ± 63.9635	46.3378 ± 53.9471	55.7444 ± 64.1375	86.5410 ± 91.8326
CC_Fr_2	8.1296 ± 6.4321	20.5464 ± 21.6984	70.1547 ± 32.4500	36.3252 ± 20.2694	0.9455 ± 0.0687	62.5605 ± 57.5518	55.6939 ± 57.9776	69.7374 ± 63.4836	102.4558 ± 91.3157
CC_Oc	6.4214 ± 5.1846	14.8794 ± 9.3793	29.8781 ± 19.1207	23.4589 ± 20.0809	0.7096 ± 0.1600	27.8331 ± 17.9019	21.0375 ± 35.0448	27.7586 ± 21.5447	28.3922 ± 57.5328
CC_Pa	7.5740 ± 5.1128	17.4426 ± 16.0180	55.1048 ± 23.9001	23.7437 ± 17.2954	0.7600 ± 0.1261	24.8944 ± 19.3461	16.2870 ± 18.9218	29.8510 ± 21.5127	70.0749 ± 92.7922
CC_Pr_Po	8.7810 ± 5.8416	19.3382 ± 19.3976	68.0227 ± 20.2694	21.8471 ± 11.5451	0.7070 ± 0.1230	31.4879 ± 22.4558	30.6227 ± 33.3437	31.4333 ± 21.6987	75.5637 ± 94.9329
CG_L	8.2210 ± 7.3928	7.8397 ± 6.0608	43.6571 ± 31.0916	51.1256 ± 28.7002	0.7725 ± 0.1736	58.4004 ± 43.9012	62.9614 ± 55.5418	61.4638 ± 46.0425	121.7211 ± 72.6422
CG_R	7.4037 ± 5.2793	6.2408 ± 5.7435	49.0586 ± 31.7803	60.1993 ± 25.5450	0.6313 ± 0.2466	79.4655 ± 40.7874	60.4525 ± 55.6768	92.5929 ± 39.5268	136.7875 ± 72.6959
FAT_L	6.0026 ± 3.5007	8.3885 ± 5.4510	30.6085 ± 18.6227	40.4849 ± 18.2571	0.8480 ± 0.1544	39.5668 ± 46.3216	69.6156 ± 56.6225	35.5501 ± 47.3795	46.5722 ± 82.5017
FAT_R	6.4289 ± 4.5804	8.8039 ± 5.4292	25.9203 ± 16.0835	44.6690 ± 18.5711	0.8441 ± 0.1775	60.0954 ± 50.4447	66.2114 ± 56.8914	61.4544 ± 47.6911	97.7640 ± 95.0988
FPT_L	9.1178 ± 5.7209	10.3170 ± 6.0989	28.0925 ± 22.2866	35.7028 ± 25.5470	0.5185 ± 0.3586	78.1981 ± 56.8633	88.2401 ± 51.3557	83.3178 ± 60.9929	143.3341 ± 71.4695
FPT_R	7.9173 ± 5.4711	10.9063 ± 5.8980	33.3736 ± 20.2079	34.3479 ± 25.4089	0.5590 ± 0.3237	88.2265 ± 49.0428	100.9288 ± 51.3037	90.5799 ± 50.5607	112.0276 ± 63.3164
IFOF_L	7.1495 ± 3.1609	6.4071 ± 3.9407	24.6019 ± 18.6905	24.9001 ± 13.2942	0.7570 ± 0.1331	47.3148 ± 46.4744	53.2249 ± 55.7457	50.2776 ± 44.7868	102.7448 ± 69.2564
IFOF_R	7.4404 ± 5.4089	8.4976 ± 5.7082	17.3303 ± 13.6081	28.5224 ± 12.7466	0.7350 ± 0.2185	50.2533 ± 34.8227	73.4665 ± 46.2785	54.2212 ± 35.0819	64.4367 ± 57.9695
ILF_L	6.5726 ± 4.3102	5.4704 ± 3.6997	38.5914 ± 16.3675	34.0845 ± 25.7503	0.4554 ± 0.1498	46.5387 ± 45.8471	63.3220 ± 49.5501	49.8823 ± 46.3211	94.0481 ± 70.6792
ILF_R	4.5974 ± 3.7230	3.6208 ± 3.4115	28.7556 ± 16.8537	33.4042 ± 21.2228	0.4992 ± 0.1836	25.9435 ± 26.2995	44.8811 ± 35.9200	29.2348 ± 28.6231	108.3219 ± 81.1134
MCP	11.0932 ± 8.8940	18.4401 ± 14.4899	37.3062 ± 23.8289	52.4690 ± 35.4656	-	-	-	-	-
MdLF_L	12.9152 ± 7.9837	12.4125 ± 7.4192	56.8275 ± 51.4548	55.9288 ± 36.3817	0.5880 ± 0.2023	63.3500 ± 40.0921	38.1713 ± 46.8170	70.8264 ± 40.8476	129.2501 ± 75.1958
MdLF_R	8.2383 ± 6.4699	9.8178 ± 6.2262	57.0397 ± 55.4893	69.8060 ± 39.2042	0.6955 ± 0.1969	65.2442 ± 45.0950	23.0411 ± 27.3208	73.2535 ± 49.0347	143.0954 ± 71.1348
OR_ML_L	6.0029 ± 4.2230	7.2800 ± 5.4194	35.8274 ± 26.3302	46.7624 ± 25.7352	0.5908 ± 0.2655	40.3678 ± 37.9401	27.4269 ± 31.6121	42.3437 ± 36.6582	96.1964 ± 68.5150
OR_ML_R	5.5023 ± 7.1583	9.0036 ± 6.7062	67.1728 ± 44.1575	69.8250 ± 35.0312	0.3853 ± 0.2079	45.0728 ± 46.8361	28.0068 ± 34.4028	51.3883 ± 52.2398	88.4322 ± 72.3478
POPT_L	9.7862 ± 5.0287	12.0157 ± 5.3240	35.1710 ± 36.5056	34.2114 ± 32.2673	0.5543 ± 0.2312	62.7392 ± 55.0573	68.5180 ± 44.1594	67.4197 ± 53.4306	114.4827 ± 76.1197
POPT_R	7.8847 ± 4.5397	13.6560 ± 6.4301	39.4922 ± 35.4703	34.3353 ± 31.6933	0.4496 ± 0.2703	72.9346 ± 59.1155	81.7816 ± 53.3687	75.4638 ± 59.9272	99.2904 ± 69.2433
PYT_L	9.4536 ± 6.8190	13.7114 ± 6.8253	53.2839 ± 33.1885	19.4918 ± 31.1406	0.5276 ± 0.2929	51.8281 ± 47.8417	87.9006 ± 49.4102	56.8794 ± 48.1711	115.6433 ± 75.0632
PYT_R	16.3759 ± 37.9825	20.1285 ± 37.3103	48.1364 ± 33.4872	24.0390 ± 37.3985	0.5335 ± 0.3247	51.4781 ± 54.6853	90.9379 ± 48.2084	53.2173 ± 55.1013	79.8352 ± 60.0055
SLF_L	8.3342 ± 6.5310	10.1870 ± 7.6870	70.3764 ± 45.8906	84.5230 ± 35.4746	0.5399 ± 0.2405	105.8984 ± 56.9638	65.8818 ± 58.1258	105.9773 ± 56.7648	94.9918 ± 75.9562
SLF_R	6.2091 ± 5.0796	7.2702 ± 6.3994	55.2501 ± 41.2581	67.1194 ± 37.4059	0.7372 ± 0.1544	58.0585 ± 50.4667	63.9556 ± 46.6864	58.9023 ± 51.2681	101.8658 ± 69.2364
UF_L	10.7290 ± 8.1664	12.6763 ± 8.1753	77.9371 ± 47.3195	74.2109 ± 34.4579	0.5088 ± 0.2538	100.1906 ± 68.6315	60.5244 ± 56.0696	95.3395 ± 67.1626	73.0715 ± 88.0472
UF_R	5.8827 ± 4.5118	12.6338 ± 8.9538	60.3274 ± 48.6066	66.0857 ± 35.3441	0.4984 ± 0.2579	73.6251 ± 67.7156	62.5297 ± 45.6729	78.1092 ± 65.3084	126.0346 ± 80.7302

Tab. S9 | Geometry and connectivity of bundles generated with MRtrix. Absolute percent differences are presented as mean ± standard deviation. A dash ′′−′ indicates an absent bundle.

Bundles	Geometry APD (↓)				Pearson (↑)	Connectivity APD (↓)			
	Mean Length	Span	Volume	Surface Area		Density	CPL	Efficiency	Modularity
AF_L	26.0359 ± 51.9210	33.1879 ± 50.9823	115.6806 ± 82.4487	111.5583 ± 71.4228	0.1288 ± 0.2031	155.4999 ± 56.9065	117.0224 ± 56.1666	140.2380 ± 70.3859	144.0560 ± 77.5087
AF_R	78.6305 ± 91.1641	78.9744 ± 91.0084	129.1711 ± 77.4854	128.3869 ± 73.6676	0.1117 ± 0.1550	155.7848 ± 59.6146	147.6481 ± 49.3685	142.1600 ± 71.7414	170.5758 ± 58.1518
CC_Fr_1	28.7244 ± 53.0094	48.7440 ± 50.4808	139.3548 ± 65.9910	106.2353 ± 71.6438	0.0000 ± 0.0000	130.2412 ± 95.1149	42.4475 ± 64.7078	130.0437 ± 94.9712	72.5120 ± 95.9289
CC_Fr_2	29.2831 ± 54.1055	73.4090 ± 52.2994	118.3693 ± 84.4848	112.6943 ± 67.5447	0.1505 ± 0.2532	168.3217 ± 49.1476	105.7133 ± 76.6728	144.8372 ± 75.3955	92.5256 ± 95.0340
CC_Oc	99.4880 ± 93.1062	96.5829 ± 95.9032	152.1405 ± 53.4519	123.5757 ± 76.9264	0.2104 ± 0.2296	145.6135 ± 59.5479	124.2123 ± 70.8672	121.4100 ± 80.1973	109.3582 ± 94.9602
CC_Pa	32.3902 ± 55.0602	79.3137 ± 50.1319	121.2504 ± 84.1238	125.1632 ± 72.4351	0.1781 ± 0.1405	133.3038 ± 67.0241	83.1278 ± 54.3227	120.0501 ± 70.4154	133.8488 ± 87.0529
CC_Pr_Po	35.8845 ± 65.4474	84.7579 ± 54.7444	114.3801 ± 88.0425	117.6074 ± 76.8859	0.1923 ± 0.1966	157.2062 ± 56.7611	133.5419 ± 60.1942	141.5786 ± 68.0989	84.8139 ± 96.3274
CG_L	32.9040 ± 57.5752	35.4116 ± 56.6073	170.8239 ± 28.2244	124.6225 ± 48.7377	0.1297 ± 0.1800	136.9602 ± 70.2673	116.9628 ± 61.4698	134.8038 ± 60.2484	139.8469 ± 83.9488
CG_R	13.1741 ± 11.7343	14.8671 ± 12.1946	169.8487 ± 25.4742	120.2559 ± 46.2748	0.0839 ± 0.1224	144.3006 ± 67.0640	124.5749 ± 62.8382	141.0171 ± 61.4804	167.4929 ± 70.6678
FAT_L	10.0875 ± 5.3252	11.1719 ± 7.9812	120.2347 ± 36.2406	44.6084 ± 43.0500	0.1111 ± 0.1945	163.5489 ± 52.8303	117.1019 ± 63.9119	142.0765 ± 72.4616	70.5682 ± 95.5498
FAT_R	10.2472 ± 9.5959	10.1540 ± 7.3847	125.3196 ± 45.9284	54.5024 ± 41.6871	0.1413 ± 0.2172	150.7412 ± 65.5731	122.7482 ± 58.0967	134.7877 ± 80.3492	98.7377 ± 97.0361
FPT_L	105.5503 ± 94.5004	106.4424 ± 93.6309	132.4727 ± 76.0569	117.7583 ± 89.3766	0.0046 ± 0.0221	189.7675 ± 40.3624	95.1517 ± 73.9796	188.5845 ± 41.3314	116.6060 ± 98.5502
FPT_R	90.8325 ± 92.3657	90.5563 ± 92.5631	128.6733 ± 78.4696	118.3725 ± 84.6276	−0.0000 ± 0.0001	177.5951 ± 59.7749	94.2654 ± 67.7910	175.5936 ± 63.6823	158.2940 ± 81.2032
IFOF_L	26.2345 ± 48.3497	27.6380 ± 48.0811	151.2080 ± 73.1127	141.9719 ± 69.5164	0.0977 ± 0.1954	183.2528 ± 41.8492	136.0062 ± 62.3505	180.8082 ± 32.1686	159.7859 ± 71.0127
IFOF_R	28.5204 ± 48.2261	31.3790 ± 47.2552	143.2197 ± 80.3972	136.9411 ± 61.2780	0.1710 ± 0.2284	167.5239 ± 42.9473	117.8082 ± 61.1774	145.9972 ± 61.6713	174.4042 ± 56.6196
ILF_L	8.2873 ± 4.8752	8.3210 ± 5.2733	93.5844 ± 62.7054	61.8256 ± 45.6450	0.1479 ± 0.1544	124.7652 ± 50.4937	55.7046 ± 50.5699	80.9878 ± 61.8907	133.4378 ± 71.8226
ILF_R	16.4147 ± 39.4264	16.6687 ± 39.3774	88.0284 ± 64.3455	60.3832 ± 49.7610	0.1827 ± 0.1490	102.0480 ± 54.3000	60.4502 ± 48.9603	68.1857 ± 48.9713	152.8993 ± 54.8246
MCP	100.8919 ± 95.0341	115.5187 ± 81.9396	123.6511 ± 80.9590	114.5660 ± 84.3858	-	-	-	-	-
MdLF_L	24.7102 ± 50.8673	24.1749 ± 51.1056	135.2740 ± 61.3215	99.2931 ± 66.7445	0.2446 ± 0.2150	132.3826 ± 58.5039	83.4365 ± 62.3007	112.9312 ± 62.6934	169.1429 ± 52.1627
MdLF_R	25.2688 ± 50.6420	23.8214 ± 51.1456	121.5002 ± 71.5644	99.3632 ± 73.7098	0.2883 ± 0.2093	117.8580 ± 56.0832	81.2787 ± 55.0951	91.4944 ± 61.1678	134.0926 ± 79.8035
OR_ML_L	99.5912 ± 96.5187	98.6680 ± 97.3852	191.6138 ± 13.5428	174.8292 ± 33.0054	0.0910 ± 0.1364	182.6335 ± 23.4281	144.3829 ± 57.6349	161.2216 ± 55.0029	141.6259 ± 80.3243
OR_ML_R	100.7051 ± 95.8658	101.1261 ± 95.4465	190.8313 ± 13.0957	172.0728 ± 35.1870	0.0614 ± 0.1193	173.1931 ± 35.8331	145.3740 ± 61.8658	149.6797 ± 64.6509	167.1639 ± 56.1163
POPT_L	103.5168 ± 92.4194	101.6116 ± 94.2752	139.4125 ± 62.5538	112.6716 ± 85.1496	0.0406 ± 0.0863	168.9418 ± 54.2808	112.7758 ± 71.7777	151.5154 ± 74.8438	139.6070 ± 82.1867
POPT_R	104.1734 ± 91.7846	101.5467 ± 94.3398	130.5875 ± 70.0885	109.0095 ± 87.9677	0.0772 ± 0.1639	153.1472 ± 69.1620	115.3940 ± 78.3786	143.2851 ± 77.3858	143.7801 ± 85.6768
PYT_L	102.3025 ± 93.9055	102.6709 ± 93.5808	120.0800 ± 78.3111	103.4642 ± 93.5588	0.0603 ± 0.1323	184.5577 ± 30.7148	122.8755 ± 69.1785	172.1689 ± 51.4240	146.3555 ± 86.6357
PYT_R	64.6197 ± 84.5313	64.6962 ± 84.4690	115.9903 ± 81.6593	100.7820 ± 93.9174	0.1166 ± 0.2115	183.6204 ± 28.7749	109.1597 ± 68.1185	166.9530 ± 55.9154	165.6398 ± 65.6945
SLF_L	8.7285 ± 6.9649	9.8758 ± 6.5081	121.5986 ± 64.2352	99.4938 ± 54.8821	0.1417 ± 0.1989	105.7062 ± 73.6827	90.4699 ± 62.7024	107.3434 ± 73.0325	91.4069 ± 80.8034
SLF_R	8.4198 ± 5.7724	10.3336 ± 6.9664	111.7213 ± 79.1217	97.2078 ± 66.3371	0.1896 ± 0.1996	119.8217 ± 61.5214	98.9296 ± 63.8060	102.2805 ± 66.3403	123.5613 ± 80.5927
UF_L	12.9460 ± 7.6095	22.0868 ± 14.5270	97.4400 ± 61.7695	73.8556 ± 48.6149	0.1220 ± 0.2109	119.0726 ± 85.2620	105.0600 ± 77.5498	113.5053 ± 86.5925	92.2117 ± 97.3248
UF_R	19.5961 ± 38.0511	20.9648 ± 38.1341	99.1578 ± 64.9586	72.3920 ± 53.4840	0.2013 ± 0.2121	103.5603 ± 76.2865	77.3140 ± 56.2050	94.6238 ± 78.2273	105.6646 ± 88.8256

Tab. S10 | Geometry and connectivity of bundles generated with RecobundlesX. Absolute percent differences are presented as mean ± standard deviation. A dash ″−′ indicates an absent bundle.

Bundles	Geometry APD (↓)				Pearson (↑)	Connectivity APD (↓)			
	Mean Length	Span	Volume	Surface Area		Density	CPL	Efficiency	Modularity
AF_L	6.8671 ± 5.1370	12.9578 ± 9.6855	80.4248 ± 31.5824	65.8603 ± 29.4192	0.5117 ± 0.1435	65.3658 ± 54.7625	72.8656 ± 45.7535	139.0153 ± 37.6715	113.2092 ± 59.4608
AF_R	6.2998 ± 5.2393	11.7393 ± 6.2468	110.7677 ± 43.0565	87.1237 ± 37.6548	0.4812 ± 0.1484	87.0591 ± 34.9837	59.9066 ± 41.0402	163.0339 ± 17.7238	105.9860 ± 60.0702
CC_Fr_1	15.3850 ± 5.3386	69.1439 ± 14.0994	52.5735 ± 29.4224	28.1593 ± 21.1176	0.2810 ± 0.0934	120.0259 ± 91.4889	36.0125 ± 56.2544	113.0662 ± 92.8543	86.7505 ± 98.9151
CC_Fr_2	6.7779 ± 4.2808	79.2540 ± 17.3526	34.3718 ± 26.8075	34.7595 ± 18.6882	0.3184 ± 0.3133	93.9333 ± 54.4729	85.7562 ± 61.1966	57.1404 ± 59.0518	125.5198 ± 86.6299
CC_Oc	5.8269 ± 4.1057	26.1639 ± 11.7008	31.4334 ± 19.6175	34.0720 ± 15.8905	0.3774 ± 0.1972	55.4983 ± 42.8273	45.2238 ± 29.1175	75.1446 ± 36.5914	177.0998 ± 45.6462
CC_Pa	5.0622 ± 4.3203	69.1572 ± 13.2717	24.1413 ± 17.6771	39.7097 ± 23.2796	0.4106 ± 0.1169	43.7868 ± 16.2084	60.3170 ± 16.2961	73.3030 ± 24.1981	162.7212 ± 69.0877
CC_Pr_Po	7.2905 ± 3.6814	76.4518 ± 15.2194	20.2895 ± 10.3475	19.0045 ± 12.5899	0.4542 ± 0.1735	62.5391 ± 36.8505	52.4721 ± 36.9441	68.2240 ± 38.0621	165.5790 ± 65.4351
CG_L	9.3606 ± 7.1440	7.1445 ± 5.4248	65.1877 ± 32.5148	54.4920 ± 26.2758	0.3232 ± 0.2259	68.1916 ± 43.3152	73.1483 ± 58.6951	154.2046 ± 28.2237	120.0022 ± 74.5257
CG_R	7.5502 ± 4.7267	5.5390 ± 3.7249	70.8563 ± 28.5890	57.4128 ± 24.9052	0.1967 ± 0.1724	88.1679 ± 41.3946	79.2521 ± 61.9854	165.4979 ± 21.3220	169.8746 ± 51.8653
FAT_L	21.2143 ± 4.4211	27.4959 ± 4.9851	61.4267 ± 22.5394	40.8125 ± 15.7842	0.2899 ± 0.1970	147.8708 ± 65.1646	114.3985 ± 58.8828	131.7010 ± 76.2037	57.5395 ± 86.2745
FAT_R	18.0987 ± 6.1302	22.9826 ± 8.6943	47.2526 ± 22.3038	33.9039 ± 18.4856	0.3415 ± 0.1170	136.1283 ± 66.4507	111.4051 ± 59.7472	112.0758 ± 80.1887	117.4574 ± 98.2727
FPT_L	6.4878 ± 4.9733	5.1643 ± 5.2063	28.3680 ± 19.1025	24.6312 ± 15.9789	0.2449 ± 0.2240	140.3164 ± 72.0895	68.1552 ± 63.2570	132.9945 ± 69.4190	124.9392 ± 96.7776
FPT_R	5.3322 ± 4.2567	4.8863 ± 5.2827	30.3603 ± 20.3129	20.4533 ± 16.1621	0.2556 ± 0.2291	134.5413 ± 66.5576	70.5679 ± 61.7485	114.2451 ± 75.5957	149.5326 ± 82.3574
IFOF_L	5.5881 ± 4.1287	9.4986 ± 5.5994	28.1587 ± 19.2804	18.2430 ± 10.9580	0.4457 ± 0.1790	63.6361 ± 30.7008	46.5834 ± 40.0148	69.5504 ± 37.9557	139.7234 ± 64.4818
IFOF_R	7.7613 ± 5.6856	13.3749 ± 8.2729	24.2648 ± 15.8628	14.9966 ± 9.9397	0.5059 ± 0.1928	70.2414 ± 38.9035	56.6767 ± 46.5375	72.2860 ± 42.7081	131.4996 ± 70.1214
ILF_L	7.8329 ± 4.6974	5.2944 ± 3.6101	29.3315 ± 26.3861	23.8331 ± 17.6178	0.2635 ± 0.1469	54.2988 ± 49.7338	56.0230 ± 40.7227	80.4280 ± 48.8916	120.3754 ± 68.8230
ILF_R	5.1991 ± 4.1538	6.1044 ± 2.7182	24.9717 ± 21.1794	17.5200 ± 14.3292	0.2726 ± 0.1521	38.4853 ± 27.7240	50.6188 ± 30.0618	90.4429 ± 29.0305	127.2172 ± 74.1863
MCP	10.5857 ± 6.3202	17.3298 ± 11.1574	51.4537 ± 30.1220	43.3855 ± 33.6452	-	-	-	-	-
MdLF_L	14.3665 ± 8.3194	8.4157 ± 5.5276	56.8422 ± 49.5838	36.7739 ± 31.1822	0.4163 ± 0.1573	50.8289 ± 30.1481	61.8143 ± 23.6356	97.4048 ± 46.8142	125.1156 ± 66.5788
MdLF_R	10.7450 ± 7.9964	8.2246 ± 5.9636	55.3305 ± 50.2630	36.1239 ± 34.6153	0.4632 ± 0.1531	45.8801 ± 36.1392	71.4388 ± 28.5265	104.3784 ± 42.4640	135.6276 ± 68.1296
OR_ML_L	4.1400 ± 3.8627	10.0678 ± 5.7946	59.0603 ± 31.4590	42.5010 ± 24.2010	0.3573 ± 0.1703	45.0228 ± 31.4545	59.9386 ± 31.6911	95.1105 ± 36.5221	88.5938 ± 69.5327
OR_ML_R	5.5102 ± 7.1274	12.7676 ± 7.6223	86.4971 ± 42.1039	62.5457 ± 34.9648	0.2138 ± 0.1196	47.8760 ± 35.7274	53.2716 ± 34.1940	111.9585 ± 39.9669	69.9509 ± 58.2121
POPT_L	4.5857 ± 3.1448	5.1290 ± 3.8614	23.6069 ± 13.4042	21.2768 ± 11.9264	0.2908 ± 0.2180	144.0642 ± 64.5311	99.7508 ± 63.4215	121.5503 ± 77.3041	126.2808 ± 88.1962
POPT_R	4.4651 ± 3.0735	5.7789 ± 4.2919	26.3258 ± 19.7795	22.0600 ± 14.7985	0.3237 ± 0.1824	125.5377 ± 67.2363	113.6394 ± 65.2036	99.3050 ± 75.7996	153.7215 ± 75.8021
PYT_L	3.8525 ± 3.7411	6.0467 ± 3.6747	63.3981 ± 21.2531	36.9747 ± 14.4668	0.4169 ± 0.2487	165.0297 ± 48.0601	99.1033 ± 68.9259	145.0567 ± 67.5981	146.3560 ± 83.8815
PYT_R	3.6086 ± 3.4015	5.9533 ± 3.4104	63.3580 ± 17.7112	35.7914 ± 12.2064	0.3720 ± 0.2331	166.4927 ± 38.0673	79.4674 ± 65.5698	133.1660 ± 68.2547	155.3007 ± 75.5170
SLF_L	11.0423 ± 7.2426	9.7943 ± 7.3305	96.2303 ± 43.7705	66.0393 ± 36.0735	0.4064 ± 0.1902	79.7213 ± 77.7249	65.8042 ± 49.2349	152.9229 ± 33.4092	121.1710 ± 75.6789
SLF_R	6.9994 ± 5.5779	11.6982 ± 6.1827	68.6048 ± 47.6555	47.0659 ± 33.1078	0.5068 ± 0.1499	53.3537 ± 31.5136	74.2599 ± 49.0805	113.8505 ± 43.8950	126.6906 ± 70.0115
UF_L	12.1606 ± 8.3958	14.4482 ± 11.9936	77.7074 ± 49.4321	43.2770 ± 33.8591	0.4197 ± 0.1874	36.2327 ± 46.3920	62.0342 ± 59.2523	61.0680 ± 57.6517	108.2708 ± 90.1743
UF_R	5.9925 ± 4.5454	13.8315 ± 10.2998	59.7495 ± 47.9775	36.6240 ± 34.8001	0.3359 ± 0.2197	69.6248 ± 57.9120	76.1830 ± 57.7207	62.0682 ± 57.6067	122.7808 ± 84.0423

Tab. S11 | Geometry and connectivity of bundles generated with TractSeg. Absolute percent differences are presented as mean ± standard deviation. A dash ′′−′ indicates an absent bundle.

Bundles	Geometry APD (↓)				Pearson (↑)	Connectivity APD (↓)			
	Mean Length	Span	Volume	Surface Area		Density	CPL	Efficiency	Modularity
AF_L	108.6242 ± 87.9049	104.5701 ± 92.0085	107.0878 ± 89.9502	110.2794 ± 87.6586	0.3509 ± 0.1771	129.2319 ± 70.9613	149.4215 ± 50.2974	163.1522 ± 43.6546	150.3046 ± 65.7166
AF_R	102.0625 ± 94.2559	102.0564 ± 94.2713	127.9537 ± 72.7292	120.8015 ± 78.6481	0.3988 ± 0.1670	124.0621 ± 78.8953	137.9303 ± 60.5412	167.0533 ± 36.3612	160.9371 ± 60.8781
CG_L	109.1932 ± 91.4129	107.3552 ± 93.0713	128.1646 ± 74.1421	126.0817 ± 75.6344	0.3249 ± 0.1296	134.1335 ± 74.4936	135.8587 ± 73.2987	169.6590 ± 37.3033	140.5862 ± 83.9344
CG_R	110.4426 ± 89.8437	107.4991 ± 92.7528	129.2591 ± 73.6956	121.9690 ± 79.7620	0.3087 ± 0.1261	140.7709 ± 65.4758	128.4395 ± 70.5760	179.6932 ± 23.0767	135.9604 ± 83.0127
FPT_L	114.6889 ± 92.8783	112.8239 ± 94.9221	130.0743 ± 78.9041	131.0289 ± 76.8399	0.1680 ± 0.2294	158.1861 ± 62.8069	137.3441 ± 58.1106	160.4339 ± 62.3067	116.6060 ± 98.5502
FPT_R	114.7110 ± 92.8800	113.0721 ± 94.7072	128.4543 ± 80.4207	132.2092 ± 75.6787	0.2598 ± 0.1107	152.0733 ± 74.8396	127.1801 ± 54.7976	165.6695 ± 56.8184	151.3615 ± 84.3869
IFOF_L	150.4790 ± 78.3311	148.9504 ± 80.7514	163.4804 ± 58.9076	157.9958 ± 67.4280	0.5757 ± 0.1183	171.9733 ± 46.1978	174.9170 ± 45.9423	187.9807 ± 18.0823	169.2243 ± 62.2790
IFOF_R	149.3084 ± 80.1683	147.8268 ± 82.5703	159.5955 ± 64.1054	155.4699 ± 70.6078	0.6215 ± 0.0737	167.2004 ± 51.6859	189.5736 ± 8.3633	188.7635 ± 14.1480	176.3352 ± 56.9079
ILF_L	94.9607 ± 92.3280	93.0919 ± 93.8667	136.2910 ± 63.6383	138.0434 ± 59.6729	0.2580 ± 0.1279	138.3339 ± 70.9965	116.2617 ± 70.4294	142.8129 ± 66.1975	113.2351 ± 90.4153
ILF_R	96.6653 ± 90.7001	93.9635 ± 93.0601	141.0087 ± 55.4564	142.6515 ± 52.7193	0.2536 ± 0.1253	143.5662 ± 62.8942	121.5727 ± 70.7468	134.3512 ± 74.1819	114.9235 ± 86.6453
MCP	64.7858 ± 44.9386	36.8547 ± 28.5301	89.8914 ± 66.1316	77.8534 ± 66.3309	-	-	-	-	-
MdLF_L	88.8504 ± 77.5839	105.9543 ± 80.7572	139.0497 ± 68.8690	141.4967 ± 62.9488	0.1744 ± 0.1748	139.2102 ± 59.7829	121.2641 ± 65.3709	168.6524 ± 32.4812	166.3795 ± 50.8442
MdLF_R	106.5477 ± 87.6184	110.4615 ± 89.1413	139.7272 ± 72.6503	143.0937 ± 63.3809	0.2992 ± 0.1727	132.1819 ± 71.2985	122.3044 ± 73.8374	166.2555 ± 36.9366	150.1958 ± 71.9453
POPT_L	103.1964 ± 92.8150	103.2093 ± 92.8561	118.8870 ± 79.3817	113.6052 ± 83.6849	0.1577 ± 0.1431	134.2133 ± 75.4831	157.0062 ± 53.0696	156.9101 ± 57.5588	146.7786 ± 72.4481
POPT_R	103.2112 ± 92.7266	103.3183 ± 92.6293	115.9655 ± 82.6732	114.5515 ± 83.1277	0.0783 ± 0.0640	135.1283 ± 77.5757	176.1081 ± 20.6275	156.0065 ± 59.6288	140.9632 ± 67.7352
UF_L	119.6257 ± 74.4698	119.2909 ± 75.2950	122.5388 ± 75.9213	135.1954 ± 64.7735	0.2849 ± 0.1600	109.7875 ± 90.2565	115.5420 ± 80.6354	114.3156 ± 88.1505	74.8395 ± 92.6888
UF_R	117.8611 ± 76.2943	117.4922 ± 76.4676	125.1935 ± 73.7242	135.9309 ± 63.7862	0.3391 ± 0.2352	114.0819 ± 85.8847	115.6618 ± 78.5836	132.0010 ± 72.9096	112.2812 ± 88.3343

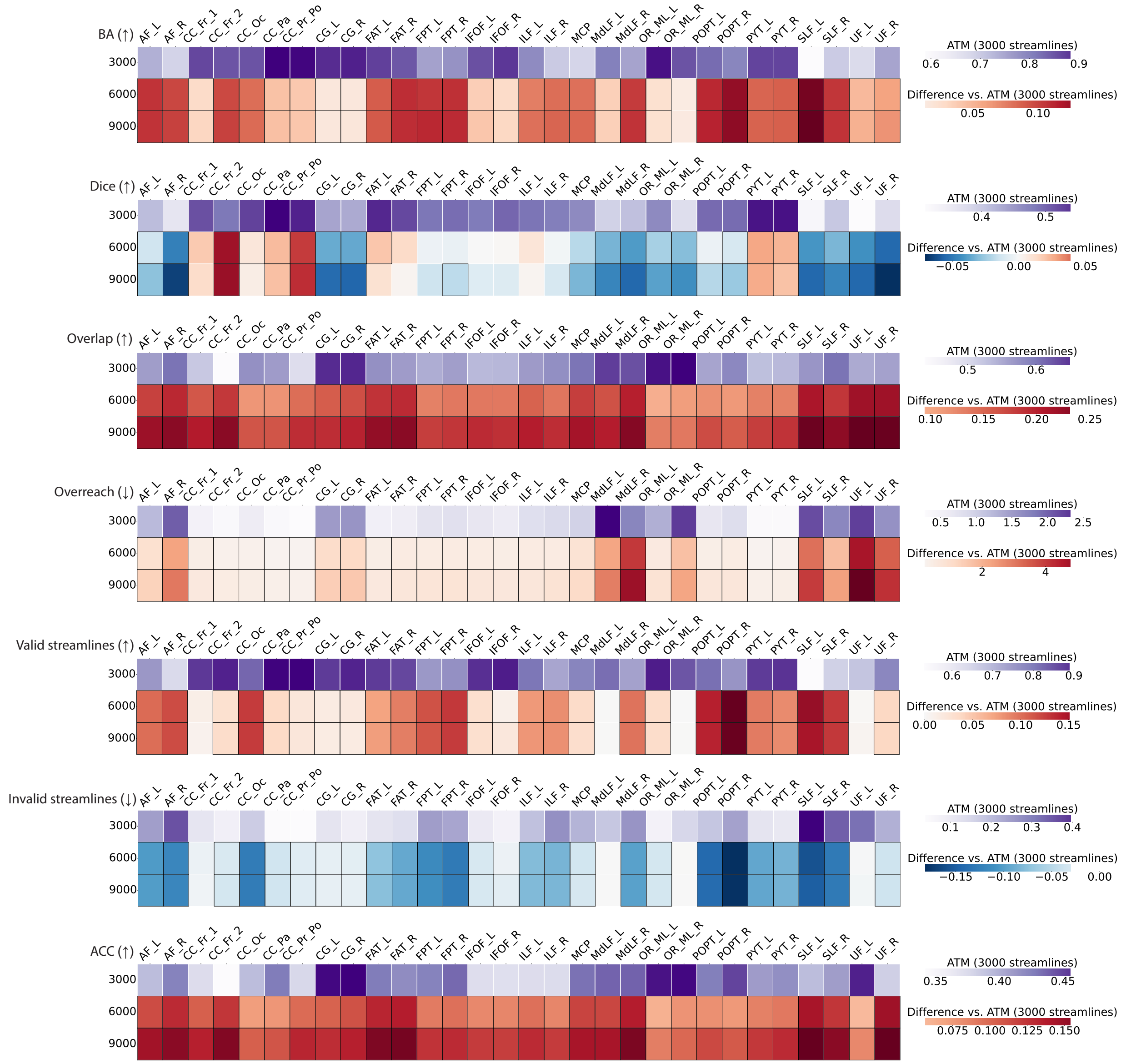

Fig. S9 | Similarity and coverage of bundles generated with ATM using 3000, 6000, and 9000 streamlines per bundle. The first row of each heatmap shows the values for 3,000 streamlines per bundle as the baseline. The subsequent rows display the paired performance differences of ATM with other streamline counts relative to this baseline. The arrow indicates preference: ↑ higher is better, ↓ lower is better. Cells outlined with squares indicate statistically significant differences.

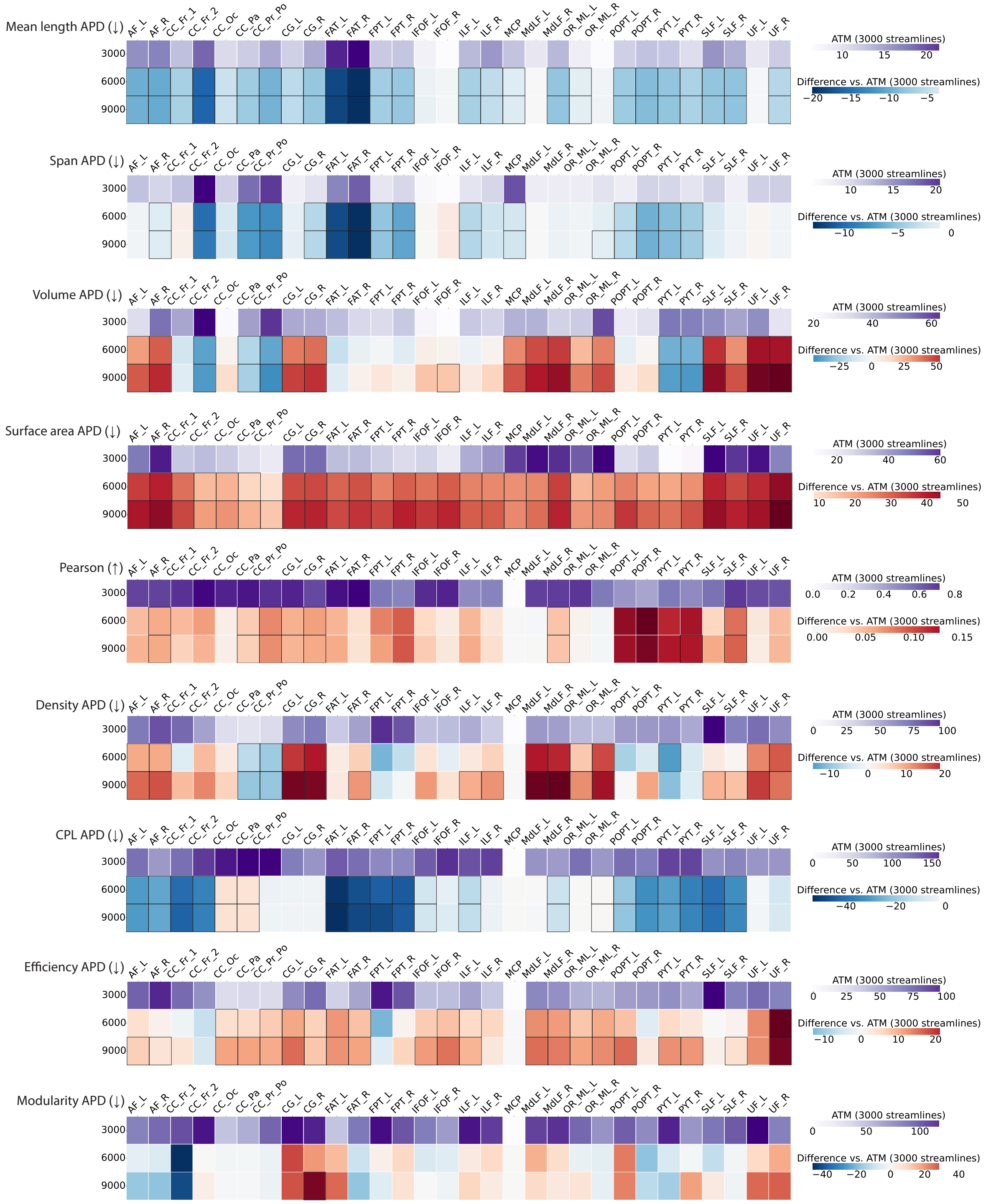

Fig. S10 | Geometry and connectivity of bundles generated with ATM using 3,000, 6,000, and 9,000 streamlines per bundle. The first row of each heatmap shows the values for 3,000 streamlines per bundle as the baseline. The subsequent rows display the paired performance differences of ATM with other streamline counts relative to this baseline. The arrow indicates preference: ↑ higher is better, ↓ lower is better. Cells outlined with squares indicate statistically significant differences.

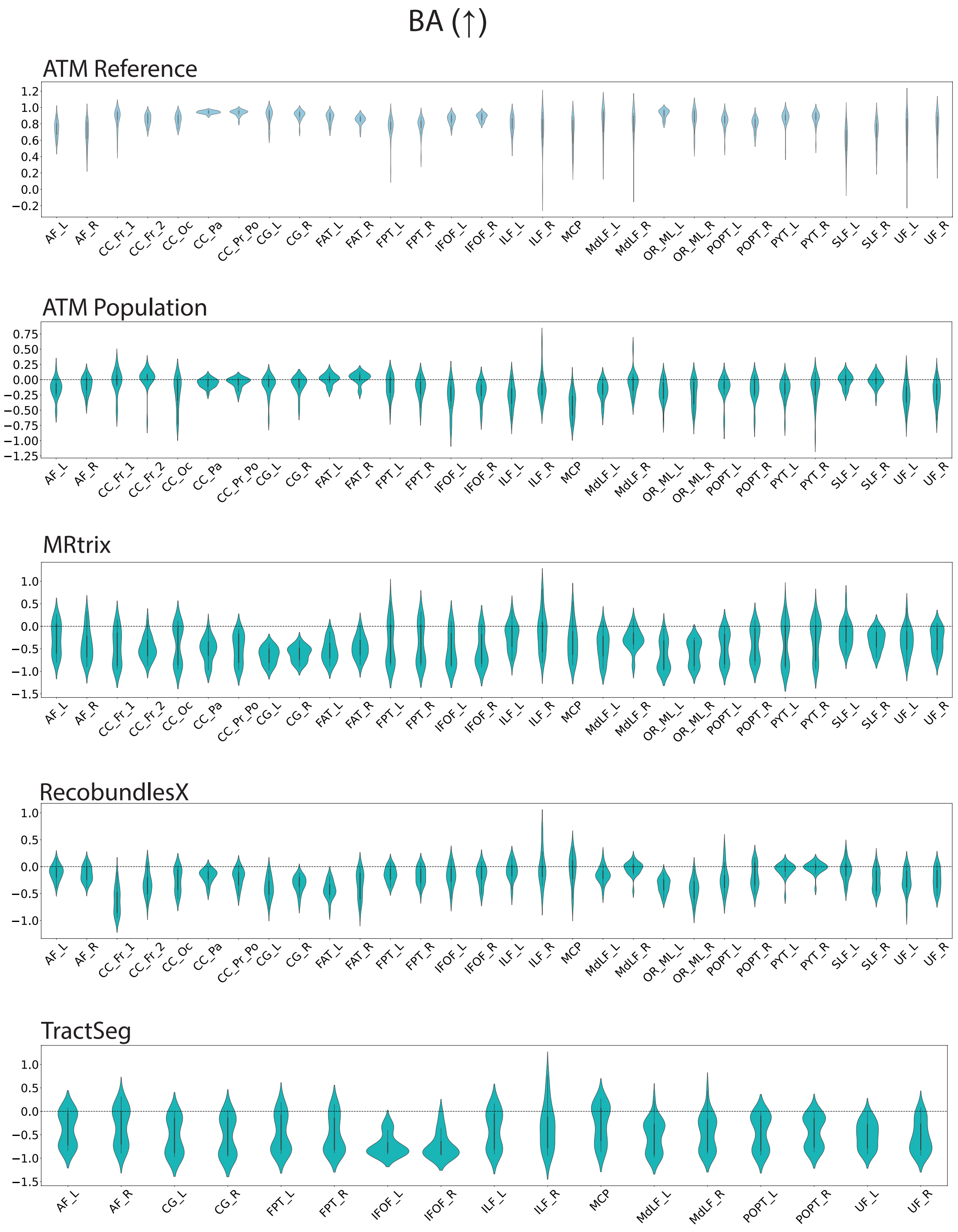

Fig. S11 | BA of bundles generated with ATM, ATM Population, MRtrix, RecobundlesX, and TractSeg. The first violin plot shows the ATM values as the baseline. The subsequent rows display the paired performance differences of other methods relative to this baseline. For each metric, the arrow indicates preference: ↑ higher is better and ↓ lower is better.

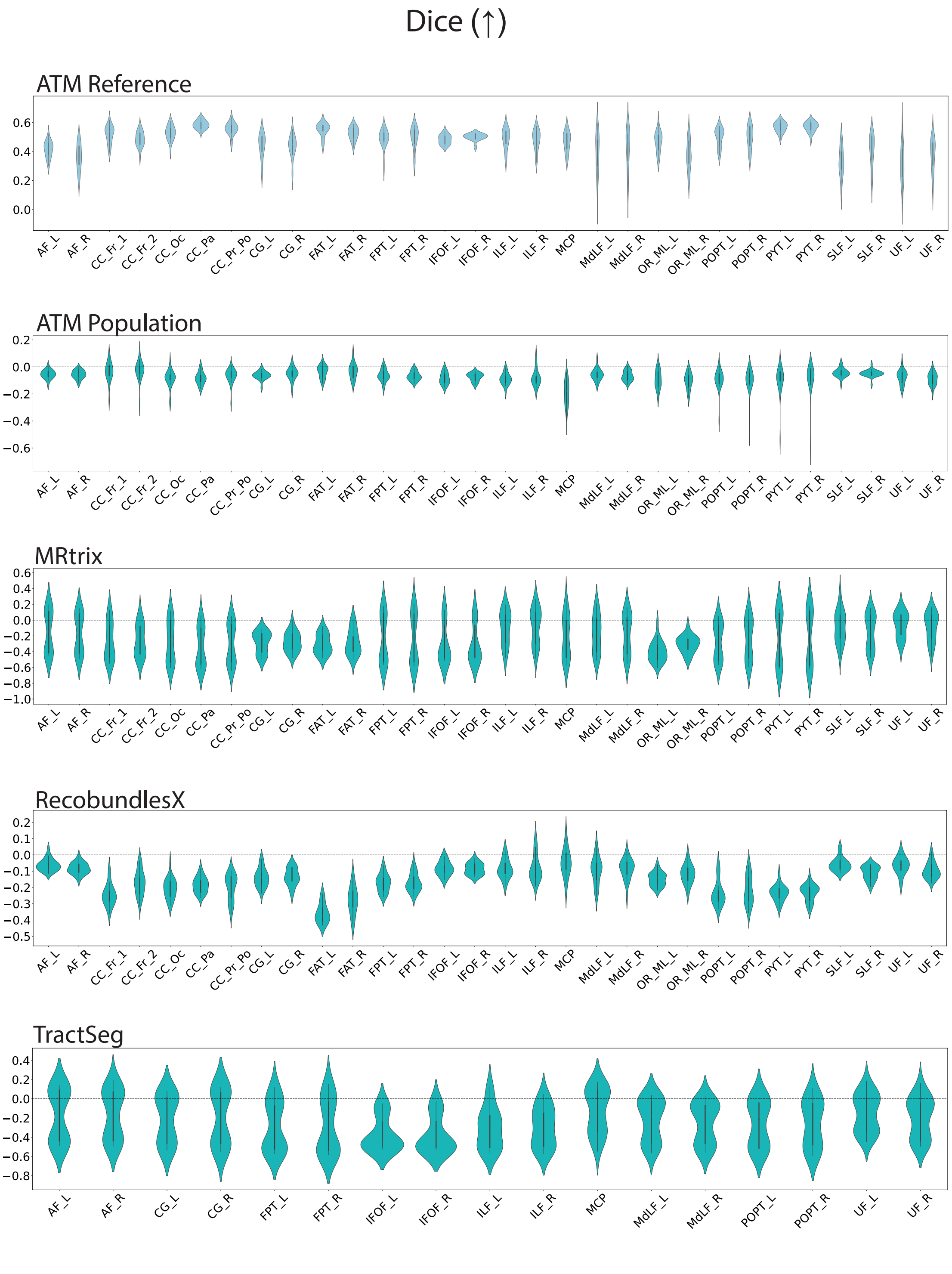

Fig. S12 | Dice scores of bundles generated with ATM, ATM Population, MRtrix, RecobundlesX, and TractSeg. The first violin plot shows the ATM values as the baseline. The subsequent rows display the paired performance differences of other methods relative to this baseline. For each metric, the arrow indicates preference: ↑ higher is better and ↓ lower is better.

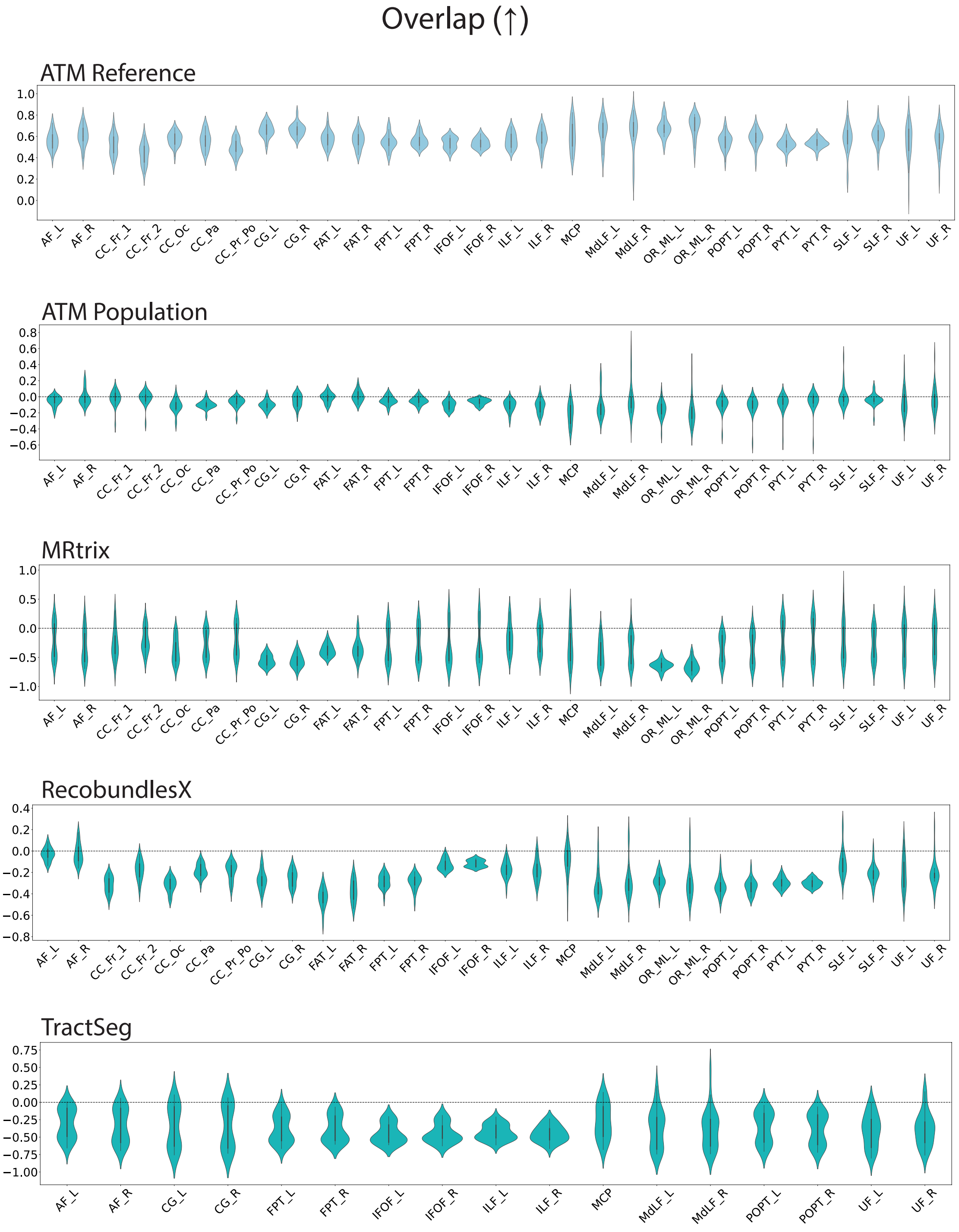

Fig. S13 | Overlap of bundles generated with ATM, ATM Population, MRtrix, RecobundlesX, and TractSeg. The first violin plot shows the ATM values as the baseline. The subsequent rows display the paired performance differences of other methods relative to this baseline. For each metric, the arrow indicates preference: ↑ higher is better and ↓ lower is better.

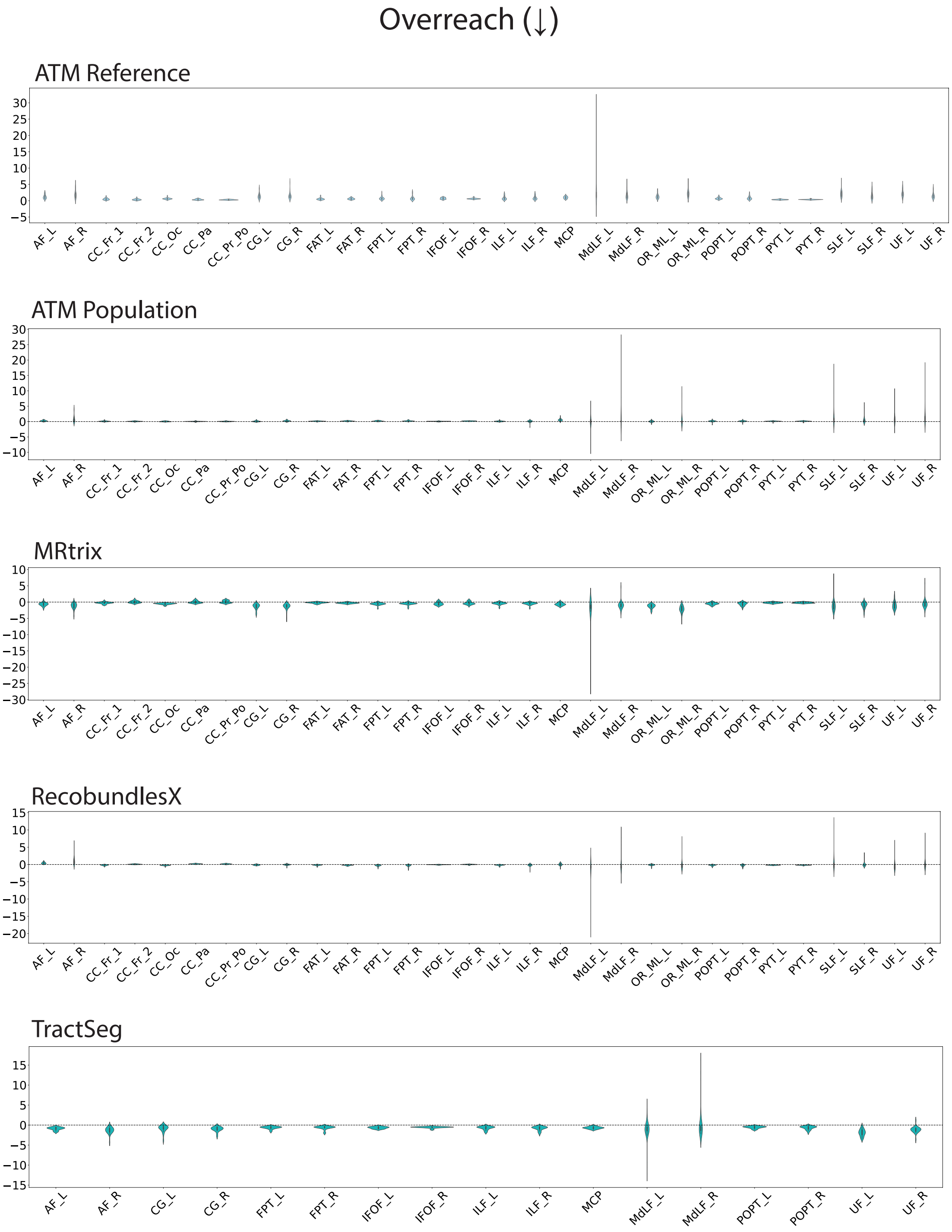

Fig. S14 | Overreach of bundles generated with ATM, ATM Population, MRtrix, RecobundlesX, and TractSeg. The first violin plot shows the ATM values as the baseline. The subsequent rows display the paired performance differences of other methods relative to this baseline. For each metric, the arrow indicates preference: ↑ higher is better and ↓ lower is better.

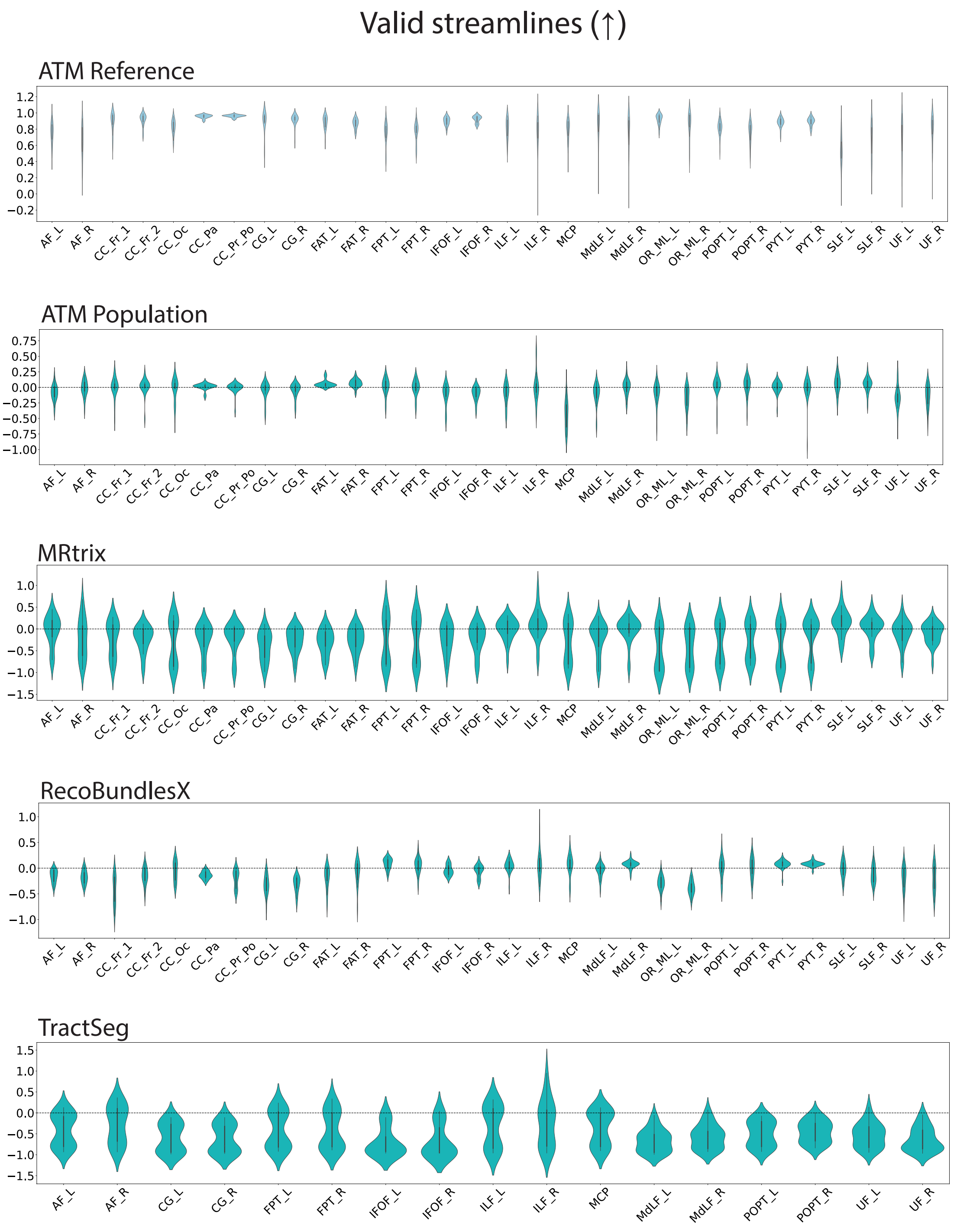

Fig. S15 | Ratio of valid streamlines in bundles generated with ATM, ATM Population, MRtrix, RecobundlesX, and TractSeg. The first violin plot shows the ATM values as the baseline. The subsequent rows display the paired performance differences of other methods relative to this baseline. For each metric, the arrow indicates preference: ↑ higher is better and ↓ lower is better.

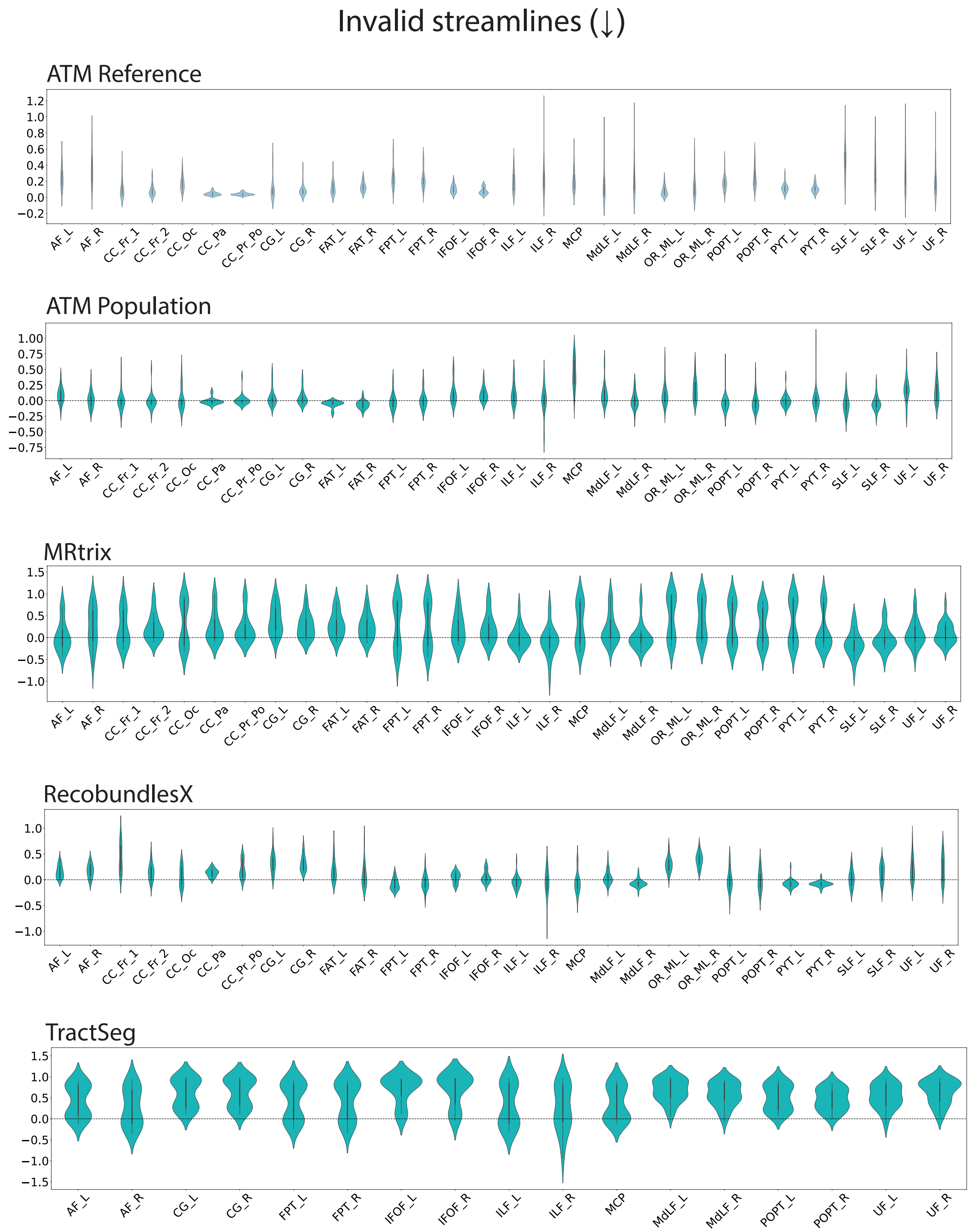

Fig. S16 | Ratio of invalid streamlines in bundles generated with ATM, ATM Population, MRtrix, Recobundles, and TractSeg. The first violin plot shows the ATM values as the baseline. The subsequent rows display the paired performance differences of other methods relative to this baseline. For each metric, the arrow indicates preference: ↑ higher is better and ↓ lower is better.

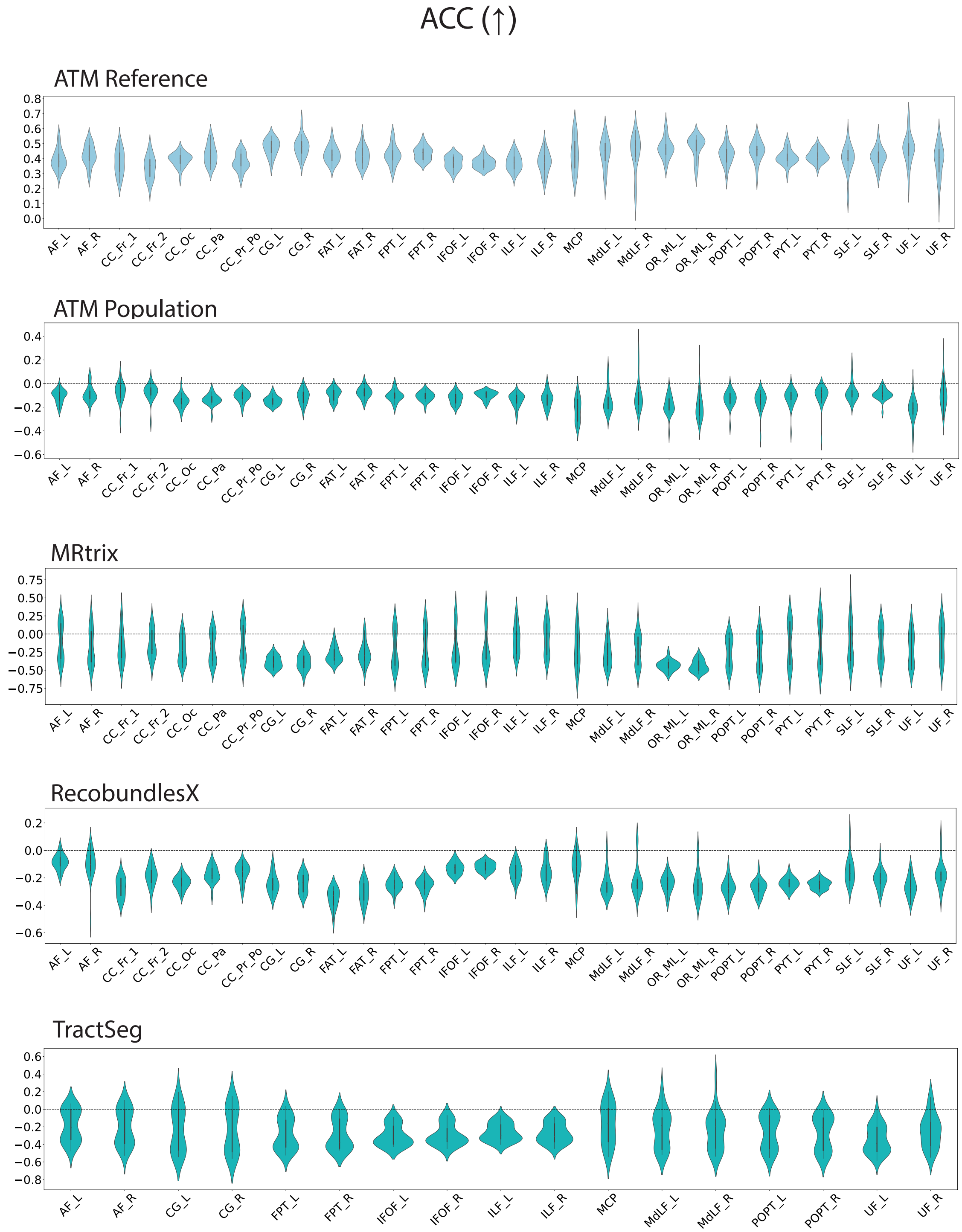

Fig. S17 | Angular correlation coefficient of bundles generated with ATM, ATM Population, MRtrix, RecobundlesX, and TractSeg. The first violin plot shows the ATM values as the baseline. The subsequent rows display the paired performance differences of other methods relative to this baseline. For each metric, the arrow indicates preference: ↑ higher is better and ↓ lower is better.

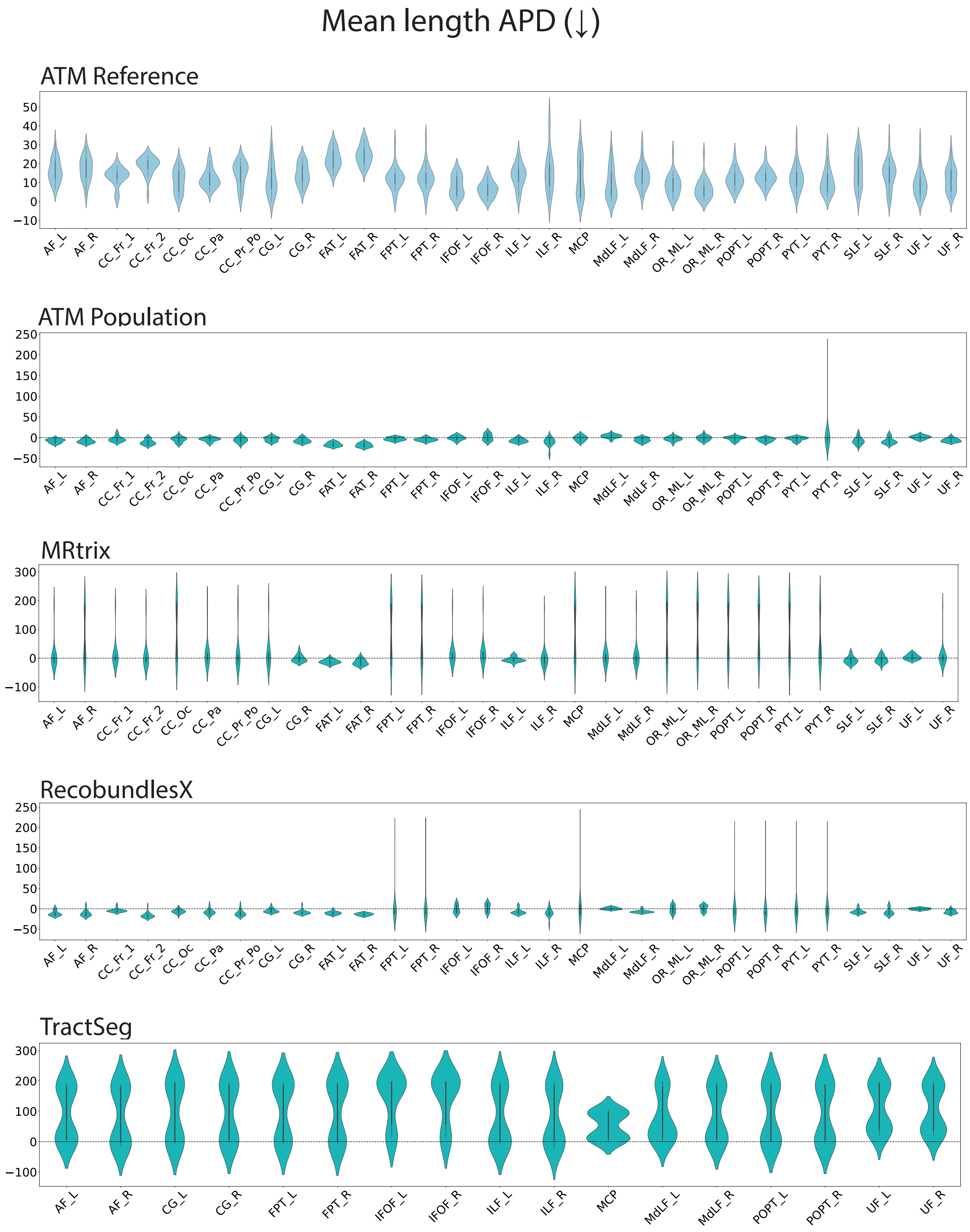

Fig. S18 | Mean length APD of bundles generated with ATM, ATM Population, MRtrix, RecobundlesX, and TractSeg. The first violin plot shows the ATM values as the baseline. The subsequent rows display the paired performance differences of other methods relative to this baseline. For each metric, the arrow indicates preference: ↑ higher is better and ↓ lower is better.

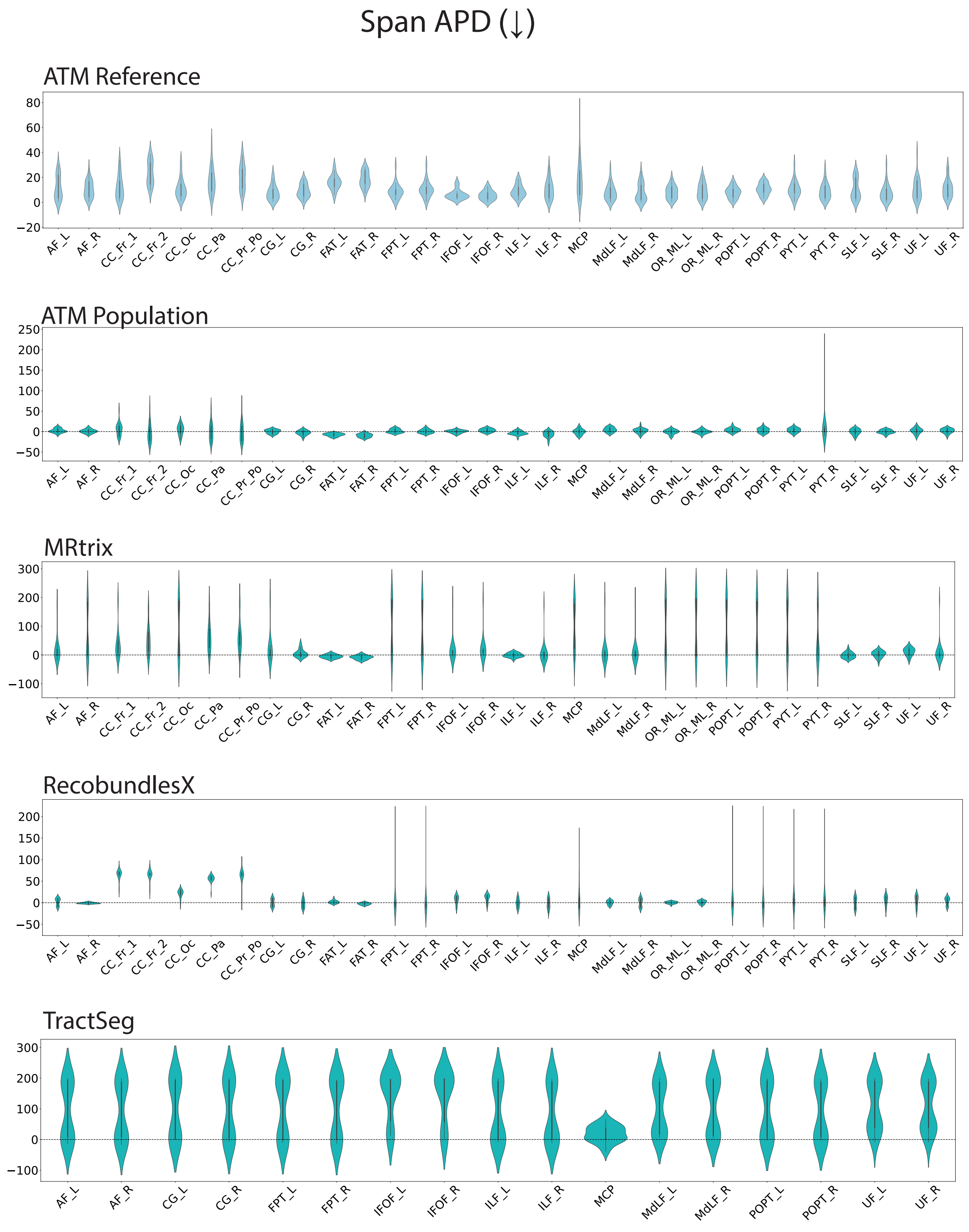

Fig. S19 | Span APD of bundles generated with ATM, ATM Population, MRtrix, RecobundlesX, and TractSeg. The first violin plot shows the ATM values as the baseline. The subsequent rows display the paired performance differences of other methods relative to this baseline. For each metric, the arrow indicates preference: ↑ higher is better and ↓ lower is better.

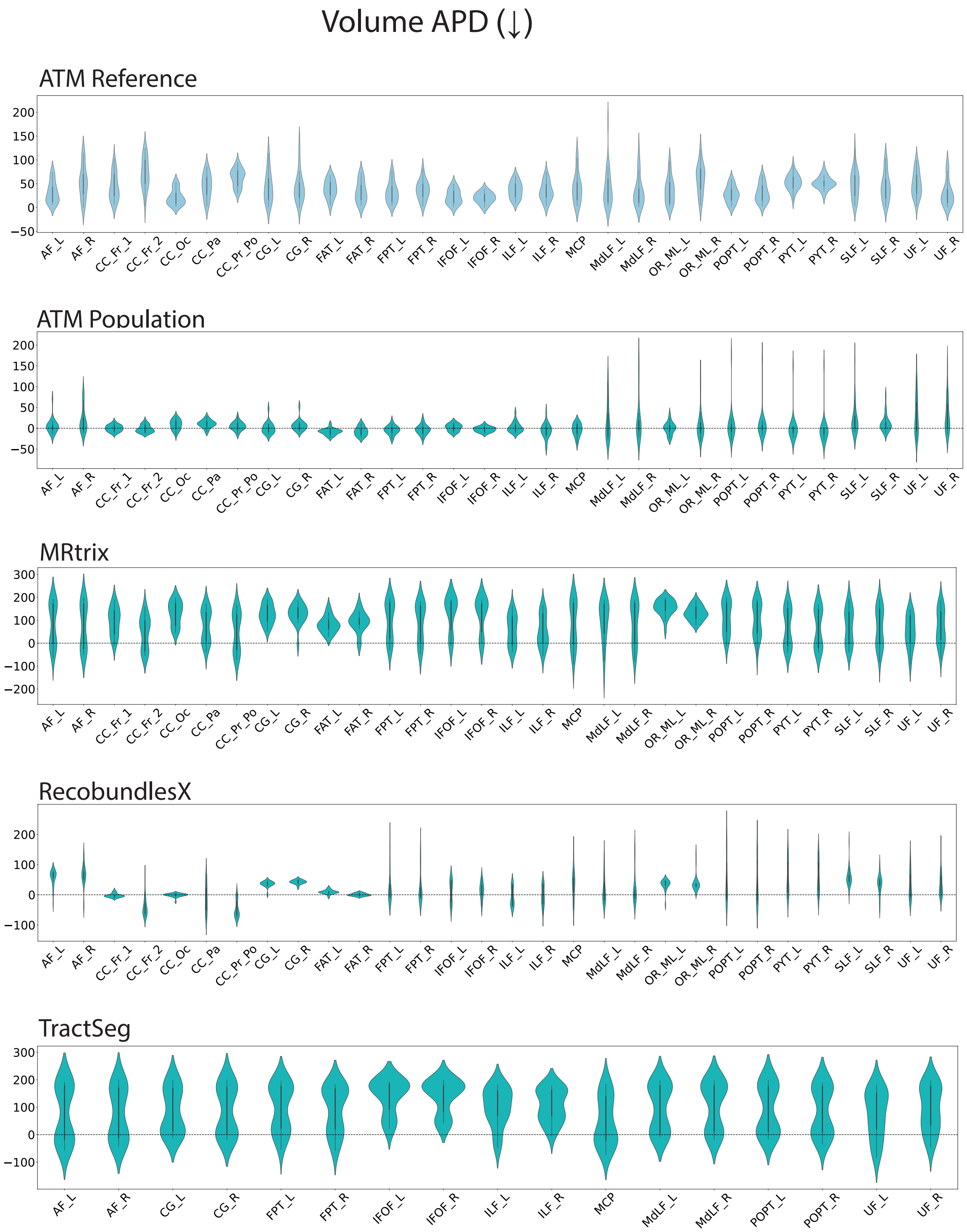

Fig. S20 | Volume APD of bundles generated with ATM, ATM Population, MRtrix, RecobundlesX, and TractSeg. The first violin plot shows the ATM values as the baseline. The subsequent rows display the paired performance differences of other methods relative to this baseline. For each metric, the arrow indicates preference: ↑ higher is better and ↓ lower is better.

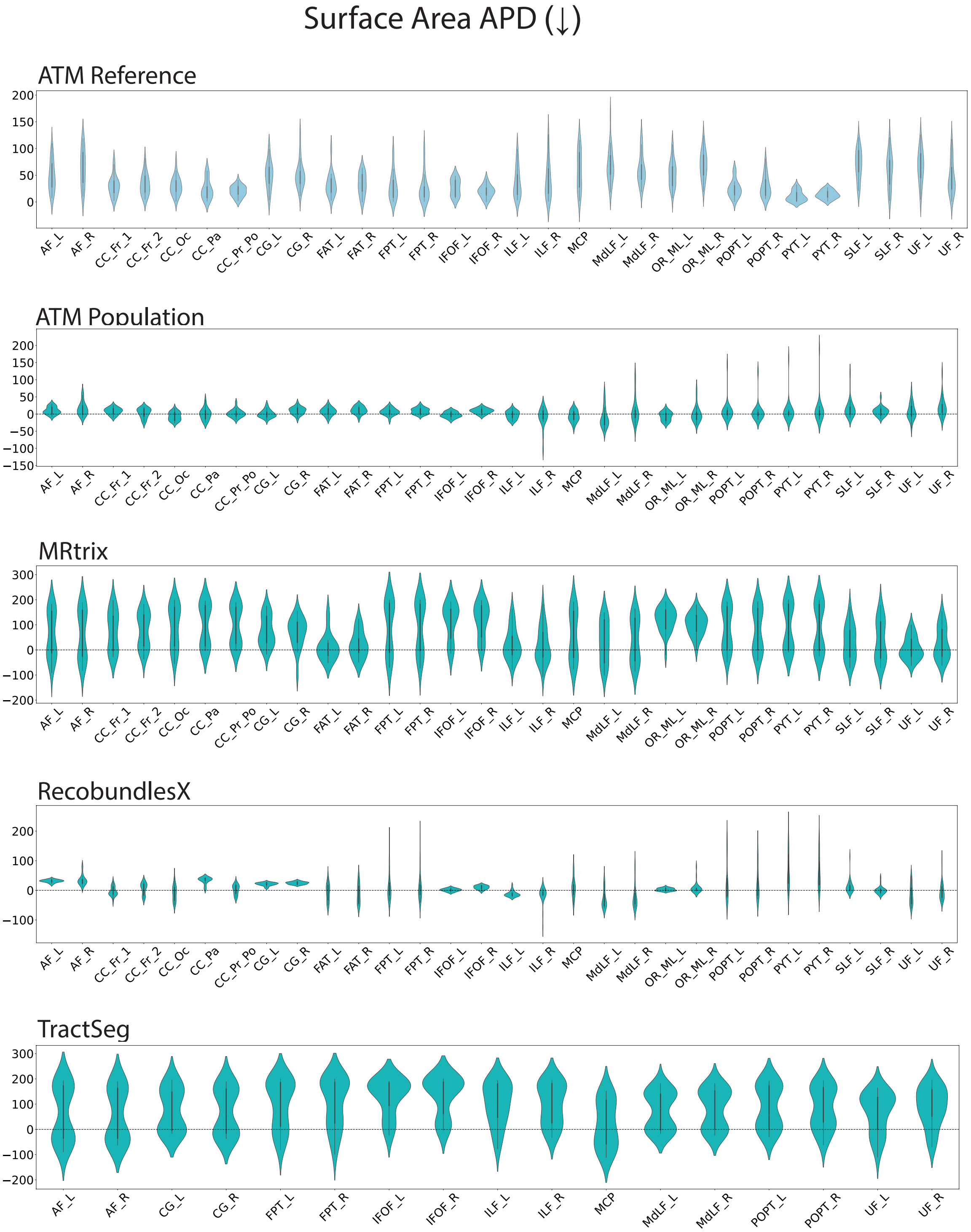

Fig. S21 | Surface area APD of bundles generated with ATM, ATM Population, MRtrix, RecobundlesX, and TractSeg. The first violin plot shows the ATM values as the baseline. The subsequent rows display the paired performance differences of other methods relative to this baseline. For each metric, the arrow indicates preference: ↑ higher is better and ↓ lower is better.

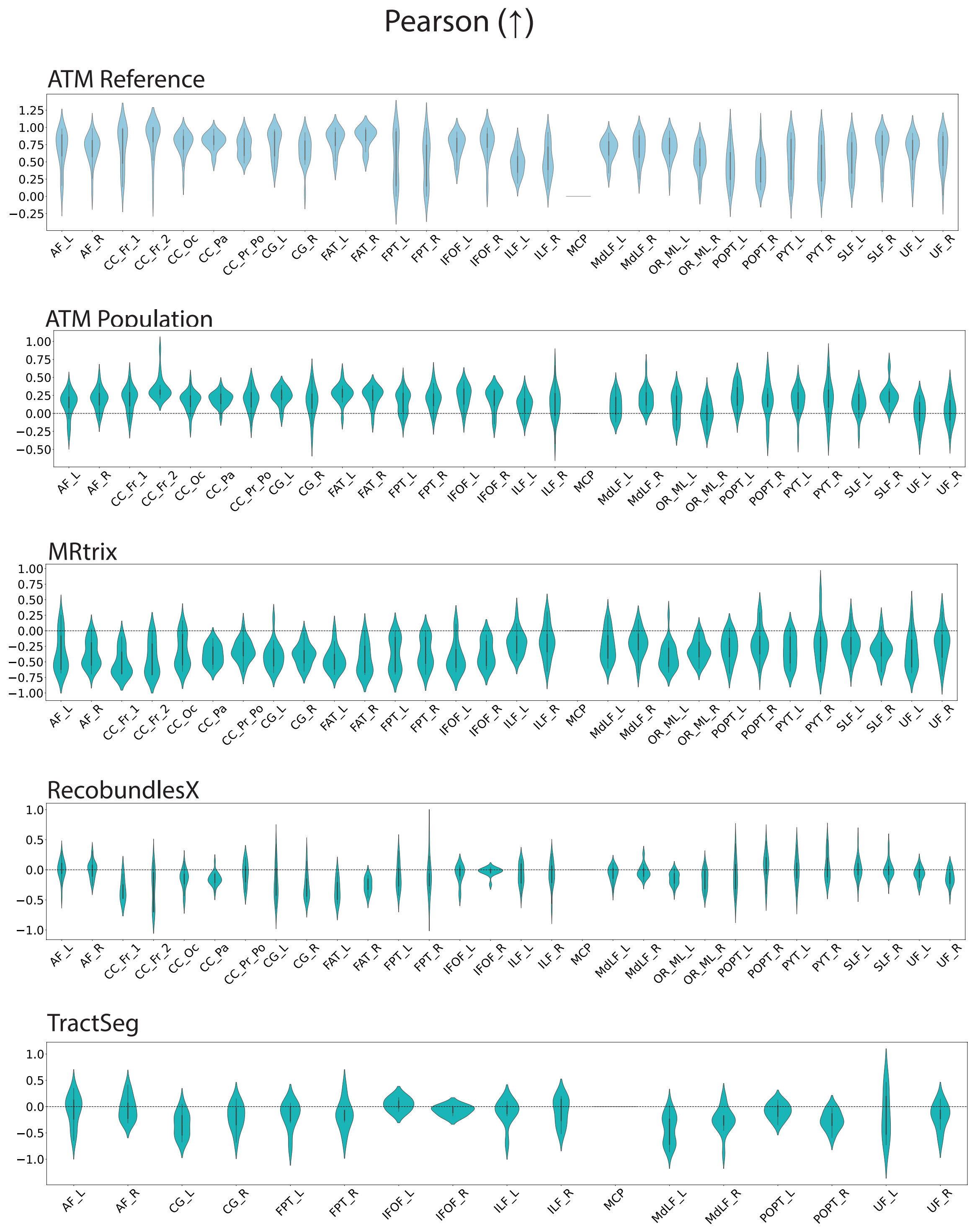

Fig. S22 | Pearson corerlation of bundle connectomes generated with ATM, ATM Population, MRtrix, RecobundlesX, and TractSeg. The first violin plot shows the ATM values as the baseline. The subsequent rows display the paired performance differences of other methods relative to this baseline. For each metric, the arrow indicates preference: ↑ higher is better and ↓ lower is better.

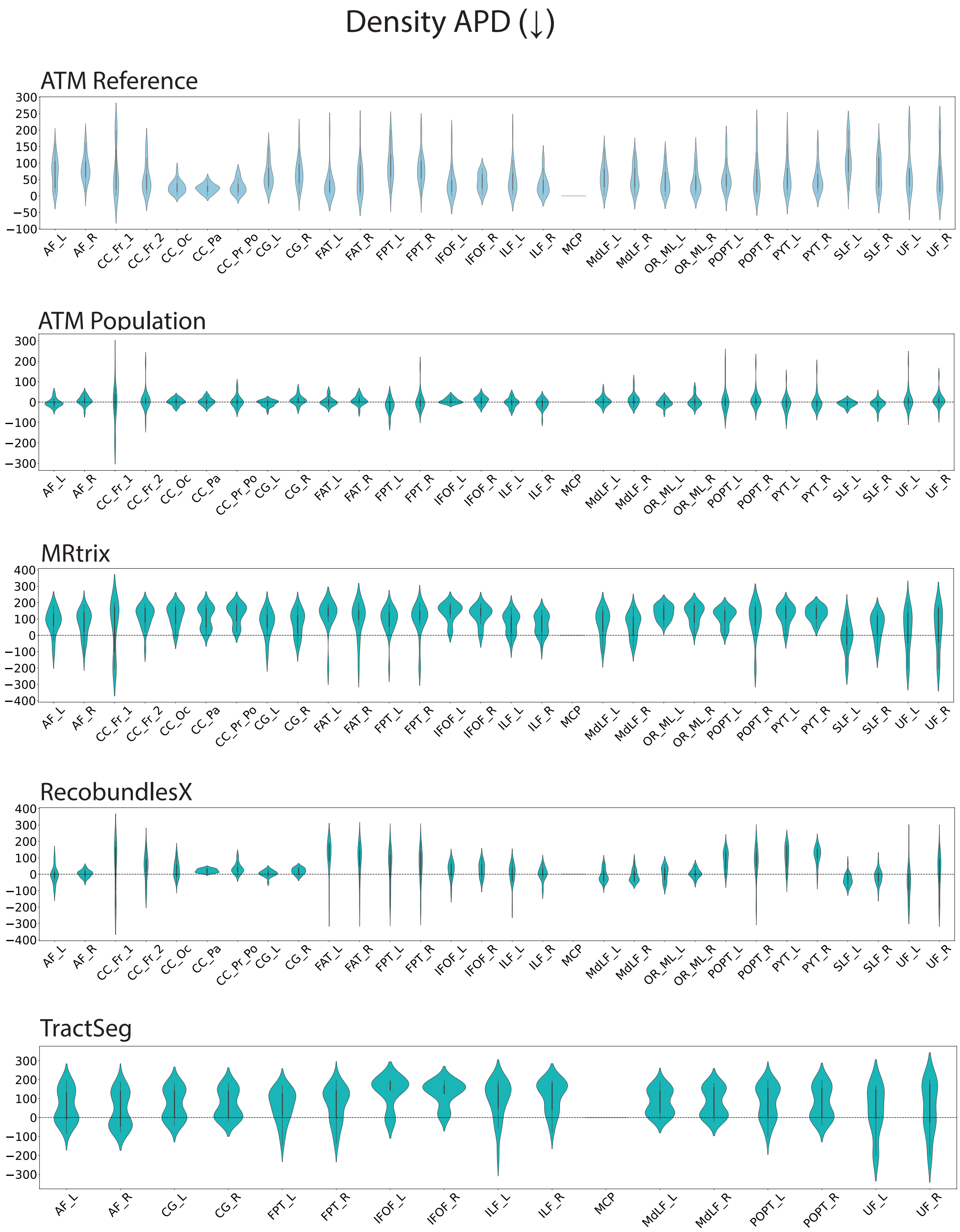

Fig. S23 | Network density APD of bundle connectomes generated with ATM, ATM Population, MRtrix, RecobundlesX, and TractSeg. The first violin plot shows the ATM values as the baseline. The subsequent rows display the paired performance differences of other methods relative to this baseline. For each metric, the arrow indicates preference: ↑ higher is better and ↓ lower is better.

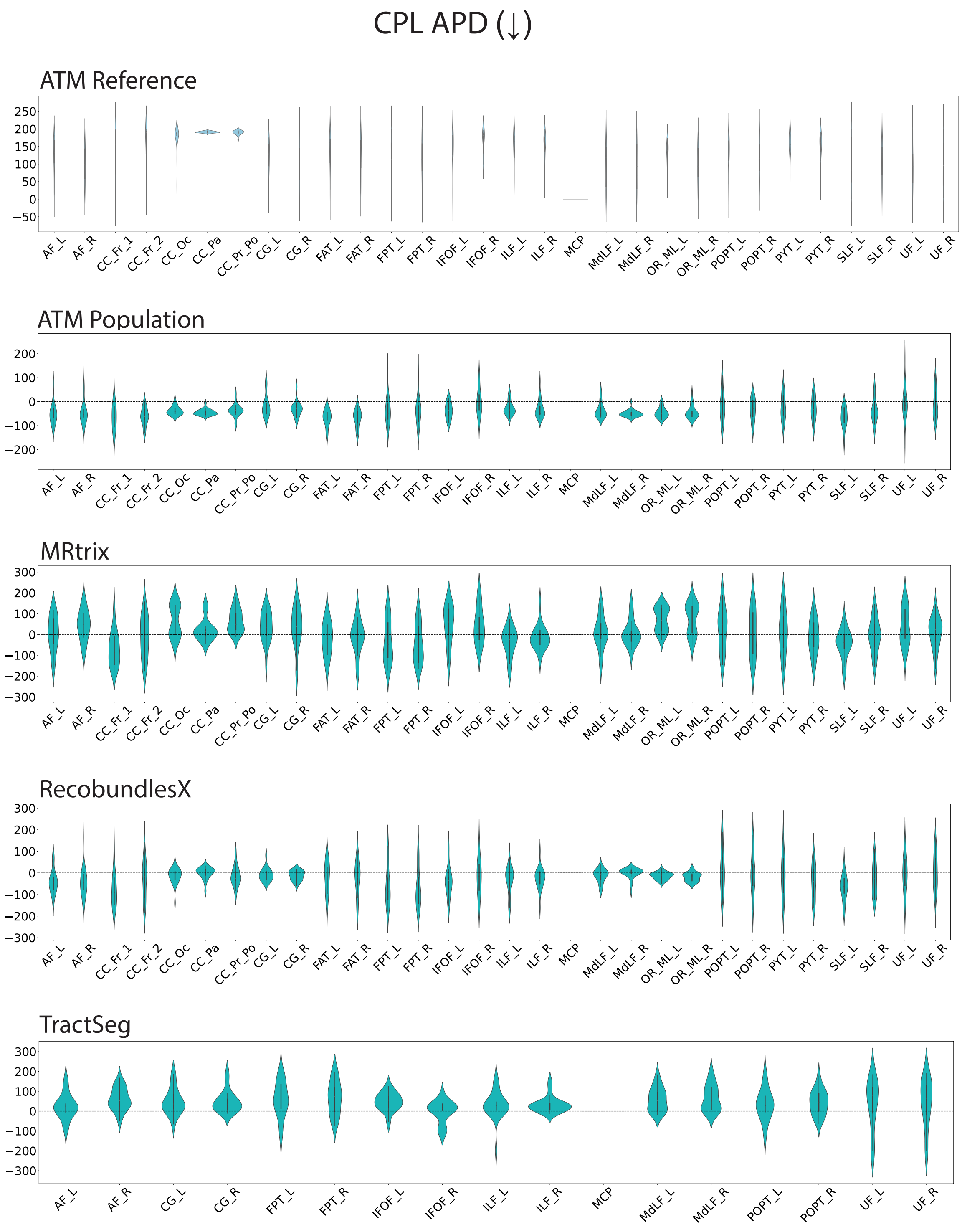

Fig. S24 | Network CPL APD of bundle connectomes generated with ATM, ATM Population, MRtrix, RecobundlesX, and TractSeg. The first violin plot shows the ATM values as the baseline. The subsequent rows display the paired performance differences of other methods relative to this baseline. For each metric, the arrow indicates preference: ↑ higher is better and ↓ lower is better.

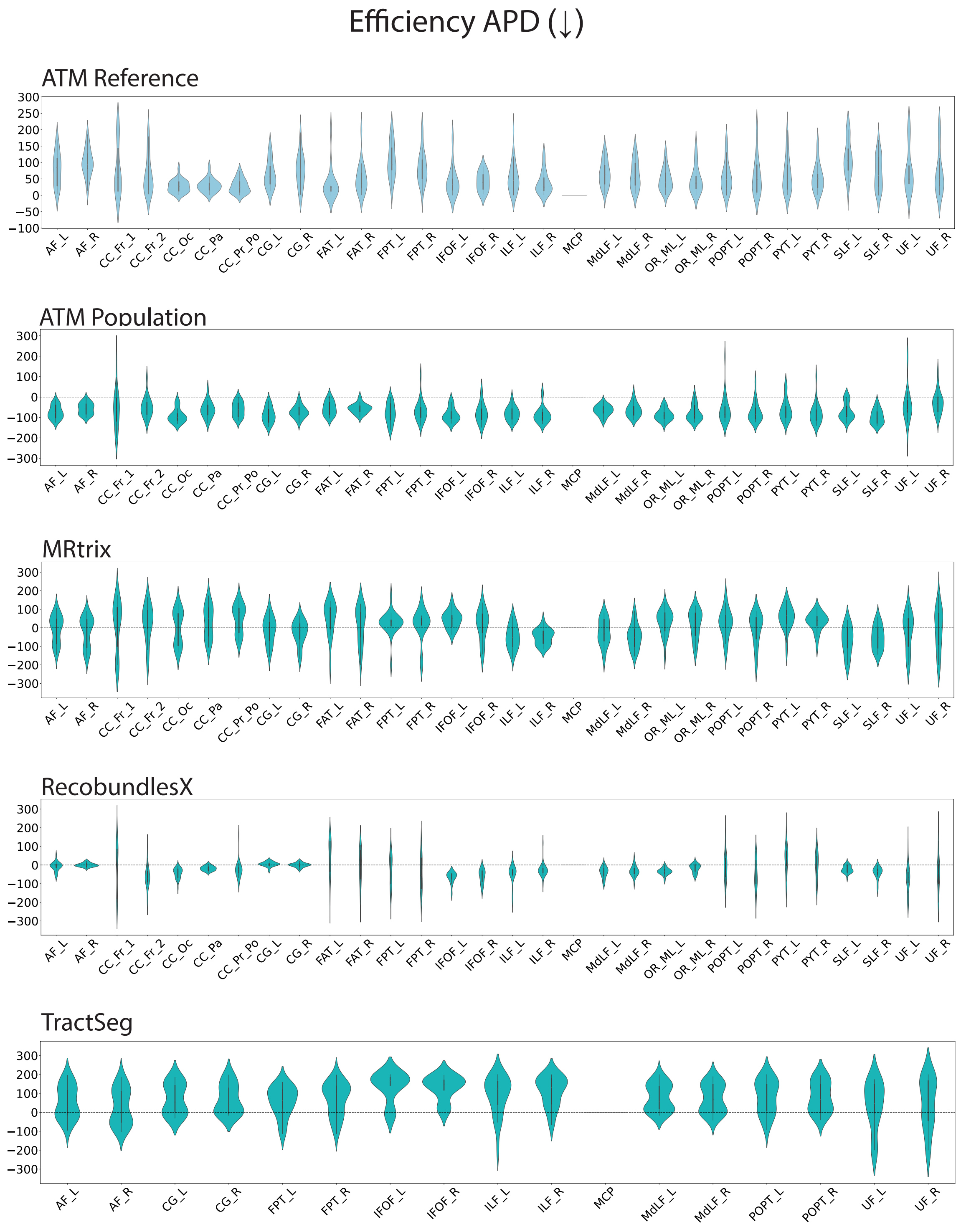

Fig. S25 | Network global efficiency APD of bundle connectomes generated with ATM, ATM Population, MRtrix, RecobundlesX, and TractSeg. The first violin plot shows the ATM values as the baseline. The subsequent rows display the paired performance differences of other methods relative to this baseline. For each metric, the arrow indicates preference: ↑ higher is better and ↓ lower is better.

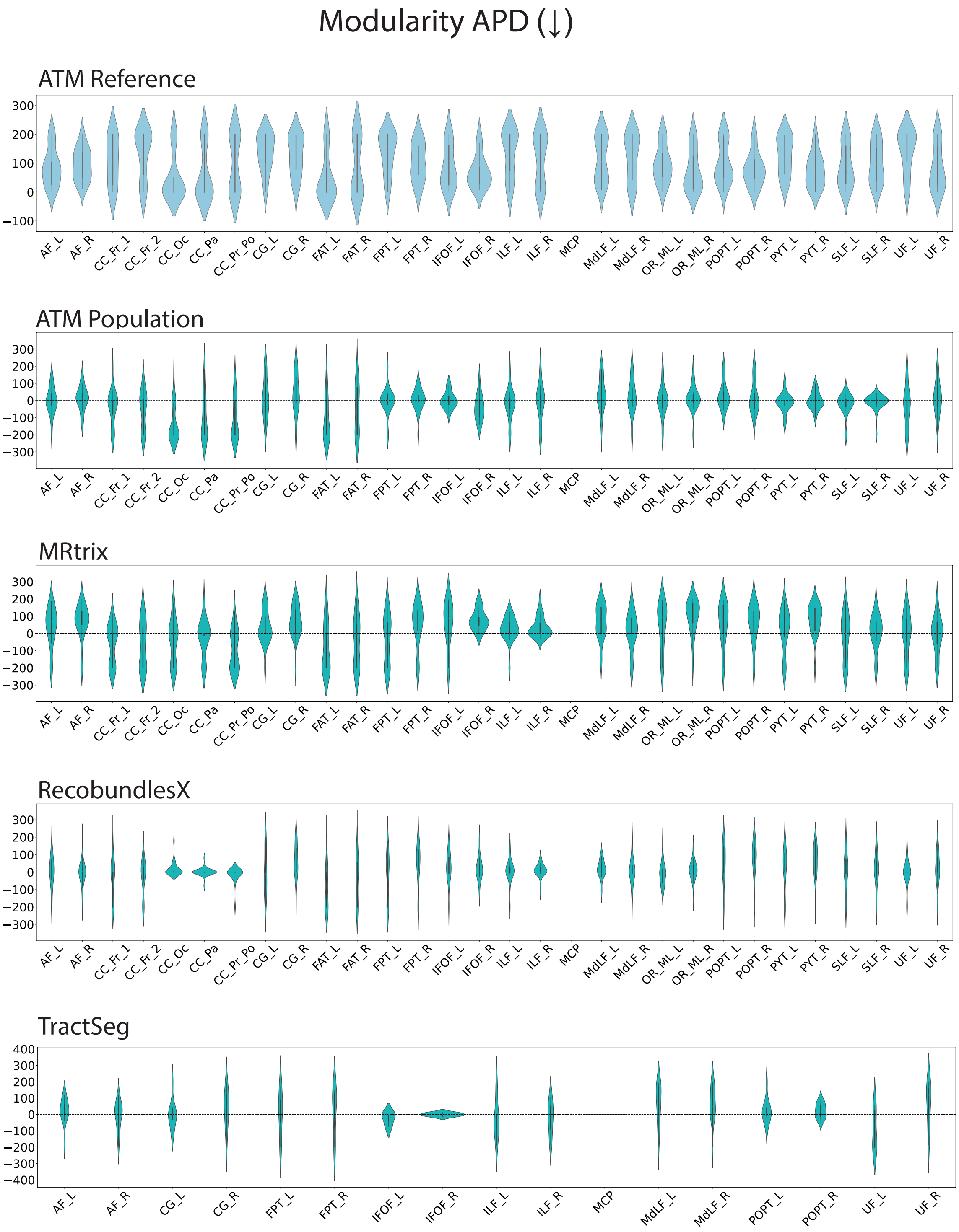

Fig. S26 | Network modularity APD of bundle connectomes generated with ATM, ATM Population, MRtrix, RecobundlesX, and TractSeg. The first violin plot shows the ATM values as the baseline. The subsequent rows display the paired performance differences of other methods relative to this baseline. For each metric, the arrow indicates preference: ↑ higher is better and ↓ lower is better.
